# An Overview of Orchidaceae from Brazil: Advances and Shortfalls After 400 Years of Studies

**DOI:** 10.3390/plants14223520

**Published:** 2025-11-18

**Authors:** Edlley M. Pessoa, Adriane M. Araújo, Felipe F. V. A. Barberena, João A. N. Batista, Adarilda P. Benelli, João S. P. Bento, Eduardo L. Borba, Antônio Edmilson Camelo-Júnior, Patrick C. Cantuária, Letícia W. Cavalcanti, Márlon C. S. Cintra, Mathias Engels, Lucas H. J. Feitoza, Leonardo P. Felix, Alessandro W. C. Ferreira, Cecilia F. Fiorini, Leonardo R. S. Guimarães, Viviane P. Klein, Ana Kelly Koch, Samantha Koehler, Amauri H. Krahl, Dayse R. P. Krahl, Bárbara S. S. Leal, Arthur Macedo, Isabel C. S. Machado, Anna Victoria S. R. Mauad, Juliana L. S. Mayer, Thiago E. C. Meneguzzo, Luiz Menini Neto, Ana Paula Moraes, Paulo Milet-Pinheiro, Felipe Nollet, Eliana M. Oliveira, Miguel S. Oliveira, Emerson R. Pansarin, Fábio Pinheiro, Carla A. Royer, Igor S. Santos, Viviane Silva-Pereira, Eric C. Smidt, Tiago L. Vieira, Luciano R. Zandoná, Danilo Zavatin, Cássio van den Berg

**Affiliations:** 1 Centro de Ciências Naturais e Humanas, Universidade Federal do ABC, Santo André 09280-560, São Paulo, Brazil; 2 Programa de Pós-Graduação em Botânica, Instituto Nacional de Pesquisas da Amazônia, Manaus 69067-375, Amazonas, Brazil; adrianne.maciel.a@gmail.com (A.M.A.); vivianepagnussatklein@gmail.com (V.P.K.); amaurikrahl@hotmail.com (A.H.K.); dayseraiane@hotmail.com (D.R.P.K.); 3 Núcleo de Pesquisas em Epífitas (Nupéfita), Universidade Federal do Rio de Janeiro, Macaé 27930-560, Rio de Janeiro, Brazil; 4 Departamento de Botânica, Instituto de Ciências Biológicas, Universidade Federal de Minas Gerais, Belo Horizonte 31270-901, Minas Gerais, Brazil; janb@icb.ufmg.br (J.A.N.B.); eduardolborba@gmail.com (E.L.B.); ceciliafiorini@gmail.com (C.F.F.); 5 Departamento de Botânica e Ecologia, Universidade Federal de Mato Grosso, Cuiabá 78060-900, Mato Grosso, Brazil; ada.benelli@gmail.com (A.P.B.); luc.jer21@gmail.com (L.H.J.F.); anakbio@gmail.com (A.K.K.); 6 Instituto de Biologia, Universidade Estadual de Campinas, Barão Geraldo, Campinas 13083-970, São Paulo, Brazil; joao.pedrospb@hotmail.com (J.S.P.B.); samk@unicamp.br (S.K.); mayerju@unicamp.br (J.L.S.M.); biopin@unicamp.br (F.P.); 7 Departamento de Ciências Biológicas, Universidade Estadual de Feira de Santana, Feira de Santana 44036-900, Bahia, Brazil; antonioedmilsom@hotmail.com (A.E.C.-J.); vcassio@gmail.com (C.v.d.B.); 8 Instituto de Pesquisas Científicas e Tecnológicas do Amapá, Macapá 68901-025, Amapá, Brazil; patrickcantuaria@gmail.com; 9 Programa de Pós-Graduação em Evolução e Diversidade, Centro de Ciências Naturais e Humanas, Universidade Federal do ABC, Santo André 09280-560, São Paulo, Brazil; letcavalcanti02@gmail.com; 10 Departamento de Química e Biologia, Universidade Estadual do Maranhão, Caxias 65020-000, Maranhão, Brazil; marloncarlos10@gmail.com; 11 Programa de Pós-graduação em Botânica, Departamento de Botânica, Universidade Federal do Paraná, Curitiba 80060-150, Paraná, Brazil; mathiasengels@hotmail.com (M.E.); annavmauad@gmail.com (A.V.S.R.M.); carladriane@gmail.com (C.A.R.); visilvapereira@gmail.com (V.S.-P.); ecsmidt@gmail.com (E.C.S.); 12 Departamento de Fitotecnia, Centro de Ciências Agrárias, Campus III, Universidade Federal da Paraíba, Areia 58397-000, Paraíba, Brazil; lpfelix2@gmail.com; 13 Departamento de Biologia, Universidade Federal do Maranhão, São Luís 65085-580, Maranhão, Brazil; alessandro.wcf@ufma.br; 14 Instituto Nacional da Mata Atlântica, Santa Teresa 29650-000, Espírito Santo, Brazil; leo.rsguimaraes@hotmail.com; 15 Museu Paraense Emílio Goeldi, Coordenação de Botânica, Belém 66040-170, Pará, Brazil; bssleal@gmail.com (B.S.S.L.); miguelsena2010@hotmail.com (M.S.O.); 16 Departamento de Ciências da Vida, Universidade de Coimbra, 3000-456 Coimbra, Portugal; arthur.macedo.rocha@hotmail.com; 17 Departamento de Botânica, Universidade Federal de Pernambuco, Recife 50740-550, Pernambuco, Brazil; isabel.machado@ufpe.br (I.C.S.M.); nolletmedeiros@yahoo.com.br (F.N.); 18 Jardim Botânico do Rio de Janeiro, Rio de Janeiro 22460-030, Rio de Janeiro, Brazil; botanica@meneguzzo.net.br; 19 Departamento de Botânica, Instituto de Ciências Biológicas, Universidade Federal de Juiz de Fora, Juiz de Fora 36036-900, Minas Gerais, Brazil; menini.neto@gmail.com; 20 Laboratório de Citogenômica e Evolução de Plantas, Instituto de Biociências, Universidade Estadual de São Paulo, Botucatu 18618-689, São Paulo, Brazil; 21 Laboratório de Interações Ecológicas e Semioquímicos, Universidade de Pernambuco, Petrolina 56328-900, Pernambuco, Brazil; paulo.milet@upe.br; 22 Laboratório Multiusuário de Microscopia Eletrônica, Universidade Federal da Bahia, Salvador 40110-909, Bahia, Brazil; elianamedeiros.ufba@gmail.com; 23 Departamento de Biologia, Faculdade de Filosofia, Ciências e Letras, Universidade de São Paulo, Ribeirão Preto 14040-900, São Paulo, Brazil; epansarin@ffclrp.usp.br; 24 Departamento de Botânica, Instituto de Ciências Biológicas, Universidade Federal de Goiás, Goiânia 74690-900, Goiás, Brazil; igorsoares@egresso.ufg.br; 25 Instituto Tecnologico Vale—Desenvolvimento Sustentável, Belém 66055-090, Pará, Brazil; tiagolvs@gmail.com; 26 Núcleo de Pesquisas Orquidário do Estado, Instituto de Botânica de São Paulo, São Paulo 04301-902, São Paulo, Brazil; luciano@zandonaconservacao.com.br; 27 Departamento de Botânica, Universidade de São Paulo, São Paulo 05508-090, São Paulo, Brazil; danilozavatin@gmail.com

**Keywords:** anatomy, biogeography, conservation, cytogenetics, evolution, reproductive biology, systematics

## Abstract

The historical background of studies on Brazilian Orchidaceae dates back almost 400 years. In this review, we provide an overview of the current knowledge on Brazilian Orchidaceae across three thematic axes: 1. diversity, distribution, and endemism; 2. taxonomy and systematics; and 3. structural, genetic, and ecological characterization. Brazil harbors five naturalized and 202 native genera, of which 23 are endemic to the country. There are currently 2515 accepted species (out of 9907 species names). Among the 7218 synonyms, 3915 are heterotypic, yielding a synonymy rate of 60.9%. Brazil is the second country in orchid endemism with 1540 endemic species. Apostasioideae is not present in Brazil, but the remaining four orchid subfamilies are represented by 16 tribes and 23 subtribes. The richest phytogeographic domain is the Atlantic Forest (1398 spp.), followed by the Amazon Forest (784 spp.) and Cerrado (656 spp.). The richest subtribes are Pleurothallidinae (642 spp.), Laeliinae (397 spp.), and Oncidiinae (283 spp.). Moving beyond a purely taxonomic and phylogenetic framework, this work offers a comprehensive synthesis of Brazilian Orchidaceae, encompassing the state of the art in cytogenetics, anatomy, population genetics, reproductive biology, and pollination. Despite these advances, there are pronounced disparities among regions, taxa, and research approaches. The persistence of these shortfalls highlights the urgent need for integrative research frameworks. Future progress in Brazilian orchidology depends on the strengthening of collaborative networks and interdisciplinary approaches.

## 1. Introduction

Orchidaceae, comprising around 28,000–31,000 species worldwide [1,2], has long attracted the attention of naturalists and evolutionary biologists such as Charles Darwin [3], due to its remarkable morphological and ecological diversification. Orchids are widely distributed across most continents and more diverse in tropical humid forests [4]. The family stands as the most species-rich plant family in the Americas [5], and it is the second most diverse family in Brazil after Fabaceae [6]. Phylogenetic studies carried out so far have improved orchid classification, resulting in a reasonably stable system with 49 subtribes [7,8,9,10,11,12,13]. However, rearrangements in generic circumscriptions are still being proposed annually, predominantly lumping old genera [14,15,16,17,18]. Consequently, the current taxonomic consensus recognizes between 700 and 736 genera within the family [13], a number subject to ongoing taxonomic changes as genetic and phylogenetic data improve our knowledge about evolutionary relationships.

**Figure 1:**
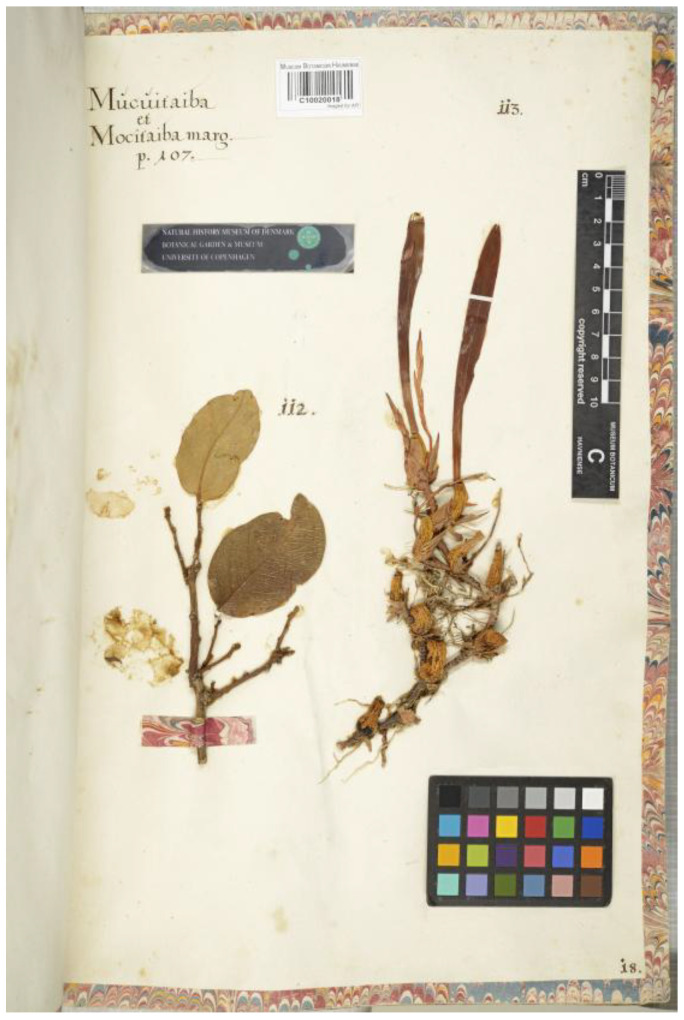
*Maxillaria subrepens* (Rolfe) Schuit. & M.W.Chase collected by Georg Marcgrave in Pernambuco, Brazil. The first orchid collection in the American continent (C-catalogue number: C10020018). Reproduced with permission of the Natural History Museum of Denmark.

The historical background of studies on Orchidaceae in Brazil dates back almost 400 years. With the arrival of Europeans (1500 CE), Brazil was delegated to a peripheral position in global scientific networks and experienced centuries of colonial economic exploitation. Nevertheless, the country maintained a significant, albeit under-recognized, tradition of botanical investigation throughout this period, a fact that contributed to the understanding of its extraordinary floristic diversity. Following the book-herbarium of Gherardo Cibo from 1532 (the earliest extant European botanical collection, currently housed at Bibliotheca Angelica, Rome) [19], Georg Marcgrave collected the first Neotropical plants in Brazil, about a century later (1638–1643), during the Dutch occupation of Pernambuco. This resulted in the creation of the oldest surviving Neotropical book-herbarium, currently held at the herbarium of the University of Copenhagen [20]. Among the specimens collected by Georg Marcgrave, there are some orchids, including *Maxillaria subrepens* (Rolfe) Schuit. & M.W.Chase (herbarium page 18, specimen 113) (Figure 1), and an illustration of a sterile *Catasetum* Rich. ex Kunth [21,22], possibly *C. macrocarpum* Rich. ex Kunth as suggested by Pickel [23]. However, this was a pre-Linnaean publication; hence, both species were later described by other botanists and based on different type materials at the beginning of the 19th century.

In the 18th century, following the Dutch occupation period, Brazil suffered a significant blockade enforced by Portugal, which prevented the entry of foreigners into the country [24]. During this period, between 1783–1792, a Brazilian naturalist born in Salvador, Bahia, named Alexandre Rodrigues Ferreira, explored the North and Midwest Brazil sponsored by the Portuguese Crown. In his Philosophical Journeys (“*Viagens Filosóficas*”), he collected biological samples and ethnographic artifacts during his travels [25,26]. Most specimens did not survive because the collection was looted by Napoleon’s invading troops and sent to France, under the direction of Geoffroy Saint-Hilaire [25,27]. As a result, this disruption caused the irretrievable loss of these valuable botanical materials, or, at least, the loss of any labeling that would have enabled their identification. A single possibly surviving specimen is currently housed in the herbarium of the Muséum national d’Histoire naturelle, Paris, and corresponds to *Elleanthus brasiliensis* (Lindl.) Rchb.f. It is mistakenly labeled as a collection of Domenico Vandelli, who was Ferreira’s professor at Coimbra University and has never visited Brazil. Besides that, other 14 orchid species can be identified among Ferreira’s collections, in the form of high-quality illustrations prepared by Joaquim José Codina and José Joaquim Freire. These are currently housed at the Biblioteca Nacional, Brazil (images available at https://www.brasilianaiconografica.art.br; accessed on 12 November 2025). Except for *Erycina pusilla* (L.) N.H.Williams & M.W.Chase, which had been described some decades earlier by Linneaus [28], all these species would be new species not formally described at the time, but they ended up like Marcgrave’s materials, losing their potential nomenclatural priority. Sir Joseph Banks and Daniel Solander were among the few foreigners allowed to collect specimens around the city of Rio de Janeiro in 1768 [24]. Two of their collections currently housed at the Natural History Museum, London, represent *Epidendrum secundum* Jacq., which was described a few years earlier [29].

**Figure 2:**
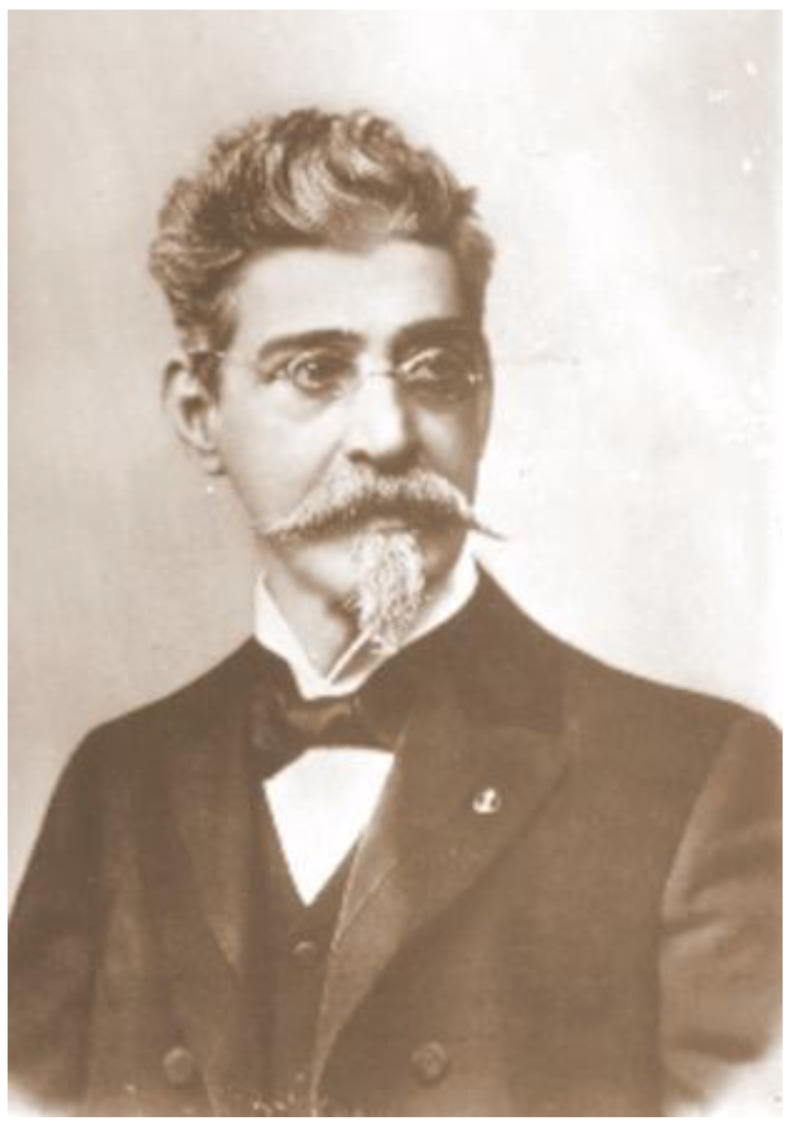
João Barbosa Rodrigues (1842–1909), Brazilian botanist, the most prolific Latin American orchidologist of XIX century. This photograph is made available under the Creative Commons CC0 1.0 Universal Public Domain dedication.

With the advent of the Napoleonic wars, a considerable change happened as a consequence of the transfer of the Portuguese Crown and the capital of the Portuguese Empire to Rio de Janeiro in 1808. The Brazilian ports were opened to other countries and were able to receive foreigners more often, including botanists [26,30]. A survey of the known specimens and contemporary literature indicate that only 18 species of Orchidaceae had been reported for Brazil by that time. This geopolitical transformation facilitated access to Brazil’s territories by foreign naturalists and botanical collectors, initiating a new era in the systematic documentation of Brazilian plant diversity. Georg Heinrich von Langsdorff, a German-Russian naturalist and general consul of the Russian Empire in Brazil, was the main promoter and supporter of expeditions by other naturalists who contributed to the advancement of Brazilian orchidology [31]. Among the prominent collectors of Brazilian orchids in the 19th century, we can list the following: J. Albert C. Löfgren, Anders F. Regnell, Auguste F. M. Glaziou, Auguste F. C. P. Saint-Hilaire, Carl. F. P. von Martius, Eugen Warming, Ernest H. G. Ule, George Gardner, Gustaf Edwall, José M. C. Veloso, Johann B. E. Pohl, Richard Spruce, and William J. Burchell [32]. The first known orchid type specimens collected in Brazil were *Gomesa barbata* (Lindl.) M. W. Chase and N. H. Williams and *Cattleya labiata* Lindl., both collected by William Swainson in Pernambuco and described by Lindley [33,34]. It is also important to highlight the influence of the Empress of Brazil and Queen Consort of Portugal, Leopoldina of Habsburg-Lorraine, who sponsored the inception of the Flora Brasiliensis project, the largest and most comprehensive monograph of a tropical country’s flora at the time [35,36]. Later in the XIX century, João Barbosa Rodrigues (Figure 2), a notable Brazilian naturalist, described over 541 new species and 28 new genera in his publications [30,37,38,39]. In the beginning of the 20th century, Cogniaux [40,41,42] finished his colossal work of monographing Orchidaceae for the *Flora Brasiliensis*, recognizing the presence of 1795 species in Brazil. Noteworthy, 538 of them (ca. 30%) had been described by Barbosa Rodrigues [34].

Following Cogniaux’s treatment, the botanical knowledge of Brazilian orchids expanded rapidly, including the description of new species and the synonymization of names by several Brazilian and non-Brazilian botanists. Among these, Frederico Carlos Hoehne [43,44,45,46,47] and Guido Frederico João Pabst [48,49] were the most significant contributors to the listing and revision of Brazilian Orchidaceae in the 20th century. The latter recognized 2356 species in the country in the two volumes of *Orchidaceae Brasiliensis* [48,49]. Later estimates of the number of species in Brazil during the 20th century were conservative, ranging between 2400–2500 taxa [4,50]. The publication of the Brazilian Catalogue of Plants and Fungi in 2010 reported 2419 orchid species in 235 genera [51], maintaining the taxonomic conservative tradition of Brazilian orchidologists. In this review, we provide an overview of the current knowledge on Brazilian Orchidaceae, highlighting both the advances and the shortfalls in three thematic axes: 1. diversity, distribution, and endemism; 2. taxonomy and systematics; and 3. structural, genetic, and ecological studies.

## 2. Methods: Literature Search Strategy

In this review, we provide a descriptive summary of the main advances in Brazilian orchidology over the past few decades. Most of the data on geographical distribution used for this review comes from the *Flora e Funga do Brasil*, a project initiated in 2008 as a collaborative network of Brazilian and foreign taxonomists [52]. Most authors of this review are also members of this project. This approach of synthesizing and discussing data from *Flora e Funga do Brasil* was also applied for similar reviews of other angiosperm families in Brazil [53,54,55,56,57]. All data on taxa numbers were retrieved from the system in March 2025 based on the digital platform (available at https://reflora.jbrj.gov.br; accessed on 12 November 2025). Additionally, a complementary literature search was carried out using Google Scholar and specialized databases to encompass other related topics, covering studies from the year 2000 up to March 2025 using the keywords “Brazil”, “Orchidaceae”, and “orchids”, and additional keywords specific to each thematic area. The complete list of references gathered is available in Supplementary File S1; surveys of chromosome numbers and studies on pollination focused on Brazilian taxa are available in Supplementary Files S2 and S3, respectively. The conservation status for the species was obtained from the Centro Nacional de Conservação da Flora website (http://cncflora.jbrj.gov.br; accessed on 12 November 2025).

## 3. Diversity, Distribution, and Endemism

**Figure 3:**
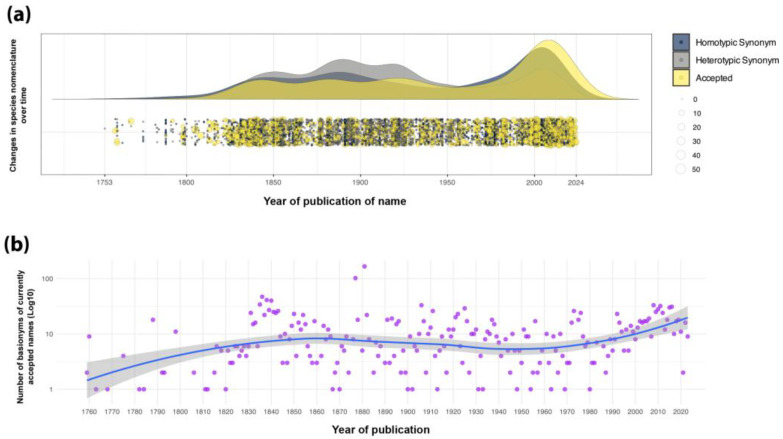
Cumulative dynamics per year (1760–2024) of (**a**) nomenclatural changes (accepted names in yellow, homotypic synonyms in dark grey, and heterotypic synonyms in light grey); and (**b**) accumulation of basionyms of currently accepted species over time.

In Brazil, a megadiverse country, taxonomists have always faced challenges in maintaining accurate species counts, since new species are described monthly [58], while many others are synonymized following careful revisions and synthesis by the active community of taxonomists [52]. Despite the significant progress made in the treatment of the family in the *Flora e Funga do Brazil*, substantial gaps and challenges persist. Some of the largest genera, such as *Habenaria*, *Acianthera*, *Anathallis*, and *Octomeria*, have not yet been revised. There is still a significant amount of unidentified material, or materials with incorrect identifications, both in national and foreign herbaria, as well as in the available online databases. Nevertheless, the data provided by the *Flora e Funga do Brasil* project are taxonomically verified. Most orchid genera have been reviewed and curated by taxonomists, offering conservative, yet feasible, estimates of species numbers. The accepted species occurring in Brazil have been continuously described since 1760 (considering basionyms when applicable, except by *Epidendrum vanilla* L. from 1753). There is an initial peak in the second half of the 19th century associated with Barbosa Rodrigues’ contributions, and a second, more recent peak beginning in the last decade of the 20th century (Figure 3).

Until the preparation of this work, the *Flora e Funga do Brasil* database [6] had accumulated 9907 binomials corresponding to native and naturalized orchid taxa within Brazilian territory, of which 2515 represent currently accepted species (Table 1). Among the total number of binomials, 7218 are synonyms, 3915 of which are heterotypic, resulting in a synonymy rate of 60.9% [i.e., heterotypic synonyms/(accepted names + heterotypic synonyms)], calculated so as to exclude homotypic synonyms (new combinations and basionyms) (Figure 3). When compared with other orchid-rich countries, Brazil ranks as the fourth richest country in species after Ecuador (4187 spp.; [59]), Indonesia (3820 spp.; [60]), and Colombia (3591 spp.; [61]). Considering the extensive Brazilian tropical territory and its status as the repository of the world’s most diverse flora [5,6], one might anticipate a higher orchid species richness than currently documented. This apparent discrepancy may be attributed to the higher elevation variation in these other countries, but also to a historical tendency toward taxonomic lumping in Brazil. Colombia, for instance, has a much lower synonym rate, around 31.9% (6980 names, 3389 synonyms, and 1685 heterotypic synonyms; [2]), which may indicate a tendency toward taxonomic splitting. These numbers reflect a relatively advanced stage of orchid taxonomic knowledge in Brazil, where, in addition to the description of new taxa, taxonomic revisions and integrative studies on species delimitation have also been carried out. This approach allowed Brazilian researchers to address potential taxonomic inflation (an increase in recognized species numbers due to splitting previously defined species into multiple new taxa), an important consideration for global biodiversity assessments [62]. For example, the opposite perspective was applied by the authors of the catalogue of the orchid species of Costa Rica. They explicitly described their method on species acceptance, which includes names with types from the country or immediately adjacent territories [63]. This approach generates an artifact with high endemicity levels and low floristic affinities with neighboring or close countries, such as Mexico or Colombia.

**Figure 4:**
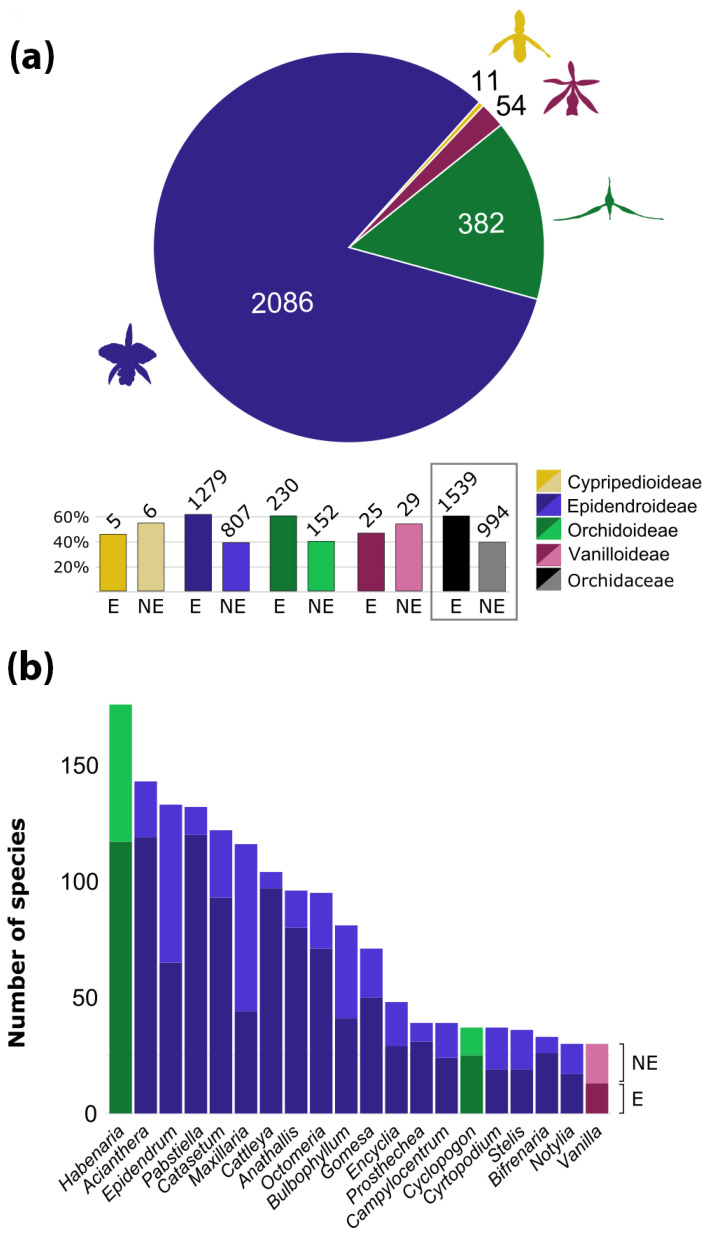
Number of species of Orchidaceae in Brazil organized by (**a**) subfamilies; and (**b**) largest genera in Brazil (E = endemic species, NE = not endemic species).

With respect to generic diversity, Brazil harbors 5 naturalized and 202 native genera of orchids, with 23 genera (approximately 11.1%) endemic to the country [14,15,16,17,18,64]. Worldwide, Brazil is the second richest country in endemic species (1540), after Ecuador (1707 species) [59], followed by Colombia with 1477 [61]. Data for Indonesia is not available. It is also important to highlight that 65% of the species endemic to Ecuador are members of Pleurothallidinae, and another 12% belong to *Epidendrum* L. (Laeliinae), with a similar pattern observed in Colombia [61]. In both countries, many of these endemic species are known only from type specimens and have never been recollected or subject to further investigation. Conversely, the endemic species from Brazil are phylogenetically more diverse. For example, Pleurothallidinae and *Epidendrum* represent only 31.7% and 4.2% of the orchid endemism in Brazil, respectively. Other significant genera with a notable number of Brazilian endemic species are *Habenaria* Willd. (Habenariinae, 7.6%), *Cattleya* Lindl. (Laeliinae, 6.3%), *Catasetum* (Catasetinae, 6%), and *Gomesa* R.Br. (Oncidiinae, 3.2%), which, together with Pleurothallidinae and *Epidendrum*, represent only 59% of the total orchid endemism in Brazil (Figure 4).

**Figure 5:**
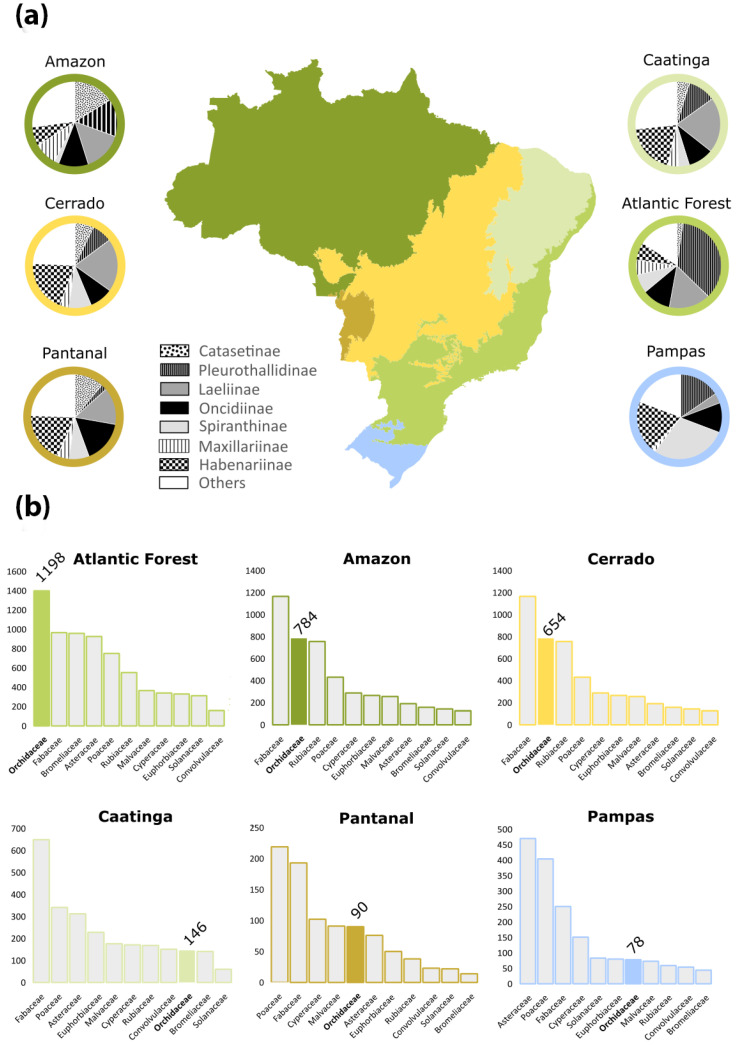
Orchidaceae in Brazilian phytogeographic domains: (**a**) proportion of the diversity organized in the six larger subtribes of the family; and (**b**) position of Orchidaceae considering the most diverse families of Angiosperms by domain.

The Brazilian orchid diversity is not homogeneously distributed across the six main Brazilian phytogeographic domains: Amazon, Atlantic Forest, Caatinga, Cerrado, Pampa, and Pantanal. The Atlantic Forest is the most studied one, but also the most degraded [65], whereas the Amazon Forest is the larger and better preserved [66]. The variety of vegetation types, soil composition, and climatic conditions of each domain are reflected in their orchid diversity, especially in the species numbers, but also in the phylogenetic diversity and substrate preference (e.g. epiphytes, hemiepiphytes, nomadic vines, paludicolous, rupicolous, or terrestrial). The richest phytogeographic domain in orchid species is the Atlantic Forest (1398 spp.), followed by the Amazon Forest (784 spp.) and Cerrado (656 spp.), whereas the other three domains (Caatinga, Pampa, and Pantanal) are much poorer (Figure 5). We summarized the current knowledge of these areas along with future challenges to address existing shortfalls. In the following sections, we detail the patterns of orchid diversity and endemism across the Brazilian six phytogeographic domains, highlighting key patterns and regional challenges.

### 3.1. Amazon Forest

The Amazon Forest is the largest phytogeographic domain of Brazil with a total area of 4,196,943 km^2^ (6,900,000 km^2^ when considering other countries) [67]. It is the most well-preserved domain and considered a wilderness, with huge importance for biodiversity conservation and global water and carbon cycles [68]. The climate is tropical wet or tropical monsoon, and the vegetation is made up of a mosaic of ecosystems and vegetation types, including dense tropical forests (terra firme forests), flooded forests (várzeas and igapós), and open forests that grow on sandy soils, similar to savannas, locally called *campinaranas* and *lavrados* [69].

**Figure 6:**
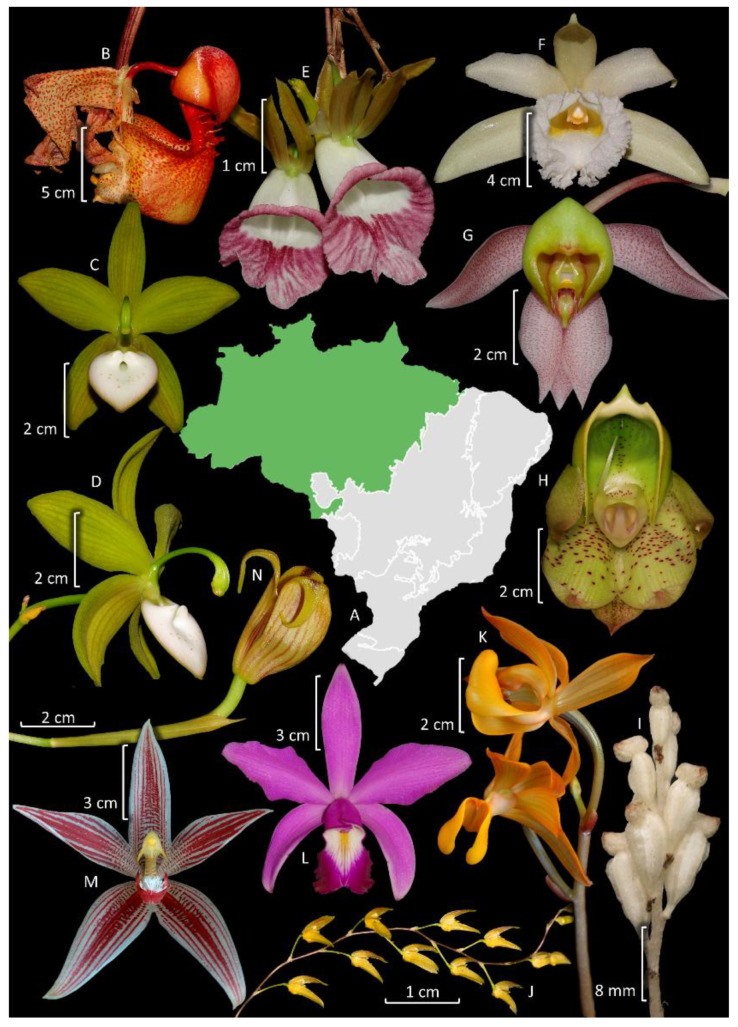
Representative taxa from the Amazon Forest. (**A**) map of the Amazon Forest in Brazil; (**B**) *Coryanthes macrantha* (Hook.) Hook.; (**C**,**D**) *Cycnoches haagii* Barb.Rodr.; (**E**) *Galeandra santarenensis* S.H.N.Monteiro & J.B.F.Silva; (**F**) *Sobralia macrophylla* Rchb.f.; (**G**) *Catasetum matogrossense* Bicalho; (**H**) *Catasetum macrocarpum* Rich. ex Kunth; (**I**) *Wullschlaegelia aphylla* (Sw.) Rchb.f.; (**J**) *Specklinia grobyi* (Batem. ex Lindl.) F.Barros; (**K**) *Mormodes matogrossensis* Engels, Fern.Rocha & E.C.Smidt; (**L**) *Cattleya violácea* (Kunth) Rolfe; (**M**) *Paphinia cristata* (Lindl.) Lindl.; and (**N**) *Maxillaria subrepens* (Rolfe) Schuit. & M.W.Chase. Images’ authorship: (**A**) Tiago Vieira; (**B**–**G**,**K**–**N**) Mathias Engels; and (**H**–**J**) Danilo A. Zavatin.

In the Amazonian domain, Orchidaceae is the second most species-rich family after Fabaceae (Figure 5). Orchids are represented by 134 genera and 784 native or naturalized (2) species, of which 26.8% (210 spp.) are endemic to Brazil, and 517 species occur exclusively in the Amazon Forest (Figure 6). This number closely resembles the estimates of orchid species in the whole Amazon across all countries (769 species) [70]. In that estimate, despite the larger area involved, the authors included only typical lowland species, trying to exclude species from the Andean slopes and Guayana Highlands. Most orchid species are epiphytes, hemiepiphytes, and nomadic vines (608 spp.), whereas 176 are terrestrial, rupicolous, or paludicolous. The richest genera are *Catasetum* (88 spp., including natural nothospecies) (Figure 6H), *Epidendrum* (58 spp.), *Habenaria* (55 spp.), and *Maxillaria* Ruiz & Pav. (49 spp.) (Figure 6N). Among the subtribes, the most representative are Catasetinae (127 spp.; 16.2%), Laeliinae (115 spp.; 14.7%), and Pleurothallidinae (113 spp.; 14.4%) (Figure 5).

Within the Brazilian territory, despite the greater overall floristic richness of the Amazon domain, it harbors only slightly more than half the orchid species diversity documented for the Atlantic Forest domain. This number suggests significant differences in diversification patterns and ecological specialization between these two megadiverse Neotropical forests. The richness difference becomes even more evident considering the fact that the Amazon Forest is approximately 3.2 times larger than the Atlantic Forest [71]. Especially regarding orchids, geomorphological features, such as variations in elevation and soil types (both more variable in the Atlantic Forest), sea influence, and a broader latitudinal range along the Atlantic Forest support a natural explanation for this pattern [72]. However, there is also a strong anthropogenic impact on the Brazilian coast, where 70% of the population lives and the biggest universities and herbaria of the country are located [72]. The low density of scientific collections and the clustered distribution of amazonic species indicate that several parts of the Amazon are still poorly known floristically and that the current knowledge of the species is based on a few, relatively well-collected areas, mainly close to larger urban centers [73]. Despite these limitations, a growing number of taxonomic studies on Orchidaceae have been published in the Brazilian Amazon [74,75] (Supplementary File S1). These studies have revealed a general pattern of low endemism, which can be explained by the shared extension of the Amazon domain with seven neighboring countries. On the other hand, the continuous description of new species points out the Linnean shortfall (the lack of knowledge about the existence and formal description of species), strongly suggesting that a large part of the diversity in this domain could remain unexplored.

Future challenges: Although there have been a number of taxonomic studies on Orchidaceae in the Brazilian Amazon [74,75], the overall research effort remains disproportionately limited relative to its immense territorial expanse. Efforts in developing floristic and taxonomic studies on terrestrial taxa (with the exception of *Habenaria*, [76]) are needed, as the focus has been primarily on epiphytic species. We believe that the epiphytic diversity is under-sampled due to difficulties accessing the higher parts of tree canopies, which can easily reach over 20 m high. Besides that, in the last two decades, an expressive number of new species have been described based only on the type specimens, usually only the holotype, which could be a result of taxonomic inflation, and, therefore, synonymous under other earlier published names from the Brazilian and neighboring Amazonian countries. Additionally, the Darwinian shortfall (the lack of incorporation of taxa in phylogenies; [77]) in the Amazon Forest is also high. Despite the challenges concerning sampling in the region, it is crucial to include taxa from this domain in broader phylogenetic analyses to enhance the understanding of biogeographic patterns in Orchidaceae and Amazon flora. In the age of herbariomics, the possibility of using herbarium specimens for molecular studies has increased significantly [78].

### 3.2. Atlantic Forest

Located on the eastern coast of the country, the Atlantic Forest is the third largest Brazilian phytogeographic domain, covering an area of 1,110,182 km^2^ [71]. At the same time, it is the most degraded phytogeographic domain with only 11% of its original soil cover currently distributed in a small number of forest remnants that hardly exceed 100 ha [65,79]. Recognized as a global hotspot of biodiversity, the domain harbors one of the richest and most endangered forests in the world [80]. The variation on factors such as climate, soil types, elevation, and continentality, combined with its broad latitudinal range (23º), contribute to its remarkable high biodiversity [72]. The vegetation is dominated by different kinds of tropical forests (e.g., rainforests, seasonal forests, and restingas), but also includes rocky outcrops, and altitude grasslands (*campos de altitude*) [69].

**Figure 7:**
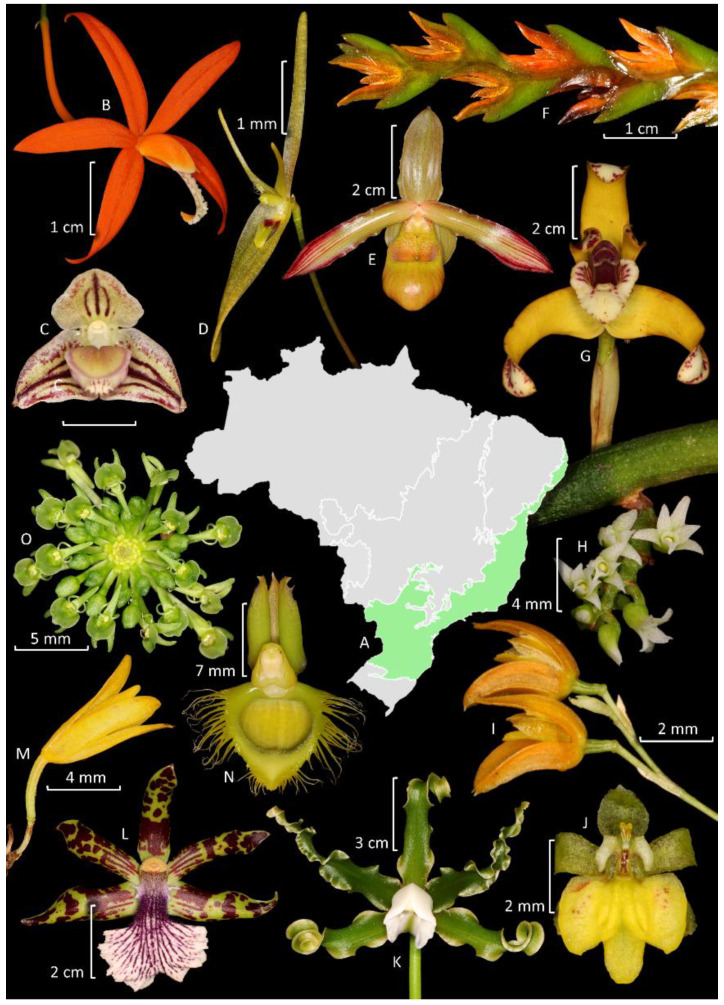
Representative taxa from the Atlantic Forest. (**A**) Map of the Atlantic Forest range in Brazil; (**B**) *Cattleya harpophylla* (Rchb.f.) Van den Berg; (**C**) *Bulbophyllum micropetaliforme* J.E.Leite; (**D**) *Barbosella cogniauxiana* (Speg. & Kraenzl.) Schltr.; (**E**) *Phragmipedium sargentianum* (Rolfe) Rolfe; (**F**) *Acianthera tricarinata* (Poepp. & Endl.) Pridgeon & M.W.Chase; (**G**) *Maxillaria picta* Hook.; (**H**) *Campylocentrum spannagelii* Hoehne*;* (**I**) *Pabstiella trifida* (Lindl.) Luer; (**J**) *Trichocentrum pumilum* (Lindl.) M.W.Chase & N.H.Williams; (**K**) *Vanilla parvifolia* Barb.Rodr.; (**L**) *Zygopetalum crinitum* Lodd.; (**M**) *Octomeria juncifolia* Barb.Rodr.; (**N**) *Catasetum gardneri* Schltr. and (**O**) *Malaxis histionantha* (Link) Garay & Dunst. Images’ authorship: (**A**) Tiago Vieira; and (**B**–**O**) Danilo A. Zavatin.

Orchidaceae is the richest family of plants in this domain (Figure 5), and it is represented by 1398 native or naturalized (4) species belonging to 147 genera, indicating that over half of Brazil’s orchid diversity is found in the Atlantic Forest. Endemism is also high, considering that 1040 orchid species in this domain are endemic to Brazil (74.4%), and 964 species are exclusive to this domain (Figure 7). Most orchid species are epiphytes, hemiepiphytes, or nomadic vines (1063 spp.), whereas 335 are terrestrial, rupicolous, or paludicolous. The richest genera are *Acianthera* Scheidw. (119 spp.) (Figure 7F), *Pabstiella* Brieger & Senghas (108 spp.), *Habenaria* (96 spp.), *Epidendrum* (78 spp.), *Anathallis* Barb. Rodr. (76 spp.), *Octomeria* R. Br. (73 spp.), *Maxillaria* (57 spp.) (Figure 7G), *Gomesa* (56 spp.), and *Cattleya* (52 spp.) (Figure 7B). Among the subtribes, the most representative are Pleurothallidinae with 508 species (36.4%), Laeliinae with 218 species (15.6%), and Oncidiinae with 155 species (11.1%) (Figure 5).

The Atlantic Forest is a major center of diversity for Orchidaceae. Of the 1541 species that are endemic to Brazil, 67.4% are found in this domain. Historical records indicate that the fragmentation of the coastal vegetation was already evident in the 16th century, at least near villages, as shown in Brazilian landscape paintings [81]. Furthermore, considering the fact that most orchids from this domain were only described during the 19th century, it is likely that many species became extinct before they could be formally documented. Although most of the research on taxonomy and systematics in Brazil is carried out in the Atlantic Forest, new species of orchids continue to be described every year (Supplementary File S1). Some big orchid genera present a significant portion of their diversity within this domain, such as *Pabstiella* (ca. 81% of the genus), *Anathallis* (ca. 65%), *Acianthera* (ca. 50%), and *Gomesa* (ca. 47%), providing valuable opportunities for studies on biogeography and diversification. In addition to species belonging to genera primarily centered in the Atlantic region (e.g., *Ornithocephalus* clade [82], and Spiranthinae [83]), there are also many taxa from lineages that are more diverse in the Amazon Forest and the Andes. Amazonian taxa are commonly found in the northern part of the Atlantic Forest, whereas Andean taxa are more prevalent in the Southern portion. These distribution patterns are explained by past connections between these areas that occurred during different geological periods [84,85,86,87], but the biogeographical connection between the Atlantic forest and the Andes had been advocated as early as in the 1960s [88].

Future challenges: The Atlantic Forest is the most well-known phytogeographic domain of Brazil, but there are still many issues that require further studies. Since we have an updated list of species, numerous species complexes have emerged as taxonomic challenges that often remain unaddressed and integrative approaches become necessary to tackle such cases. In some instances, taxonomic inflation is evident, resulting in more names than real species [89], but many cryptic species are also awaiting to be described [90], or simply recognized [91], as several taxa already have formal names that are currently considered synonyms. The potential for novelties is amplified by the fact that the most preserved areas of the Atlantic Forest are steep and remote mountain areas with difficult access, despite being closer to inhabited areas. Moreover, the impact of habitat loss and fragmentation on natural populations of orchids still needs to be further addressed [92], as these populations are often small and sparse [93], and, therefore, are especially susceptible to genetic drift and local extinctions [94].

### 3.3. Caatinga

**Figure 8:**
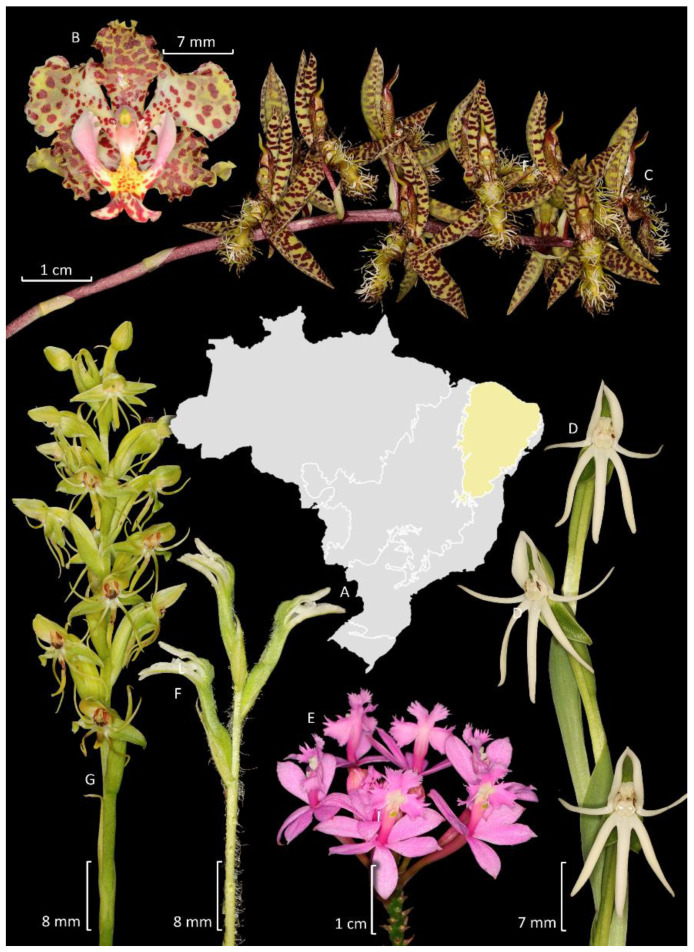
Representative taxa from the Caatinga. (**A**) Map of the Caatinga range in Brazil; (**B**) *Cyrtopodium aliciae* L. Linden & Rolfe; (**C**) *Catasetum barbatum* (Lindl.) Lindl.; (**D**) *Habenaria trifida* Kunth; (**E**) *Epidendrum secundum* Jacq.; (**F**) *Veyretia aphylla* (Ridl.) Szlach.; and (**G**) *Habenaria subviridis* Hoehne & Schltr. Images’ authorship: (**A**) Tiago Vieira; and (**B**–**G**) Danilo A. Zavatin.

The Caatinga is the fourth largest phytogeographic domain of Brazil, covering an area of 850,000 km^2^, primarily located in the northeastern region of the country [71]. The semi-arid climate characterizes this region, but the vegetation consists of a diverse mosaic that includes mainly seasonally dry forests, savannas, and rocky outcrops along with other minor formations [95]. In this domain, the Orchidaceae is comparatively much less diverse than in other domains, being only the ninth richest family after Fabaceae, Poaceae, Asteraceae, Euphorbiaceae, Malvaceae, Cyperaceae, Rubiaceae, and Convolvulaceae (Figure 4). In this review, we did not include in the counting orchid species from areas of the Atlantic Forest and portions of the Espinhaço range that are included under the Caatinga Domain. Only species from other physiognomies that are also characteristic from Caatinga are included. It comprises 146 species organized into 50 genera, from which 51% (75 spp.) are endemic to Brazil, and 14 species are found exclusively in Caatinga (Figure 8). Most species are terrestrial, rupicolous, or paludicolous (79 spp.), whereas 67 spp. are epiphytes, hemiepiphytes and nomadic vines. The richest genus is *Habenaria* (Figure 8D,G) with 29 spp. (Habenariinae, 20%), while subtribe Laeliinae is also well-represented with 30 species (20.5%). Epiphytic species are mostly found in relic forest fragments that survived the expansion of South American dry lands, hence being species found nowadays in the Amazon Forest and/or Atlantic Forest as well.

Most areas of Caatinga are poor in orchid species. However, there are notable exceptions, such as the portions of the Chapada Diamantina (in part, included below as Cerrado since is part of the Espinhaço Range) [96] and the Borborema Plateau [97], even though such higher diversity could be, in part, biased by long-term collection efforts. Taxonomic surveys have been published for several localities in these two geological formations (Supplementary File S1). Despite these localized centers of diversity, knowledge about orchid species in areas outside these regions is scarce. Some exceptions occur in the transition zones between Caatinga and the Atlantic Forest (Supplementary File S1).

Future challenges: Both Chapada Diamantina and Borborema Plateau have populational island systems [98], where disconnected rocky outcrops function as natural barriers to gene flow, resulting in genetically structured patterns [99]. In the long term, this circumstance promotes speciation [100]. However, this process does not always lead to noticeable morphological changes, resulting in the formation of cryptic species [101]. There is evidence of a greater diversity of orchid species in Caatinga, for instance, the exceptional variation in chromosome numbers in *Epidendrum secundum* (Figure 8E) [102] that may indicate the presence of more than one species. The main shortfall in this phytogeographic domain concerns the identification and proper characterization of population-level discontinuities that potentially represent distinct species requiring formal taxonomic recognition.

### 3.4. Cerrado

**Figure 9:**
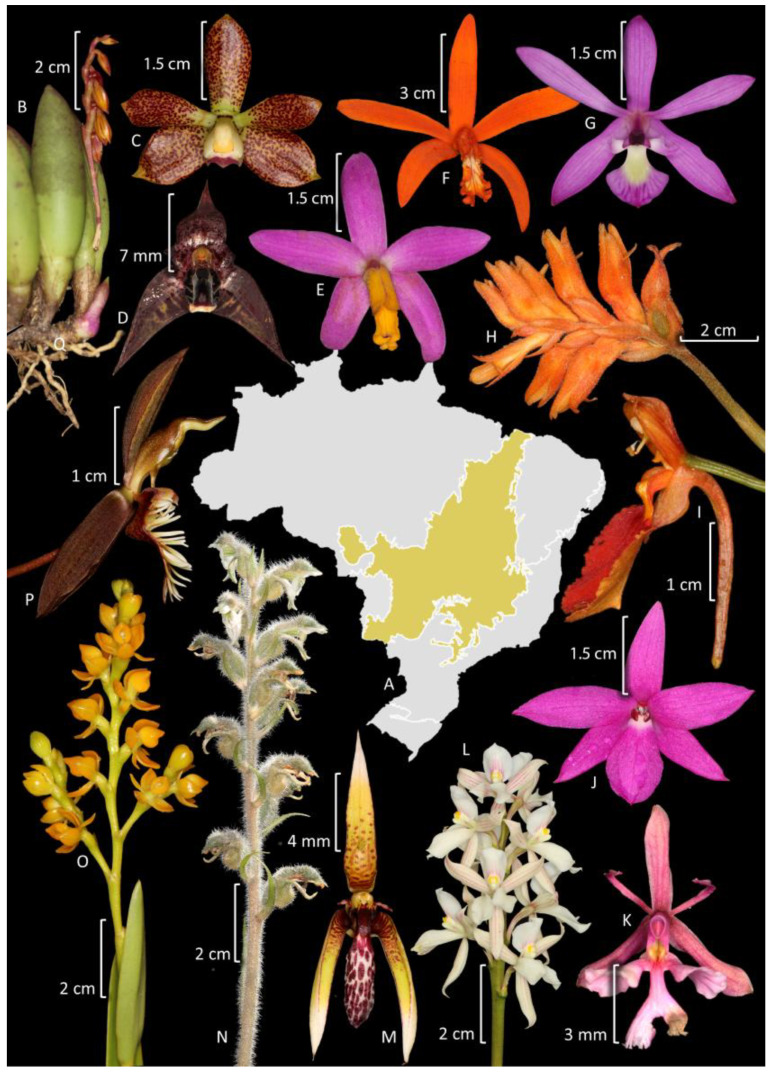
Representative taxa from the Cerrado. (**A**) Map of the Cerrado range in Brazil; (**B**) *Acianthera teres* (Lindl.) Borba; (**C**) *Prosthechea pachysepala* (Klotzsch) Chiron & V.P.Castro*;* (**D**) *Bulbophyllum involutum* Borba, Semir & F.Barros*;* (**E**) *Cattleya rupestris* (Lindl.) Van den Berg; (**F**) *Cattleya cinnabarina* (Bateman ex Lindl.) Van den Berg; (**G**) *Pseudolaelia vellozicola* (Hoehne) Porto & Brade; (**H**) *Sacoila espinhacensis* J.A.N.Bat. & Meneguzzo; (**I**) *Comparettia coccinea* Lindl.; (**J**) *Isabelia violacea* (Lindl.) Van den Berg & M.W.Chase; (**K**) *Epidendrum saxatile* Lindl.; (**L**) *Prosthechea widgrenii* (Lindl.) W.E.Higgins; (**M**) *Bulbophyllum weddellii* (Lindl.) Rchb.f.; (**N**) *Pachygenium parvum* (Cogn.) Szlach., R.González & Rutk.; (**O**) *Epidendrum dendrobioides* Thunb.; and (**P**) *Catasetum lanciferum* Lindl. Images’ authorship: (**A**) Tiago Vieira; and (**B**–**P**) Danilo A. Zavatin.

The Cerrado (Brazilian savanna) is the second largest phytogeographic domain of Brazil in area, covering 2,036,448 km^2^, and is predominantly located in the center of South America, mostly on the Brazilian plateau [71]. Together with the Caatinga and the Chaco formations, the Cerrado constitutes the South American Dry Diagonal, a significant biogeographic corridor that separates the continent’s two major forest biomes, the Atlantic and Amazon forests [103]. The accumulated evidence from phylogeographic investigations indicates that biodiversity in this xeric corridor arose through the interplay of climatic oscillations and geological events, resulting in remarkable levels of diversity and endemism throughout these seasonally dry ecosystems [104]. The vegetation primarily consists of various types of savannas, although semi-deciduous forests and grasslands are also common [105]. It is recognized as the richest savanna in the world and is listed among the global biodiversity hotspots [80]. The higher biodiversity of the Cerrado in relation to the Caatinga and Chaco probably can be explained by the relatively high rainfall, in most parts similar to the average areas of the Atlantic Forest. The open vegetation in the Cerrados is instead explained by a combination of weathered, acidic, and nutrient-poor soils with very low levels of phosphorus [106]. Orchidaceae is the fourth richest family after Fabaceae, Asteraceae, and Poaceae (Figure 5). The family is represented by 653 species organized in 110 genera, from which 59.3% (387 spp.) are endemic to Brazil and 231 species are found exclusively in the Cerrado (Figure 9). Most species are terrestrial, rupicolous, or paludicolous (367 spp.), whereas 287 spp. are epiphytes, hemiepiphytes, and nomadic vines. The richest genera are *Habenaria* with 130 spp. and *Cattleya* with 47 spp.; Habenariinae (20%) and Laeliinae (20.1%) are the most representative subtribes with 131 spp.

The diversity of orchid species in the Cerrado domain appears to be influenced by factors such as the reasonably high rainfall, elevation, and proximity to rivers, as well as a high diversity of soils and vegetation, creating a more diverse set of habitats (Supplementary File S1). The different types of savanna, rocky outcrops, and grasslands present in Cerrado are rich in terrestrial species, but relatively poor in epiphyte species, a pattern compatible with the predominance of open vegetation types, with fewer opportunities for epiphytism. Notably, *Cyrtopodium* R. Br. and *Habenaria* stand out among terrestrial species, since Cerrado is recognized as a center of endemism for both genera [107,108]. When comparing different domains, Cerrado is the richest in terrestrial species (308 spp.), followed by the Atlantic Forest (304 spp.) and Amazon Forest (182 spp.). Semi-deciduous forests, particularly those along rivers, exhibit high diversity in epiphytes; however, they are not as diverse as the communities found in tropical moist forests (Supplementary File S1). Among the richest genera of epiphytes in Cerrado are *Catasetum* (30 spp.) (Figure 9P), *Epidendrum* (28 spp.) (Figure 9K), and *Bulbophyllum* Thouars (20 spp.) (Figure 9D,M).

It is noteworthy that the Espinhaço Range (i.e., Cadeia do Espinhaço, including portions of Chapada Diamantina as cited above), a north–south-oriented mountain chain throughout the states of Minas Gerais and Bahia, harbors a rich orchid flora with many endemic taxa. The characteristic vegetation of the region is the campo rupestre, a savannah-like formation with quartzitic and metasedimentary rocky outcrops, typically found above 900 m [109]. As a result, rupicolous life forms are very common. There is also a specialized orchid flora which is rupicolous on iron-ore, especially in the Southern part of the range. Additionally, semi-deciduous forests occur along rivers and in small patches (capões de mata) scattered throughout the landscape. Most of the area lies within the Cerrado domain, whereas its northernmost and southernmost portions fall within the Caatinga domain (Chapada Diamantina) and the Atlantic Forest, respectively, forming a transition zone between the domains. Since the 1980s, the region has been a target of taxonomic studies, with several floristic studies carried out across its extent (Supplementary File S1). Consequently, the orchid flora of the Espinhaço Range is considerably well-known.

Future challenges: There are numerous published taxonomic studies focused on the Cerrado (Supplementary File S1). However, the taxonomic knowledge remains limited in peripheral areas like the Piauí state, which currently has only 20 recorded species. Similarly, the Cerrados of the states of Bahia, Maranhão, Tocantins, Mato Grosso, and Mato Grosso do Sul are also poorly explored. Some geologically distinctive formations, such as Chapada dos Guimarães in Mato Grosso, harbor noteworthy taxa, including *Chysis guimaraensis* Benelli & E.M. Pessoa and *Lueckelia breviloba* (Summerh. ex E.W.Cooper) Jenny: the former species is the only of the genus outside the Andes and Central America, whereas the latter is a monospecific genus endemic to central South America [110,111]. However, regions of comparable geological uniqueness remain inadequately investigated, including Serra do Amolar, Serra das Araras, Serra de Ricardo Franco in the state of Mato Grosso, and Chapada das Mesas in the state of Maranhão among others. These areas may host more species than are currently known, and could potentially harbor undiscovered taxa. An integrative study summarizing the whole orchid diversity across the whole region is still lacking, in order to provide a revised and taxonomically validated broader checklist, as well as to explore biogeographical patterns, aiming to better understand how this flora was assembled over time and space.

### 3.5. Pampas

**Figure 10:**
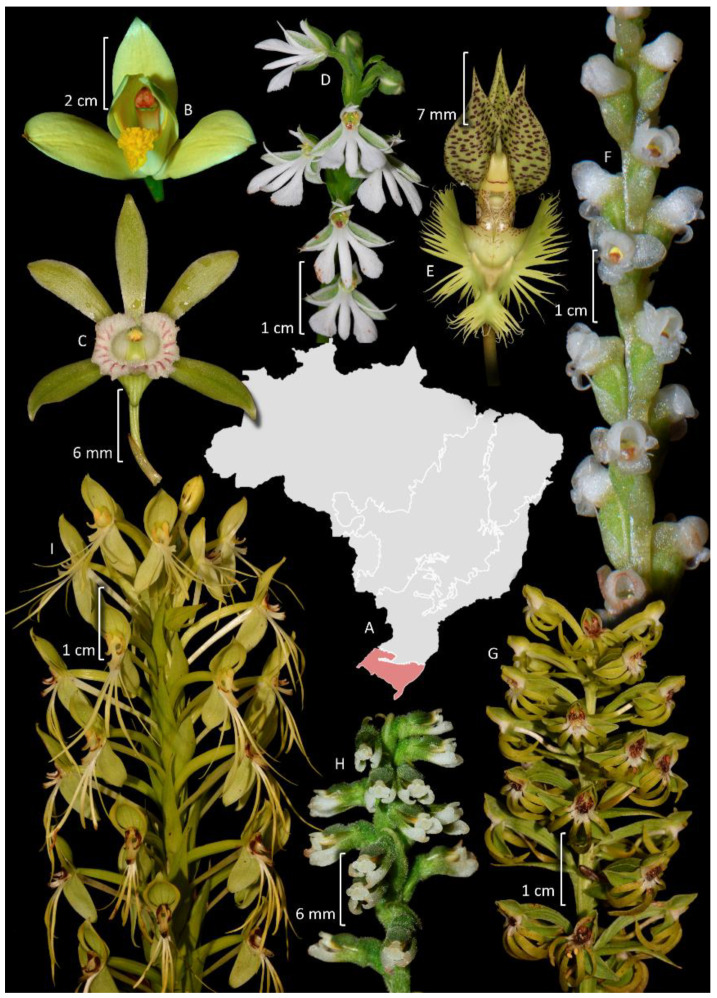
Representative taxa from the Pampas. (**A**) Map of the Pampas in Brazil; (**B**) *Cyanaeorchis arundinae* (Rchb.f.) Barb.Rodr.; (**C**) *Galeandra beyrichii* Rchb.f.; (**D**) *Habenaria leucosantha* Barb.Rodr.; (**E**) *Catasetum fimbriatum* (C.Morren) Lindl.; (**F**) *Prescottia oligantha* (Sw.) Lindl.; (**G**) *Habenaria achalensis* Kraenzl.; (**H**) *Cyclopogon apricus* (Lindl.) Schltr.; and (**I**) *Habenaria macronectar* (Vell.) Hoehne. Images’ authorship: (**A**) Tiago Vieira; (**B**,**D**,**F**–**I**) João A.N. Batista; and (**C**,**E**) Danilo A. Zavatin.

The Pampas constitute the second smallest phytogeographic domain of Brazil, occupying approximately 176,000 km^2^, restricted within Brazilian territory to the state of Rio Grande do Sul, while extending beyond national boundaries to Argentina and Uruguay [71]. It is located in the South Temperate Zone and has both subtropical and temperate climates with four well-characterized seasons. The Pampas comprise a combination of vegetation types, covered predominantly by grassland vegetation, shrub formations, and sparse forest formations [112]. Although it holds approximately 9% of the Brazilian biodiversity, the Pampas are neglected in terms of conservation, and knowledge of its biodiversity is fragmented, lacking taxonomic knowledge and information on species distribution for many regions of the state [112]. Orchidaceae is the sixth largest family in terms of species diversity, following Asteraceae, Poaceae, Fabaceae, Cyperaceae, and Solanaceae (Figure 5). It is represented by 78 species organized in 31 genera, of which only 17 are endemic to Brazil and six are exclusive to the Pampas. Most species are terrestrial, rupicolous, or paludicolous (48 spp.), whereas 30 are epiphytes. The richest genus is *Habenaria* with 15 spp. (Figure 10D,G,I); Habenariinae (19,2%) and Spiranthinae are the most representative subtribe with 23 spp. (29.5%). Other highlights are Chloraeinae (five spp.) and Codonorchideae (one spp.).

Future challenges: Few localities in the Brazilian Pampas have been studied for taxonomic purposes. Most of the existing knowledge of this domain regarding orchid flora comes from taxonomic treatments focused on selected genera within the state of Rio Grande do Sul (Supplementary File S1). The current number of orchid species recorded in the Brazilian portion of the Pampas may be underestimated, as surrounding countries have higher diversity in this domain [113,114]. Future taxonomic revisions must consider the names in use and which are applied to the floras of the surrounding countries (Argentina and Uruguay), as many names currently used in Brazil are likely to be synonyms of those from those areas.

### 3.6. Pantanal

**Figure 11:**
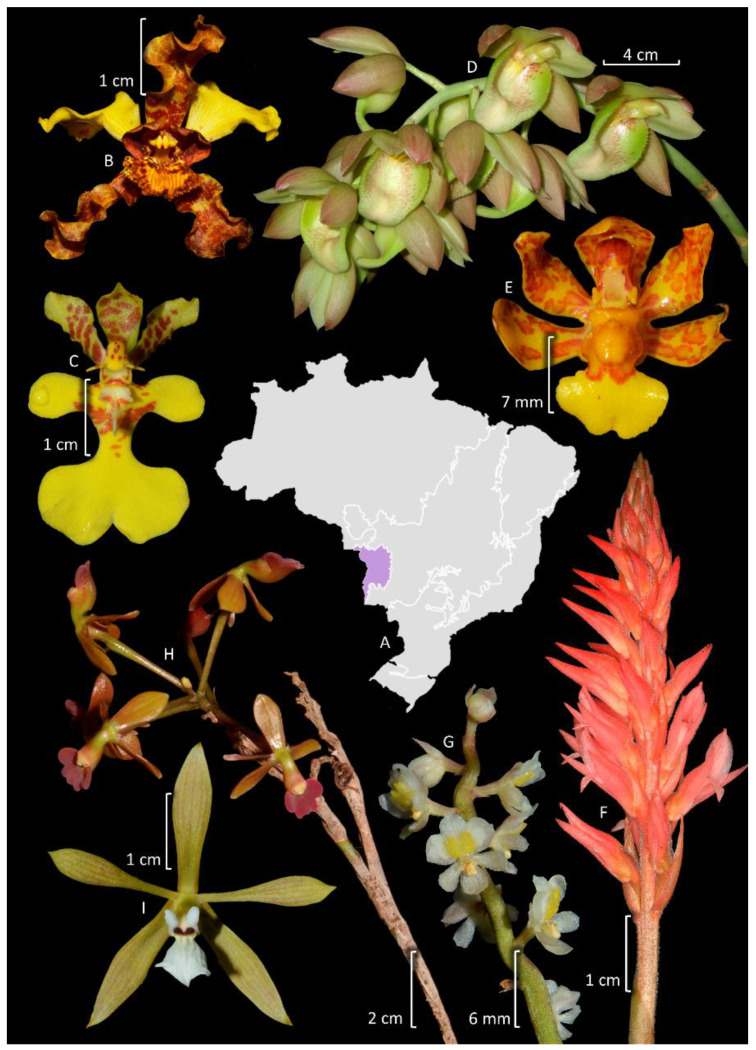
Representative taxa from the Pantanal. (**A**) Map of the Pantanal range in Brazil; (**B**) *Cyrtopodium saintlegerianum* Rchb.f.; (**C**) *Trichocentrum cepula* (Hoffmanns.) J.M.H.Shaw; (**D**) *Catasetum vinaceum* (Hoehne) Hoehne; (**E**) *Trichocentrum nanum* (Lindl.) M.W.Chase & N.H.Williams; (**F**) *Sacoila lanceolata* (Aubl.) Garay; (**G**) *Trichocentrum morenoi* (Dodson & Luer) M.W.Chase & N.H.Williams; (**H**) *Epidendrum anceps* Jacq.; and (**I**) *Encyclia linearifolioides* (Kraenzl.) Hoehne. Images’ authorship: (**A**) Tiago Vieira; (**B**,**C**,**E**,**G**,**I**) Adarilda P. Benelli; (**D**) Ana K. Koch; (**F**) Danilo A. Zavatin; and (**H**) João N.A. Batista.

The Pantanal is the smallest phytogeographic domain of Brazil, covering an area of 150,355 km^2^, and is situated on the Brazilian border with Bolivia and Paraguay in the Mato Grosso and Mato Grosso do Sul states [71]. It is a wetland belonging to the categories of alluvial and fluvial floodplains, in which the flood pulse is predictable, monomodal, and of low amplitude. It is composed of a vegetation mosaic of flooded grasslands, savannas, and forests forming different macrohabitats [115]. Although the domain is considered the world’s largest floodplain, its flora remains one of the least studied in Brazil [116]. Indeed, this is the only phytogeographic domain for which the data from the *Flora e Funga do Brazil* are almost absent. Given this issue, we provide an updated checklist of Orchidaceae from Pantanal in Supplementary File S4. The family is the fifth richest family in species, after Poaceae, Fabaceae, Cyperaceae, and Malvaceae (Figure 5). The family is represented by 87 species organized into 39 genera, of which 16.7% (15 spp.) are endemic to Brazil, even though none are exclusive to the Pantanal (Figure 11). Most species are epiphytes, hemiepiphytes, and nomadic vines (52 spp.), while 38 spp. are terrestrial, rupicolous, or paludicolous. The richest genus is *Habenaria* with 17 spp. (Habenariinae, 23%). Members of Oncidiinae and Laeliinae also represent significant groups with 30 (34.5%) and 13 spp. (14.9%), respectively.

Although smaller in size, the Brazilian Pantanal is richer in species than the Pampas. Nevertheless, it warrants emphasis that the paucity of endemic taxa may suggest this domain does not function as a significant center of diversification for Orchidaceae, though this interpretation is constrained by limited sampling and taxonomic investigation within the region. Most species are widespread, with only a few being endemic to Brazil. The flooding regime may favor some *Habenaria* species, but, even among these, the five Brazilian endemic ones are widely distributed in central Brazil. It seems that Pantanal can bear some Amazonian and Cerrado species that tolerate its warm climate and intermittent flood conditions.

Future challenges: The Wallacean shortfall (the lack of knowledge about the geographic distribution of species) in Pantanal is significant, because its flora is poorly collected and documented. The only major botanical expedition in this domain took place in the early 20th century (1908–1923) by [47]. There are currently no taxonomic studies specifically focused on Orchidaceae in this region, aside from broader checklists for the states of Mato Grosso and Mato Grosso do Sul [117,118]. The number of specimens available in herbaria is minimal and the amount of those with taxonomic identification done or verified by experts is also very little. Therefore, larger collection efforts are among the first steps towards better knowledge of the orchids from the Pantanal domain.

## 4. Taxonomy and Systematics

The current widely accepted classification system for Orchidaceae includes 5 subfamilies, 22 tribes, and 49 subtribes [13]. Recent phylogenetic studies using high-throughput genomic datasets, comprising hundreds of loci, confirmed these groups and provided better support for their relationships [119,120]. Brazil holds four of the subfamilies, except for Apostasioideae which is restricted to Asia and Oceania [7]. The distribution of Apostasioideae suggests a putative Paleotropical origin and limited dispersal beyond this region. Cypripedioideae and Vanilloideae are not well-represented, except for the genus *Vanilla* Plum. ex Mill. (Figure 7K) which includes 30–40 species [121]. The greatest diversity is found in subfamilies Epidendroideae (2063 spp.) and Orchidoideae (387 spp.). Among the tribes, 16 are present in Brazil (73% of the total), with the most representative being Epidendreae (1046 spp.), Cymbidieae (818 spp.), Cranichideae (206 spp.), and Orchideae (180 spp.). There are 25 subtribes represented in Brazil (51% of the total), with the richest in species being Pleurothallidinae (642 spp.), Laeliinae (397 spp.), Oncidiinae (283 spp.), Catasetinae (186 spp.), Habenariinae (180 spp.), Maxillariinae (159 spp.), and Spiranthinae (154 spp.). Regarding endemism to Brazil, the following subtribes have the highest proportions: Pleurothallidinae (75.4%), Catasetinae (68.5%), Laeliinae (66%), and Habenarinae (65%); this highlights the significant endemism within these taxa (Table 1).

The level of taxonomic knowledge of Orchidaceae in Brazil is unevenly distributed across different taxa. For instance, the species of Laeliinae are much better studied than those of Pleurothallidinae. Moreover, the phylogenetic relationships of several genera of Oncidiinae remain unclear [122], and the largest genera of Catasetinae still lack taxonomic revisions. In the latter, the number of species and nothospecies described has grown two times in the last three decades without any proper revision. Below, we summarize the studies that have been carried out on Brazilian Orchidaceae including checklists, floras, taxonomic revisions, and phylogenetic reconstructions, as well as the main shortfalls that need to be addressed in future research.

### 4.1. Cypripedioideae and Vanilloideae

Cypripedioideae, commonly known as “slipper orchids”, includes five genera widespread in temperate and tropical regions of Eurasia and America [7]. Among these genera, four are present in the Neotropics, but only two are recorded in Brazil, *Phragmipedium* Rolfe (seven spp., two endemic to the country) and *Selenipedium* Rchb. f. (four spp., two endemic to the country). Although the flower shapes of these two genera are similar, they are easily distinguished by their leaves, conduplicate in the former, plicate in the latter [7]. Except for *P. sargentianum* (Rolfe) Rolfe (Figure 7E) and *P. vittatum* (Vell.) Rolfe, which are well-documented with several specimens in the herbaria, the other species are rare, and with some known only from their type specimens. While they are uncommon, members of Cypripedioideae are widely distributed in Brazil, particularly outside of the southern region. It is likely that new occurrences will be found for *Phragmipedium* in the bordering states of the Amazon domain.

Vanilloideae is widespread in tropical regions and it is organized into two tribes, Pogonieae and Vanilleae [9], both represented in Brazil. Pogonieae includes four genera, two of which are present in Brazil, *Cleistes* Rich. Ex Lindl. (15 spp., 11 endemic to the country) and *Duckeella* Porto & Brade (3 spp.). The first is widespread in the country, whereas the last is narrowly endemic to a portion of the Guyana shield that includes parts of Brazil, Colombia, and Venezuela [9]. Notably, *Cleistes* is particularly rich in species in the Cerrado, where 12 species occur, accounting for about 40% of the whole genus. Vanilleae includes nine genera, but only two are recorded in Brazil, *Epistephium* Kunth (6 species, 1 endemic to the country) and *Vanilla* (30–40 species including nothospecies, 13 endemic to the country). Both genera are widespread throughout Brazil, but *Epistephium* is richer in the Amazon (five spp.) and Cerrado (four spp.), whereas *Vanilla* is more diverse in the Amazon (15 spp.), Atlantic Forest (13 spp.), and Cerrado (11 spp.). Taxonomic studies on Vanilloideae in Brazil focus on the expansion of species distribution, the discovery of new species (Supplementary File S1), or, less commonly, the use of multidisciplinary approaches for species delimitation [123]. Presently, there is a lack of published taxonomic treatments for *Epistephium* and *Duckeella*, and there is an ongoing taxonomic revision for *Vanilla*.

Future challenges: Currently, there is no comprehensive taxonomic study on the Brazilian species of Cypripedioideae. The actual number of species may be underestimated because the quality of the herbarium specimens is often poor, which hampers proper identification. Additionally, populations of *P. sargentianum* are significantly variable, suggesting that this species may, in fact, represent more than one distinct species. To gain a better understanding of these species, further research in the alpha taxonomy and population genetics is necessary. Conversely, there are several punctual studies on Vanilloideae species from Brazil, particularly focusing on *Vanilla* (Supplementary File S1). This genus has currently been studied by several research groups in the Neotropics. Some studies synonymized a number of Brazilian species based on the examination of photographs of type specimens [124]. However, accurate investigations based on integrative taxonomy have been used to revalidate some species. It is the case of *V. lindmaniana* [125], *V. calyculata* [126], and *V. argentina* [127]. In addition, many endemic species have been published [121,128] or rediscovered [129,130]. There is an unpublished thesis with a taxonomic revision for *Cleistes* [131], but none for *Epistephium*. Aside from formal revisions, given the complexity of species delimitation in Vanilloideae [123,125,127], this taxonomic group seems to require the application of integrative approaches. The species diversity within Vanilloideae is likely underestimated because the taxonomic treatments are primarily based on dried specimens.

### 4.2. Spiranthinae

Spiranthinae is the largest subtribe of Cranichideae, predominantly found in the Neotropics [9], and it encompasses 40 genera and approximately 520 species [83]. According to Salazar et al. [83], there are four major clades, namely, *Eurystyles*, *Pelexia*, *Spiranthes*, and *Stenorrhynchos*, all of which are represented in Brazil. Brazil hosts 24 genera and 154 species, which accounts for about 30% of the total, with 90 species being endemic to the country. The subtribe is almost entirely terrestrial, although two genera, *Eurystyles* Wawra and *Lankesterella* Ames, are exclusively epiphytes, along with some species of *Cyclopogon* C.Presl. (Figure 10H). The subtribe is more diverse in the Atlantic Forest (104 spp.), followed by Cerrado (56 spp.) The most representative genera are *Cyclopogon* (37 spp.), *Sarcoglottis* C. Presl. (13 spp.), *Eltroplectris* Raf. (11 spp., ca. 65% of the total of the genus), *Eurystyles* (11 spp.), *Pachygenium* (Schltr.) Szlach, R. González & Rutk. (11 spp.) (Figure 9N), and *Brachystele* Schltr. (10 spp.). Additionally, there are also some endemic genera such as *Nothostele* (two spp.), which is restricted to the Atlantic Forest, and *Espinhassoa* Salazar & J.A.N.Bat. (two spp.), from Cadeia do Espinhaço in eastern Brazil [132]. Of particular interest is the monotypic genus *Cotylolabium*, with a single species, *C. lutzii* (Pabst) Garay, that is microendemic to the Serra do Caparaó. It is known only from very small populations near Pico da Bandeira, one of the highest peaks in Brazil. This genus is the sister group to the remaining Spiranthinae and possesses very peculiar morphological characteristics, shedding light on the group’s evolution and biogeography [133]. Taxonomic studies on Spiranthinae in Brazil primarily focus on descriptions of new species, nomenclatural issues [134], and species distribution expansions (Supplementary File S1), along with phylogenetic studies [132,133,135].

Future challenges: There are important gaps in the understanding of the phylogenetic relationships among several genera in Spiranthinae—specifically, in the clade that includes *Thelyschista* Garay, *Buchtienia* Schltr., *Nothostele* Garay, *Eltroplectris*, *Mesadenella* Pabst & Garay, *Pteroglossa* Schltr., *Lyroglossa* Schltr., *Sacoila* Raf. (Figure 9H), and *Skeptrostachys* Garay [83]. Previous attempts to resolve these relationships using small sets of Sanger-sequenced DNA regions have proven unsuccessful. Therefore, the use of complete plastid genomes [15] and other high-throughput genomic datasets may be a more effective approach. Furthermore, generic delimitation in this clade is also not simple, and some lumping would be beneficial for the orchid community. The Brazilian species of *Brachystele*, *Cyclopogon*, *Pachygenium*, *Pelexia*, *Sacoila*, *Skeptrostachys*, and *Sarcoglottis* are not well-delimited or -studied, and the current richness seems to be overestimated, and the genera lack proper revisions. A comprehensive taxonomic treatment, coupled with phylogenetic analyses for these genera, is necessary, ideally including *Veyretia* (Figure 8F), since it has been found to be nested within *Cyclopogon* [83,136,137]. The Wallacean and Linnean shortfalls in the Amazon Forest are significant, since only 13 species of Spiranthinae have been recorded to this domain so far. A focused study may provide a better overview of the subtribe in this region.

### 4.3. Other Orchidoideae

In Brazil, the other Orchidoideae, excluding Spiranthinae, are represented by the tribe Codonorchideae (1 spp.), and subtribes Chloraeinae (5 spp.), Cranichidinae (19 spp.), Goodyerinae (27 spp.) (Cranichideae), Habenarinae (180 spp.) (Orchideae), and Discyphinae (1 spp.). Among these taxa, *Habenaria* (180 spp.) stands out as one of the richest genera of Brazil (Figure 4b), and the only representative of Orchideae in the country. It is also one of the largest genera of monocots with over 898 species distributed along the tropical and temperate zones of the world [2]. *Habenaria* has been extensively studied in Brazil, with research covering taxonomic treatments [76,138,139,140], and molecular phylogenetics [141,142,143], as well as integrative studies for species delimitation [144].

The only known species of Codonorchideae from Brazil, *Codonorchis canisioi* Mansf., was recently rediscovered after 78 years since its original description [145]. Goodyerinae has undergone substantial taxonomic recircumscription in the last years, and most of its previously recognized native genera were synonymized into *Microchilus* C. Presl. [15]. The Brazilian species were revised in a thesis, but not yet published [146]. An exception is the invasive *Zeuxine strateumatica* (L.) Schltr., a species native to Asia [147]. There is a published revision of *Prescottia* Lindl. (Cranichidinae) (Figure 10F) [148] and another for Chloraeinae [149]. A study on the phylogenetic position of *Discyphus scopulariae* (Rchb.f.) Schltr., based on materials collected in Bahia, Brazil, revealed that it corresponds to a monotypic genus in an isolated subtribe, Discyphinae Salazar & van den Berg [150]. However, many other taxa remain less studied.

Future challenges: As described above, Orchidoideae is relatively well-studied in Brazil. However, except for *Habenaria*, little is known about species from the Amazon Forest. Apart from *Baskervilla* Lindl., *Ponthieva* R. Br., and *Stenoptera* C. Presl., which each have only one species represented in Brazil, the other genera need to be better studied. The Brazilian *Cranichis* Sw. (four species) have never been subject to a particular revision, and only one species has been sampled in previous phylogenetic studies, suggesting that the diversity of this genus may be underestimated. Although *Habenaria* is well-studied, a comprehensive and updated review of the genus is needed for the country, while several species complexes among Brazilian species require more in-depth research.

### 4.4. Epidendreae

Epidendreae is organized in six subtribes [13], of which five, Bletiinae (2 spp.), Calypsoinae (1 spp.), Laeliinae (397 spp.), Pleurothallidinae (642 spp.), and Ponerinae (2 spp.), are represented in the Brazilian flora. It is also the most diverse tribe in Brazil, comprising 1043 spp., and exhibits extremely high endemism, particularly in its richest subtribes. Bletiinae is represented by two species, *Bletia catenulata* Ruiz & Pav., which is widespread throughout South America, and *Chysis guimaraensis*, which is micro-endemic to western Brazil [111]. *Chysis* was previously thought to be restricted to the Andes and Central America, but the discovery of this species in Brazil has expanded its known distribution to mid-elevations (~600 m). *Bletia stenophylla* (Schltr.) occurs very near the Brazilian border in the Venezuelan Gran Sabana, and probably will be found in Roraima with more sampling effort.

Calypsoinae is represented by *Govenia utriculata* (Sw.) Lindl., but the taxonomic status of the Brazilian populations of this species requires clarification, as Salazar et al. [151] indicate that this name should be applied only to the Antilles and Mexico. Ponerinae is represented by one widespread species, *Isochilus linearis* (Jacq.) R.Br., and the Brazilian endemic *Nemaconia australis* (Cogn.) Van den Berg.

Laeliinae is restricted to the Neotropics, being the second richest subtribe in Brazil [10]. Recent phylogenetic studies have redefined several taxa, resulting in a current total of 38 genera [13,152], of which 20 are recorded in Brazil, and 5, *Adamantinia* Van den Berg & C.N. Gonç. (one spp.), *Constantia* Barb. Rodr. (six spp.), *Homalopetalum* Rolfe (two spp.), *Pseudolaelia* Porto & Brade (12 spp.) (Figure 9G), and *Pygmaeorchis* Brade (two spp.), are endemic to the country. Endemicity is also high in *Leptotes* Lindl. (71%). The most representative genera are *Epidendrum* (130 spp.) (Figure 8E, Figure 9O,K, and Figure 11E) and *Cattleya* (106 spp.) (Figure 6L, Figure 7B, and Figure 9E,F). The subtribe is more diverse in the Atlantic Forest (218 spp.), followed by Cerrado (131 spp.). It is one of the better studied subtribes in Brazil, encompassing studies of alpha taxonomy, molecular phylogenies, and multidisciplinary studies focused on species delimitation (Supplementary File S1). Among the relatively diverse genera in Brazil, there is a recent phylogenetic study of *Prosthechea* Knowles & Westc. (Figure 9C,L) [153], as well as taxonomic revisions for *Encyclia* Hook. (Figure 11I) [154], *Orleanesia* Barb. Rodr. [155], and *Pseudolaelia* [156]. The Brazilian species of *Prosthechea* were also revised in an unpublished thesis [157]. Additionally, an infrageneric classification for *Cattleya* has been proposed, which includes several infrageneric taxa endemic to Brazil [158].

Pleurothallidinae is the richest subtribe in Brazil, and it is also endemic to the Neotropics [10]. In the last decades, it has been the focus of several phylogenetic studies that have revised the circumscriptions of many genera. Currently, the subtribe encompasses 43 genera [16,159], 25 of which are recorded in Brazil. The endemicity is higher in *Pabstiella* (91% of total species), *Octomeria* (75%) (Figure 7M), and *Madisonia* Luer (67%), while the richest genera are *Acianthera* (143 spp.) (Figure 7F and Figure 9B), *Pabstiella* (132 spp.), *Anathallis* (96 spp.), and *Octomeria* (95 spp.). The subtribe presents greater diversity in the Atlantic Forest (488 spp.), followed by the Amazon Forest (113 spp.). Several molecular phylogenetic studies have been carried out on genera that are more prevalent in Brazil, such as *Acianthera* p.p. [160,161], *Pabstiella* [162], and *Madisonia* [16]. Additionally, there are taxonomic treatments for *Acianthera* p.p. [163] and *Dryadella* Luer [164], as well as the description of several new species and local floras, and papers on nomenclature notes (Supplementary File S1). A large number of species complexes make the taxonomy of some groups quite difficult, which indicates that they could benefit from multidisciplinary multipopulational studies, but these are sparse [165]. One example is the rupicolous *Acianthera teres* Lindl. complex, whose revision was based on studies of population genetics, morphometrics, floral and reproductive biology, biochemistry, and phylogeny [166,167,168,169,170,171].

Future challenges: Due to their hyperdiverse taxa, the main gaps for Laeliinae and Pleurothallidinae are related to taxonomic treatments. Within Laeliinae, some smaller genera such as *Brassavola* (10 spp.), *Constantia* (six spp.), and *Leptotes* (seven spp.), as well as those with limited diversity in Brazil such as *Scaphyglottis* Poepp. & Endl. (14 spp.), remain taxonomically challenging and require comprehensive systematic revision. Conversely, for the larger genera *Epidendrum* and *Cattleya*, a more effective strategy would involve smaller, focused taxonomic treatments at the infra-generic level. Such treatments are available for *Cattleya* but not for *Epidendrum* which still lacks a comprehensive broader phylogeny [87,90,172,173,174,175]. In Pleurothallidinae, the large genera *Acianthera*, *Anathallis*, and *Octomeria* also need molecular phylogenetic studies encompassing more comprehensive samples. Although *Acianthera* and *Pabstiella* have infra-generic classifications, only a small section, *Acianthera sect. Pleurobotryae*, was revised [163]. *Octomeria* was partially revised for the flat-leaved taxa in an unpublished thesis [176] Additionally, other smaller genera that deserve a taxonomic revision are *Barbosella* Schltr. (9 spp.), *Masdevallia* Ruiz & Pav. (14 spp.), *Myoxanthus* Poepp. & Endl. (8 spp.), *Pleurothallis* R. Br. (16 spp.), *Stelis* Sw. (36 spp.), and *Trichosalpinx* Luer (12 spp.). Furthermore, *Acianthera*, *Anathallis*, *Octomeria*, and *Pabstiella* contain numerous species complexes that also need to be reassessed to evaluate their species limits accurately. To better delimitate species and to study species complexes in these relatively young, hyperdiverse taxa, new molecular markers that detect more genetic variation are needed. Therefore, next-generation sequencing (NGS) approaches are desirable, as they yield great volumes of data from several genes [177] to complete genomes [178,179]. In Pleurothallidinae, NGS has been used to study a species complex in *Lepanthes* Sw. [180], and new molecular markers were proposed from mutational hotspots in the plastome sequences of some Brazilian species from different genera [181].

### 4.5. Cymbidieae

Cymbidieae is divided into 10 subtribes [13], 9 of which are represented in Brazil: Catasetinae (186 spp.), Coeliopsidinae (4 spp.), Cyrtopodiinae (37 spp.), Eriopsidinae (2 spp.), Eulophiinae (3 spp.), Maxillariinae (159 spp.), Oncidiinae (283 spp.), Stanhopeinae (73 spp.), and Zygopetalinae (71 spp.). Only Eulophiinae is not restricted to the Neotropics. Cymbidieae is the second most diverse tribe in Brazil, encompassing 841 spp., of which more than a half are endemic to the country (438 spp.). Eulophiinae includes only naturalized species of *Eulophia* R. Br. (including *Oeceoclades* Lindl.) and *Orthochilus* Hochst. ex A.Rich. The two species belonging to Eriopsidinae are widespread in northern South America, but restricted to the eastern slopes of the Andes [182]. Coeliopsidinae includes four species of *Peristeria* Hook, three of which are exclusive to the Amazon Forest, with one species being endemic to Brazil, and reaching the Atlantic Forest in Bahia. Cyrtopodiinae is represented by a single genus, *Cyrtopodium* (37 spp.) (Figure 8B and Figure 11B), which has its center of diversity in Cerrado, and had its nomenclature reviewed by Romero-Gonzalez et al. [107].

Stanhopeinae is composed of 20 genera [13], with 12 of these recorded to Brazil. Among them, two genera are endemic to the country, *Cirrhaea* Lindl. (seven spp.) and *Archivea* Christenson & Jenny (one sp.); the latter is known solely from an illustration [183] and probably has gone extinct. The most diverse genera are *Coryanthes* Hook. (24 spp.) (Figure 6B), *Gongora* Ruiz & Pav. (16 spp.), and *Stanhopea* J.Frost ex Hook. (14 spp.). Stanhopeinae is one of the largest orchid subtribes in Brazil which has been relatively less studied. There are some target studies available, including local floras, expansions of geographic distributions, and the description of new species (Supplementary File S1). The richest domain for Stanhopeinae is the Amazon Forest (37 spp.) followed by the Atlantic Forest (23 spp.), whereas others are significantly less diverse.

Catasetinae is organized in eight genera [13], seven of which are present in Brazil. *Grobya* Lindl. (five spp.) is endemic to the country and sister to *Cyanaeorchis* Barb. Rodr. (three spp.) (Figure 6C) [184]. *Grobya* was revised by Barros and Lourenço (2004) [185], and, among the other genera, *Galeandra* Lindl. (14 spp.) (Figure 6E and Figure 10C) was revised in an unpublished thesis [186] and has also been subject of local treatments [187,188,189]. *Mormodes* Lindl. (30 spp.) (Figure 6K) is noteworthy, as 34% of the genus occur in Brazil. However, besides the proposals of new species, there is no comprehensive taxonomic treatment for *Mormodes*, but there are molecular phylogenies published [190,191]. The largest genus in this subtribe is *Catasetum* (122 spp., including nothotaxa) (Figure 6H, Figure 7N, Figure 8C, and Figure 11D): notably, 76% of its Brazilian species are endemic to the country, and, in general, 61% of the genus diversity inhabits Brazilian forests. Due to the recent origin and high occurrence of natural hybridization, previous attempts to develop a well-resolved phylogeny based in Sanger sequences for the genus have not been successful [192]; however, efforts using complete plastomes are currently underway. While Catasetinae is the most diverse subtribe in the Amazon, it is not remarkable in the other domains. Undoubtedly, the diversity of Stanhopeinae and Catasetinae is overestimated by taxonomic inflation, especially by the high number of species published in the last decades without care on checking previously published species, their synonyms, and other names that occur in neighboring countries.

Zygopetalinae is organized into 36 genera [13], of which 21 are recorded in Brazil, and 1, *Paradisanthus* Rchb.f. (one spp.), is endemic to the country. This subtribe is among the larger orchid subtribes that have been studied less extensively in Brazil, and a challenging condition is the fact that many of its genera are not monophyletic [193] (unpublished thesis). Unfortunately, the necessary taxonomic changes have not yet been implemented. The most diverse genus is *Dichaea* Lindl. (25 spp.), with most of its taxonomic diversity concentrated in the Amazon (18 spp.). Studies in *Dichaea* from Brazil primarily rely on descriptions of new taxa (Supplementary File S1). Except for the non-monophyletic genus *Koellensteinia* Rchb.f. (nine spp.), which was revised by Meneguzzo et al. [194], all the other genera are represented by one to four species. The exact number of species in *Promenaea* Rchbf., *Zygopetalum* Hook. (Figure 7L), and *Paradisanthus* remains a topic of debate [193,195,196].

The generic circumscriptions in Maxillariinae have shifted from a splitter approach [11,197] to a much broader lumper approach [17,198]. Currently, the subtribe consists of seven genera, five of which can be found in Brazil. The richest genera in species are *Maxillaria* (116 spp.) (Figure 6N, Figure 7G, and Figure 8H) and *Bifrenaria* s.l. (33 spp.), and the latter has an exceptionally high endemism rate (72%). A taxonomic revision of the Brazilian *Maxillaria* sect. *Maxillaria* is underway; however, smaller sections have already been revised [199], and part of *Bifrenaria* Lindl. was revised by Koehler & Amaral [200]. The genus *Xylobium* Lindl. (four spp.) was revised by Ormerod [201]. Molecular phylogenies are available for *Maxillaria* sect. *Urceolatae* Christenson [202] and *Bifrenaria* s.s. [203], both including many Brazilian species. The subtribe is more representative in the Atlantic Forest (84 spp.), followed by the Amazon Forest (65 spp.).

Oncidiinae is currently organized into 63 genera [13,18], with 38 of these found in Brazil. Among these, six genera are endemic to the country: *Chytroglossa* Rchb.f. (three spp.), *Platyrhiza* Barb.Rodr. (one spp.), *Psychopsiella* Luckel & Braem (one spp.), *Rauhiella* Pabst & Braga (three spp.), *Schunkea* Senghas (one spp.), and *Thysanoglossa* Porto & Brade (three spp.). It is the third largest subtribe in Brazil after Pleurothallidinae and Laeliinae. The molecular phylogenetic study carried out by Chase et al. [204] lumped several smaller genera that were traditionally applied to Brazilian species at the time into *Gomesa* (71 spp.), making it the largest genus of the subtribe in Brazil. Other large genera in Brazil are *Notylia* Lindl. (30 spp.), *Rodriguezia* Ruiz & Pav. (24 spp.), *Trichocentrum* s.l. Poepp. & Endl. (20 spp.) (Figure 7J, Figure 11C,E, and *Zygostates* Lindl. (19 spp.). The number of species of *Notylia* is certainly overestimated; a taxonomic revision is available and the authors discuss it [205]. Furthermore, no molecular phylogeny is available yet. *Rodriguezia* has never been the subject of a proper revision, and the list for Brazil probably includes several species that do not occur in the country. A more complete phylogeny is also needed [122]. Most Brazilian species of *Trichocentrum* have wide distributions, with only two being endemic to the country. Although broader revisions of parts of the genus have been conducted [206], some species complexes in Brazil require further investigation [207]. *Zygostates* was studied by Royer et al. [208,209], but a complete revision is still needed. Smidt et al. [82] provided a molecular phylogeny of the *Ornithocephalus* clade. The subtribe is more representative in the Atlantic Forest (155 spp.), followed by the Amazon Forest (84 spp.). The difference in species distribution is primarily influenced by *Gomesa*, with 56 species occurring in the Atlantic Forest and just 2 species found in the Amazon.

Future challenges: Both taxonomic revisions and phylogenetic studies are essential for the understanding of Brazilian Cymbidieae. Some genera, such as *Quekettia* Lindl., have never been properly sequenced. Additionally, the relationships among several genera within the twig epiphyte clade of Oncidiinae remain unknown [122]. It is also necessary to re-evaluate the circumscriptions of certain genera in Zygopetalinae, since the phylogenetic data on *Aganisia*, *Koellensteinia*, *Zygosepalum*, and *Zygopetalum* indicates they are not monophyletic [193] (unpublished thesis). The hybrid identity of some taxa in *Catasetum* presents a significant shortcoming, as many (or most) accepted species may actually be natural hybrids, and the total number of taxa in Brazil can exceed 105 species, and 44 nothospecies [210,211]. Therefore, a new taxonomic revision is necessary, as the only one existing for the genus [212] is outdated due to the new circumscriptions and descriptions of new species and nothospecies which directly impacts the Brazilian listing. Taxonomic revisions for Brazilian species of *Catasetum*, *Dichaea*, *Gomesa*, *Gongora*, *Mormodes*, *Rodriguezia*, and *Stanhopea* are urgently needed. In some cases, such as in *Dichaea* or *Gomesa*, new species are expected, whereas *Gongora*, *Mormodes*, *Rodriguezia*, and *Stanhopea* are likely to have their diversity inflated.

### 4.6. Other Epidendroideae

Within the remaining lineages of Epidendroideae, taxonomic diversity is distributed across tribe Vandeae and other 13 smaller tribes [13], 8 of which are found in Brazil. Within Vandeae, there are four subtribes [13], and two of these are present in Brazil, Polystachyinae (8 spp.) and Angraecinae (40 spp.). The first is represented solely by the pantropical genus *Polystachya* Hook., while Angraecinae is represented by the native *Campylocentrum* Benth. (39 spp.) (Figure 7H) and the naturalized *Calyptrochillum christyanum* (Rchb.f.) Summerh. The exact number of species in *Polystachya* is under debate, and even the existing revisions of the genus are not congruent [213,214]. *Campylocentrum* is endemic to the Neotropics and was revised by Pessoa & Alves [215,216,217,218], which also provided a phylogeny and a historical biogeography study for the genus [219]. Brazil is the center of diversity of the genus, hosting 53% of its species. Of these, 33% are endemic, especially those from the Atlantic Forest.

Malaxideae (94 spp.) comprises two subtribes, Dendrobiinae (81 spp.) and Malaxidinae (13 spp.) [13], both present in Brazil. Dendrobiinae is represented by *Bulbophyllum* (53 spp.) (Figure 7C and Figure 9D,M), while Malaxidinae includes *Liparis* Rich. (3 spp.) and *Malaxis* Sol. ex Sw. (10 spp.) (Figure 7O), with both genera exhibiting pantropical distribution. *Bulbophyllum* was revised by Smidt [220] (unpublished thesis) and *Malaxis* by Santos [221] (unpublished thesis): most Brazilian species of these genera are endemic to the country (77% and 70%, respectively) especially from the Cerrado and Atlantic Forest. Sobralieae (33 spp.) is endemic to the Neotropics, and it is represented in Brazil by *Sobralia* Ruiz & Pav. (22 spp.) (Figure 6F) and *Elleanthus* C.Presl. (11 spp.), mainly in the Amazon Forest, although also present in other domains. Both are relatively poorly represented in the country, but much richer in the Andes [10].

Few species of the tribes Gastrodieae (three spp.), Neottieae (eight spp.), Triphoreae (nine spp.), Tropidieae (two spp.), Wullschlaegelieae (two spp.), and Xerorchideae (two spp.) are recorded to Brazil. Except for *Corymborkis* Thouars (one spp.) and *Tropidia* Lindl. (one spp.), the other genera are endemic to the American continent. Among them, we highlight *Palmorchis* Barb.Rodr. (eight species) as the richest genus. It occurs only in South America, Panamá and Costa Rica [10], and the Brazilian species are restricted to the Amazon Forest, of which four are endemic to the country. The two species of *Wullschlaegelia* (Figure 6I) are quite similar, and, while there are a few morphological features to distinguish them [222], further clarification is still needed on this matter.

Future challenges: Although well-studied, some species complexes in *Campylocentrum* and *Bulbophyllum* require further studies, which could benefit from a multidisciplinary approach [99,223]. The Brazilian species of *Elleanthus* and *Sobralia* are currently being studied. *Palmorchis* deserves a proper taxonomic treatment; furthermore, only five species of *Palmorchis* have been sequenced so far, approximately 13% of the genus, among which one is recorded from Brazil. A more complete phylogeny for the genus is necessary, including samples of Brazilian species.

## 5. Structural, Genetic, and Ecological Characterization

Brazil also has a strong research community in disciplines beyond Systematics. In the last decades, the advances in Cytogenetics and Anatomy, for instance, have been crucial for in-depth discussions on synapomorphies in unexpected clades revealed by molecular phylogenies, which were previously difficult to characterize based solely on the external morphology [7]. The development of new molecular techniques and approaches has also improved population genetics inferences and provided a clearer view of evolutionary processes [224], species delimitation [90,207], and conservation [225]. Furthermore, the increasing number of phylogenetic studies has allowed the investigation of biogeographic patterns [82,99,162,219] that help shed light on the history of the Brazilian biota. Several studies on reproductive biology provide important evidence for evolutionary inference and conservation. Below, the most important findings in recent decades in these areas are summarized, highlighting the main shortfalls.

### 5.1. Anatomy

Advanced imaging techniques have been crucial for describing and understanding the strategies developed by epiphytic orchids to establish themselves in nutrient-poor environments, such as tree canopies. In their roots, epiphytic orchids exhibit a highly specialized tissue for water absorption and retention [226,227]. Anatomical studies also provide insights into the development of vegetative and reproductive organs, as well as processes such as pollination, self-incompatibility or self-pollination, seed germination, protocorm formation, and plant acclimatization. They also shed light on the interactions between orchids and mycorrhizal fungi, and the structure and function of various secretory tissues, and support taxonomic research. In this context, research on Brazilian orchid species has increased over the past decade, revealing a rich diversity of morphological strategies associated with different life forms, including terrestrial, epiphytic, rupicolous, and semi-epiphytic habits. Additionally, many of these species are endemic and suffer influence from distinct environmental conditions and factors found across Brazil’s diverse biomes.

Orchids produce a large number of small seeds, which lack endosperm with nutrient reserves and the ability to directly utilize the substrate in nature, thus requiring mycorrhizal associations to germinate [228]. The embryo in the mature seed is in the globular stage [229], and the first structure to emerge during orchid seed germination is called the protocorm [230]. Studies on Brazilian orchid species used anatomy to investigate the germination and development of the protocorm [231,232,233,234], as well as cloning processes [235,236,237] and the cryopreservation and acclimatization of seedlings [238].

Mycoheterotrophic plants are achlorophyllous throughout their entire life cycle and obtain carbon exclusively through interactions with fungi [239,240]. The mycoheterotrophic genus *Pogoniopsis* (Triphorinae) has been used as a model plant in various studies, focusing on the morpho-anatomy, phylogeny, and genetic structure and the development of seeds and fruits [241,242,243,244]. These studies showed that *P. schenckii* Cogn. is autogamous and has low genetic variation. Furthermore, the study of reproductive organs revealed a novel interaction for mycoheterotrophic plants: the presence of fungal hyphae during the maturation of the indehiscent fruit, allowing the fungus to approach the seed even before the dispersal process, which occurs simply by the fruit falling to the ground. These fungi were isolated and used for the symbiotic germination of seeds [245]. They were capable of breaking the seed coat during in vitro symbiotic germination; however, only *Clonostachys* sp. stimulated the initial development of the protocorm. Studies with this species also led to the publication of two methodological articles—one on methods for isolating, characterizing, and identifying mycorrhizal fungi [246], and another on light, electron, and confocal microscopy techniques for studying seed and tissue development during symbiotic germination [247].

Orchid root tissue also serves as a framework for a diverse microbial community. Multilocus metabarcoding approaches have expanded our knowledge about this microbiota [248,249]. Recent studies revealed the presence of decomposer microeukaryotes from the Class Mixogastria, ciliate protists, and even strictly aquatic groups such as Marine Stramenopiles (MAST) [250,251,252]. Other notable members include diazotrophic organisms, such as cyanobacteria of the genus Nostoc and bacteria of the genera *Burkholderia* and *Bradyrhizobium* [252,253,254,255,256], as well as multicellular organisms like algae [257] and nematodes [252]. This microbiota likely contributes to plant nutrition through the decomposition of organic matter accumulated in the velamen and via biological nitrogen fixation by certain taxa.

The developmental analysis of floral organs, fruits, and seeds is fundamental for the accurate morphological interpretation of these structures within Orchidaceae and for elucidating their evolutionary patterns. Over recent decades, research has emphasized the organization and development of the ovary and fruit in orchids, including their dehiscence mechanisms [258,259,260]. They contribute to the understanding of the characters present in the early lineages of orchids [260], and also explores the organization of the carpels that result in fruits with two valves, as well as how the characters of the pericarp may influence the dispersal process of their diaspores. Ovule formation and the structural divergences observed among subfamilies are comprehensively addressed by Mayer et al. [261]. The timing of double fertilization and seed development is species-specific [258,259], and seeds may either have a developed testa or remain ategmatic [242,243]. Anatomical studies are critical for characterizing the stigma and transmitting tissue [262], and also support the investigation of reproductive phenomena such as apomixis [263] and pollination mechanisms [264,265,266,267]. The ontogeny and structural features of pollinia and the caudicle have been examined in various *Epidendrum* (Laeliinae) species by Alves et al. [244], who reported no significant anatomical differences among species. These findings suggest that such characters are evolutionarily conserved and do not correlate with the diverse reproductive strategies observed in the genus.

Secretory structures and the type of exudate produced in floral organs may vary depending on the type of pollinator being attracted. For instance, in a study of seven Brazilian species of Pleurothallidinae, nectaries and osmophores were analyzed, and the authors observed that both the position and presence of these secretory structures differed between myophilous and sapromyophilous species [268].

Extrafloral nectaries, as well as those located within the flower, may also serve to attract ants, which, in turn, defend the plant against herbivore attacks, as observed in *Coryanthes* (Stanhopeinae) [269]. The study of floral and extrafloral secretory structures in *Coryanthes macrantha* (Hook.) Hook. revealed the presence of epidermal osmophores with unicellular papillae, which were foraged by male *Eulaema* bees, along with floral nectaries located on the sepals and extrafloral nectaries on the bracts [270]. The nectaries on the bracts and sepals were visited by *Azteca* ants during both pre-anthesis and post-anthesis stages. Another secretory structure found in orchids is the colleter, which produces mucilage or lipophilic substances that protect developing buds. Colleters composed of trichomes have been identified in both vegetative organs [271] and reproductive structures [262,271,272] of different orchid species.

The anatomical structure of both vegetative and reproductive organs can provide key diagnostic characters for phylogenetic inference. Bonfante et al. [273] investigated the vegetative morpho-anatomical traits of roots, stems, and leaves and analyzed their evolutionary patterns within a phylogenetic framework for the genus *Pabstiella* (Pleurothallidinae). Based on the anatomical study of vegetative organs, the authors identified several characters with phylogenetic potential, highlighting their diagnostic value at both generic and infrageneric levels within the genus. They also emphasized the importance of integrating morphological and molecular data in phylogenetic studies. The anatomical analysis of reproductive organs is also a valuable tool, especially in taxa with minute floral structures, such as *Microchilus* (Goodyerinae). Pieczak et al. [274] analyzed the micromorphology and ultrastructure of spur secretory structures in species of this genus. They identified various types of glandular and non-glandular trichomes, suggesting that this structural diversity may contribute to understanding speciation processes in the group, possibly through pollinator shifts or behavioral changes. Anatomical studies have also been carried out on species with economic or medicinal potential, such as *Brachystele guayanensis* (Lindl.) Schltr. (Spiranthinae) [275].

Moreira et al. [276] studied 38 species of Orchidaceae from the subtropical Brazilian Atlantic Forest in search of anatomical features that could group species into functional categories. In another study, the anatomical structure of leaves and roots of epiphytic species occurring in a Guarani Indigenous Territory was analyzed to identify traits associated with the epiphytic habit [277]. Lima et al. [278] investigated the roots of *Vanilla phaeantha* Rchb.f. (Vanilleae), which may grow attached to the phorophyte, anchored in the soil, or hanging freely. The authors observed that the root habit can influence the arrangement of hemicelluloses and proteins with pectins in the cell walls, a factor that may be associated with increased wall rigidity. Some anatomical studies associated with ecophysiological studies help to understand phenotypic plasticity in vegetative organs (a characteristic frequently associated with taxonomic problems) in species with a high ecological range of occurrence, such as species that occur both as rupicolous on rocky outcrops and as epiphytes in forests associated with such environments [279,280].

Future challenges: Given the vast orchid species diversity in Brazil, and the complex network of interactions with mycorrhizal fungi and pollinators, there is a huge field that remains to be investigated and understood. Anatomical studies serve as a powerful tool for addressing various research questions, such as the development of vegetative organs, the identification and interpretation of self-pollination and apomixis, and the observation of endophytic and mycorrhizal fungi in root tissues, protocorms, and seedlings. Across all these areas, studies involving Brazilian species remain limited, representing a vast and largely unexplored field for future research.

### 5.2. Cytogenetics

Considering the 2515 native orchid species recorded in Brazil, chromosome numbers are currently known for only approximately 12% of them (361 species; Supplementary File S2). These cytogenetically studied species span ca. 80 genera across 4 subfamilies, with data accumulated over the past 65 years. From this number, 332 species from 66 genera have been cytogenetically analyzed using material collected within the country by Brazilian research groups (Supplementary File S2), underscoring the central role of national scientists in advancing cytogenetic knowledge of the Neotropical orchid flora.

The first cytogenetic study on Brazilian orchids was conducted in 1957, as part of Almiro Blumenschein’s PhD thesis at ESALQ. His work was later published, presenting chromosome numbers based on meiotic bivalent counts [281,282,283]. The next major phase, from 1988 to 2010, encompassed analyses carried out by Leonardo Felix under the supervision of Marcelo Guerra (UFPE). These studies used Giemsa staining to determine chromosome numbers for approximately 150 species across 45 genera [284,285,286,287,288], but some studies employed additional techniques as fluorescent chromosome banding using chromomycin A3 (CMA) and 4′,6-diamidino-2-phenylindole (DAPI). These fluorochromes reveal heterochromatin blocks, allowing their classification as GC-rich (CMA^+^) or AT-rich (DAPI^+^), respectively [202,289]. Subsequent studies also employed fluorescent in situ hybridization (FISH), which enables the direct localization of labeled probes on chromosomes. This technique has been widely used to investigate chromosome variation through the mapping of 5S and 35S ribosomal DNA (rDNA) sites, particularly in the subtribes Maxillariinae [290,291,292,293], Laeliinae [102,294,295,296], Pleurothallidinae [297], and Sobralieae [298].

Hybrid formation has also been explored using cytogenetic approaches, such as genomic in situ hybridization (GISH) or chromosome banding, especially in the genus *Epidendrum* [291,295,299,300]. Genome size estimation has been recently investigated on orchids, to understand the role of the genome size variation on orchid ecology [102,296,301] or in association with hybridization evaluation [300].

**Figure 12:**
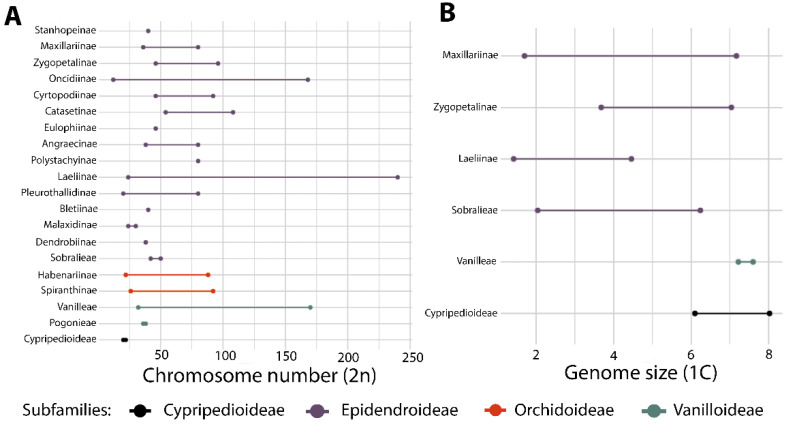
Variation in chromosome number (**A**) and genome size (**B**) among Brazilian orchid species.

Chromosome number among Brazilian species varies 20-fold (Figure 12A and Supplementary File S2), mirroring the diversity observed across the entire family from 2*n* = 12 in *Erycina pusilla* (Oncidiinae) to 2*n* = 240 in *Epidendrum cinnabarinum* Salzm. ex Lindl. (Laeliinae). That variation is among the highest reported for angiosperms, highlighting the significant role of both polyploidy and dysploidy as key drivers of chromosomal evolution within the family. Even occurring at low frequency, polyploidy is a crucial event in orchid evolution, shaping environmental niches and broadening the range of ecological habitats that can be occupied. In contrast, genome size appears to be associated with epiphytism, a key innovation that has driven the diversification of Neotropical orchids [293,301]. Genome size also shows considerable variation, ranging nearly seven-fold (likely underestimated as a result of the relatively low number of Brazilian species studied to date), from 1C = 1.18 pg in *Acianthera sudae* J. Ponert, Chumová, Mandáková & P. Trávn. (Pleurothallidinae) to 1C = 8.0 pg in *Phragmipedium lindleyanum* (M.R. Schomb. ex Lindl.) Rolfe (Cypripedioideae) (Figure 12B). Among the four subfamilies occurring in Brazil, the availability of cytogenetic data varies greatly. Cypripedioideae is represented by only three species with known chromosome numbers, whereas Epidendroideae, the most diverse subfamily in the country, has data for 153 species, corresponding to only 7.42% of its Brazilian representatives.

Chromosome number data for Cypripedioideae are available for three *Phragmipedium* species ranging from 2*n* = 20 to 2*n* = 22 (Figure 12A and Supplementary File S2). The data for Vanilloideae cover only 16.67% of the Brazilian species. *Cleistes* (Pogonieae) has two species analyzed, with chromosome numbers of 2*n* = 36 and 2*n* = 38. In Vanilleae, a single species of *Epistephium* (Vanilleae) and six of *Vanilla* were analyzed. Despite the limited data, this tribe exhibits remarkable variability in chromosome number, ranging from 2*n* = 32 in all sampled *Vanilla* species to 2*n* = 170 in *Epistephium williamsii* Hook.f. Notably, *Vanilla* species, despite their relatively low chromosome numbers, appear to have large genome sizes (1C = 7.0–7.595 pg). Analyses for representatives of Orchidoideae are more numerous, since 68 species (17.71%) from 17 genera native to Brazil have been cytogenetically analyzed. Chromosome numbers in this subfamily range from 2*n* = 22 (observed in *Habenaria aranifera* Lindl. and *H. heptadactyla* Rchb.f., Habenariinae) to 2*n* = 92, in *Sarcoglottis biflora* (Vell.) Schltr. (Spiranthinae). To date, no genome size estimation is available to Brazilian Orchidoideae species.

Epidendroideae, as the largest and most diverse subfamily, is also the most frequently analyzed. However, cytogenetic data are available for only 7.42% of its Brazilian species (153 out of 2063). The range of chromosome numbers within this subfamily mirrors that observed for the entire family. The two most represented tribes in the cytogenetic survey are Cymbidieae and Epidendreae. In Cymbidieae, 122 species have been studied, with chromosome numbers ranging from 2*n* = 12 in *Erycina pusilla* (Oncidiinae) to 2*n* = 108 in *Catasetum discolor* (Lindl.) Lindl. and *C. fimbriatum* (C. Morren) Lindl. (Catasetinae). Maxillariinae is the most sampled subtribe within Cymbidieae, with data for 76 species. Most taxa in this group exhibit 2*n* = 40, though dysploid series and rare polyploid populations have been observed, as in *Bifrenaria tyrianthina* (Lodd. ex Loudon) Rchb.f. (2*n* = 38 and 76). Despite this apparent conservatism in chromosome number, genome size within Maxillariinae varies considerably, from 1C = 1.85 to 5.69 pg. Within Epidendreae, 152 species have been analyzed, with *Epidendrum* L. (Laeliinae) as the most studied genus with chromosome numbers ranging from 2*n* = 24 in *Epidendrum fulgens* Brongn. to 2*n* = 240 in *E. cinnabarinum*, and genome sizes from 1C = 1.45 to 4.46 pg. *Cattleya* (Laeliinae) follows, with 30 species (2*n* = 40–80), including one record of 2*n* = 30, highlighting the relevance of polyploidy in this genus.

The distribution of heterochromatin blocks, as revealed by CMA and DAPI staining, shows considerable diversity, with both AT- and GC-rich bands present in orchids. In several species, these bands may represent chromosomal hotspots for rearrangements—such as translocations, inversions, fusions, or fissions—highlighting their potential role in dysploid evolution and lineage diversification. For instance, *Maxillaria valenzuelana* (A.Rich.) Nash [published as *Heterotaxis valenzuelana* (A.Rich.) Odeja & Carnevali] exhibits a centric fusion changing chromosome number from 2*n* = 42 to 2*n* = 40 [292], and *Maxillaria ferdinandiana* Barb.Rodr. [published as *Christensonella ferdinandiana* (Barb.Rodr.) Szlach., Mytnik, Górniak & Smiszek] shows 2*n* = 36 with two DAPI^+^ bands, while closely related species possess 2*n* = 36 and 38 and a single DAPI^+^ band—suggesting chromosomal fusions or fissions in this clade [202].

Future challenges: The past 65 years of orchid cytogenetic research in Brazil have revealed remarkable chromosomal variability and opened new avenues to the integration of classical cytogenetics with emerging molecular cytogenetic and genomic tools. These approaches offer promising avenues to deepening our understanding of chromosomal evolution and structure, along with the chromosome and genome size roles on speciation, adaptation, and taxonomy.

The extensive variation in chromosome number within Orchidaceae parallels the geographic, ecological, and morphological diversity of the family, positioning orchids as a valuable model for testing classical evolutionary hypotheses, such as the association between polyploidy and ecological, morphological, and physiological traits. In addition, the high susceptibility of Orchidaceae to interspecific, and even intergeneric, hybridization adds complexity to this scenario. Numerous hybrids remain to be studied at the chromosomal level to shed light on the evolutionary history of species and their hybrids.

Further cytogenetic studies should also include high-resolution molecular approaches, such as ChIP-seq for centromere mapping, helping to elucidate recurrent centric fission and fusion, the analysis of repetitive elements via next-generation sequencing (NGS), and the mapping of sequences associated with chromosome rearrangements. These approaches are essential for clarifying the mechanisms underlying genome size and chromosome number variation, as well as taxonomic and evolutionary relationships in Brazilian orchids, especially in light of their vast geographic distribution, morphological diversity, and frequent hybridization. Finally, genome size estimation will be paramount for establishing genome sizes during the design of whole-genome-sequence projects for the crescent number of species that should have their complete genome sequenced in the next decade, the main trend in genomics.

### 5.3. Evolution

Genetic studies addressing population differentiation within species are essential for understanding how genetic incompatibilities arise and contribute to lineage diversification [302,303]. Furthermore, reproductive experiments focused on different plant populations are crucial to understanding how reproductive barriers arise within species [304] and the role of outbreeding depression in speciation [305,306], clarifying the first steps involved in lineage diversification. Despite the importance of such studies to clarify the origin and maintenance of the high levels of plant diversity observed in Brazil, few studies have used orchids as models to understand the role of abiotic and biotic variables in species evolution. Ultimately, very few explored how reproductive barriers evolve among populations, giving rise to new species. The pioneer studies with Brazilian orchid populations arose in the 1960s and 1970s, with a focus on phenotypic variation. Dr. F.G. Brieger, a German botanist–geneticist, was hired in 1935 to establish the plant genetics and breeding department of the then recently created University of São Paulo, Piracicaba (ESALQ/USP). Because he was a botanist who specialized in orchids, he then started a huge orchid collection, obtaining population samples of South American orchids in expeditions from 1950. After 10 years of cultivation to decrease environmental variation, the plants had their flowers disassembled, dried, and preserved in cards for use in morphometric studies. There are about 22,000 of these cards, and several published thesis and dissertations presented morphometric analyses which were the first attempts of species delimitation studies in Brazilian or Neotropical orchids. These included *Catasetum* (Catasetinae) [307], *Prosthechea* (Laeliinae) [308], *Miltonia* (Oncidiinae) [309], *Brassavola* (Laeliinae) [310], and *Maxillaria* (Maxillariinae) [311]. The collection was also used for other studies dealing with reproductive systems [312], and cytogenetics [281]. Most studies were never published, except by several papers with new species that were discovered along the collection process. After a gap of about 30 years, new studies on the field flourished and estimates of genetic diversity, heterozygosity, inbreeding, and isolation among natural populations in Brazil have been mostly based on genetic polymorphism revealed by codominant markers of isozymes and microsatellites, or ISSR-dominant markers (Supplementary File S1).

Studies on non-epidendroid Brazilian orchids remain scarce, with only a few exceptions such as the recent work on *Habenaria* (Habenariinae) [144,313]. However, these studies primarily focus on species delimitation rather than evolutionary processes. Among basal Epidendroideae, the sole investigation involves the mycoheterotrophic terrestrial species *Pogoniopsis schenckii* (Triphorinae), which exhibited a low genetic variation and a pronounced interpopulation structure, a pattern attributed to naturally small population sizes and reproductive constraints linked to its specialized ecology [313]. The majority of research efforts in Brazil have instead concentrated on tribes Malaxideae and Epidendreae (Supplementary File S1), as for species of *Cattleya* (Laeliinae) [314,315,316,317,318,319,320], *Pseudolaelia* (Laeliinae) [321], *Bulbophyllum* (Dendrobiinae) [99,223,322,323,324], and different genera of Pleurothallidinae [168,169,325].

The analyses carried out in Brazil have shown a consistent pattern of moderate to high genetic diversity within natural populations of rupicolous orchids (Supplementary File S1). This trend has been interpreted as the result of reproductive strategies that increase the chances of outcrossing. In species of *Acianthera* [168] and *Octomeria* (Pleurothalidinae) [325], for example, mating systems with total or partial self-incompatibility mechanisms reduce the chances of autogamy. In some *Cattleya* species, flowers mimic other melittophilous species and are pollinated by food deception, allowing them to exploit the behavior of naive pollinators and increase the chances of outcrossing [316,320,326]. For these species, an increased amplitude of gametic gene flow results in greater genetic variation and enhanced connectivity among landscape patches. Furthermore, zygotic gene flow, facilitated by the long-distance wind dispersal of seeds, is common to nearly all orchids and reinforces population cohesion.

Several studies addressing evolutionary questions in Brazilian orchids were performed in the genus *Epidendrum* (Laeliinae), which has been considered a model group for understanding plant evolution in the Neotropics [327]. The ecological and evolutionary outcomes of hybrid zones have been studied in detail in several species pairs [299,300,328,329], revealing different levels of interspecific gene exchange between co-occurring species. Karyotype differences [291] and local adaptation to different environments inferred by transcriptome data and soil and climate analyses were identified as important barriers to gene exchange in *Epidendrum*.

Most studies have used different species to test barriers to gene exchange, but a decrease in gene exchange may occur among populations from the same species or ecotypes [304]. It occurs mainly on broadly distributed species occurring in different habitats [330,331,332]. Differences in several morphological traits [332], gene expression [333], and physiological responses to stress conditions [334,335] have been recorded for different orchid populations from the same species, suggesting that habitat heterogeneity may be a driver for diversification. Indeed, multidisciplinary studies using pollinator data, niche models, genetic structure, and the analysis of functional traits have shown how ecological variables may have shaped the geographical distribution of *Epidendrum fulgens*, a broadly distributed coastal species [336,337]. Diversity in several traits is not only observed among populations, but within populations as well. Arida et al. [338] show that different color morphs in *E. fulgens* are maintained by combining factors including varying pollinator-mediated selection, assortative mating due to differential pollinator preferences, and different phenotypes’ inheritabilities.

Future challenges: The studies published so far reveal the importance of investigating broadly distributed orchids occurring in different environments, and how multidisciplinary studies may increase our power to depict complex evolutionary scenarios. Nevertheless, most studies have focused on a few species of Epidendroideae, and mostly from open areas because they were easier to collect, in rocky outcrops of open vegetation areas in either the Cerrado or Restinga, highlighting the need for additional models to further explore key questions in ecology and evolution. Studies addressing non-epidendroid species from Brazil, as well as epiphytic species of all groups, are critically urgent. Furthermore, many polyploid-rich taxonomic groups occurring in Brazil, such as *Epidendrum* (Laeliinae), *Gomesa* (Oncidiinae), *Catasetum* (Catasetinae), and *Zygopetalum* (Zygopetalinae) [89,286,339], are promising models with which to investigate the role of polyploidy in driving diversification in the Neotropics. However, examining the gene flow, drift, and selection across ploidy levels remains challenging for most of these systems due to the difficulty in determining the allelic dosage and distinguishing paralogous from homologous sequences [340,341].

Understanding the evolution of Neotropical Orchidaceae presents considerable challenges due to the occurrence of hybridization and introgression, which genetic data reveal to be more frequent, and sometimes cryptic, than previously recognized [99,224,329]. These processes may play important roles in diversification and recent radiations across multiple genera in the region [342,343]. Given the potential evolutionary significance of hybridization in shaping orchid diversity, comprehensive studies investigating this phenomenon across different Neotropical orchid lineages are needed to advance our understanding of evolutionary processes in this megadiverse region.

The paucity of empirical data on reproductive and pollination biology also represents an impediment for evolutionary inference. The lack of detailed natural history data, especially regarding breeding systems and pollination strategies, represents a critical barrier to macroevolutionary studies that aim to test the hypotheses on pollinator-driven diversification [344]. Due to the floral morphology and pollination mechanisms in Orchidaceae that are deeply intertwined [345], the absence of reliable ecological data compromises the ability to interpret phylogenetic patterns and evolutionary transitions in breeding systems, floral traits, and pollinators. Addressing these gaps is therefore fundamental not only for advancing our ecological understanding, but also for enabling more robust evolutionary and comparative analyses across different orchid lineages.

### 5.4. Biogeography

The pioneer studies on biogeographical patterns of Brazilian orchids were published in 1960 [88,346], in which Brieger proposed a route from Mexico to the Andes and from the Andes to the Atlantic Forest, based on taxonomic observations on genera of Laeliinae and Oncidiinae. Pabst and Dungs [48,49] also provided some tentative biogeographical explanations without formal analyses, but studies addressing the biogeographical patterns and spatial distribution of Brazilian orchid taxa remain relatively uncommon in the literature, with the initial investigations methodologically focused on identifying centers of endemism within the Atlantic Forest for certain genera [347,348]. The biogeography of orchids has been increasingly studied in recent years, as better-grounded and more detailed phylogenetic trees have made it possible to track patterns. Most biogeographical studies on Orchidaceae in Brazil have focused on specific genera or suprageneric groups within a phylogenetic context, seeking data on origins and models that reveal patterns and investigate species colonization and dispersal routes.

Based on recent estimates, orchids diverged at the end of the Cretaceous [119,120,349], with Australasia or Laurasia being the probable initial diversification site of the family [120,349]. Therefore, the current distribution of orchid groups occurred after the fragmentation caused by plate tectonics. This pattern seems to have little relation with the long-distance dispersal capability observed in the family, as the main clades of Orchidaceae are confined to individual continents [350].

Several studies have investigated the origin and diversification of orchids found in Brazil, highlighting the influence of historical and environmental factors on their distribution. In a study of Neotropical representatives of the Pantropical genus *Bulbophyllum* (Dendrobiinae), Smidt et al. [351] propose a single colonization event in the Neotropics from African ancestral groups to northern South America, followed by dispersal across the Andes into southeastern Brazil. The Neotropical clade consists of six monophyletic sections, divided into two clades. One clade comprises two lineages and is primarily found north of the Equator, while the second clade consists of four lineages, showing significant diversity in southeastern Brazil, with numerous Brazilian endemic species from the Cerrado and Atlantic Forest. Smidt et al. [352] also used a parsimony analysis of endemicity (PAE) for searching the most parsimonious arrangement of shared *Bulbophyllum* species among areas, aiming to reveal the biogeographical affinities in a hierarchical pattern.

Smidt et al. [82] analyzed the *Ornithocephalus* clade (Oncidiinae) and inferred that its origin occurred in the Brazilian Atlantic Forest during a period when this forest was still connected to other humid formations of the Neotropics, where the group is currently distributed. On the other hand, subtribe Goodyerinae had its center of origin estimated in the Australasian region, with subsequent dispersion into the Neotropics at a time when Neotropical forests formed a continuum between the Amazon and the Atlantic coast [15]. The genus *Campylocentrum* (Angraecinae) had its evolutionary history strongly influenced by orogenic events during the Pliocene and climatic fluctuations during the Pleistocene. Its ancestor originated in Africa and, through long-distance dispersals, first established itself in the Antilles (Central America) and later in the Brazilian Atlantic Forest [219]. Studies on the species complex related to *Epidendrum latilabrum* Lindl. (Laeliinae) revealed an intricate pattern of connections between the tropical forests east of the Andes in South America. The species from the Amazon and the Atlantic Forest are intermingled in the results, suggesting that climatic changes during the Pleistocene were one of the main driving forces behind speciation [87]. Among the Pleurothallidinae, the genus *Pabstiella* originated in the Miocene, around 7.93 Mya, but its ancestral distribution could not be determined with certainty. Both the southern Andes and the Atlantic Forest have been suggested as ancestral areas, at a time when these regions were likely connected [162]. Molecular data have recently highlighted the need to expand the concept of *Madisonia* Luer, formerly a monotypic and Amazonian genus, which now, after its expansion, comprises several species found in the Atlantic Forest [16]. The genus *Dryadella* originated in northwestern South America in approximately 6.91 Mya (late Miocene-Pliocene), and underwent subsequent dispersal events for other South American regions. Its biogeographic history supports the existence of ancient connections among South America’s tropical forests [353]. For the genus *Cycnoches* Lindl. (Catasetinae), Perez-Escobar et al. [354] inferred that the most recent common ancestor emerged in the Amazon around five million years ago and later colonized Central America through a direct migration event, followed by multiple bidirectional trans-Andean migrations between the Amazon and Central America. From the same subtribe, Catasetinae, *Galeandra* and *Catasetum* [192,355] were also studied from a biogeographical perspective. *Galeandra* is a Neotropical genus containing both epiphytic and terrestrial species. Findings suggest *Galeandra* originated in Amazonia near the end of the Miocene, with the terrestrial clade emerging in the last five million years, coinciding with the expansion of dry vegetation biomes. Habit changes influenced floral spur length and geographic range, with epiphytic species developing longer spurs and narrower ranges due to specialized pollination by long-tongued Euglossini bees. Terrestrial species exhibited variable spur lengths, wider ranges, evidence of self-pollination, and loss of pollination specialization. The study underscores how climate change shaped habit evolution and associated pollinator interactions. For *Catasetum*, the study suggests the origin likely occurred between the Late Miocene and Pliocene, with its evolution shaped by events such as hybridization or incomplete lineage sorting. Two key dispersal events were identified: diversification in Mesoamerican and Pacific areas, followed by recolonization from Amazonian ancestors, indicating the Andes were not a long-lasting barrier to dispersal. Additionally, Amazonian ancestors colonized the Atlantic Forest, supporting theories of past connections between these regions.

Future challenges: Despite recent advances, many Orchidaceae lineages lack detailed biogeographical studies, and lineages in different tribes might present a diversity of patterns. Including underexplored genera could reveal hidden patterns of diversification and dispersal. Additionally, research on Neotropical epiphytic taxa is essential to understanding historical connections between the Amazon and the Atlantic Forest and the influence of drier biomes, such as the Caatinga, Cerrado, and Chaco, on species distribution. To deepen this knowledge, expanding the sampling of endemic taxa is crucial, as well as critically reviewing the group’s taxonomy and incorporating more comprehensive genomic approaches, such as NGS sequencing. Furthermore, the impact of climate change on species distribution and the role of interactions with pollinators in orchid diversification should be investigated in greater depth.

### 5.5. Conservation

The IUCN Red List is the leading source of information on global species extinction risk, based on five quantitative criteria related to population size, geographic range, and rates of decline [356]. Despite its importance, full assessments are costly and time-consuming. Therefore, less-studied groups, like tropical plants, remain largely under-assessed [357]. In Brazil, the National Center for Plant Conservation (CNCFlora) works on assessing species extinction risk, developing action plans, and identifying priority areas for conservation, contributing to both national and international biodiversity targets. Its assessments follow the same quantitative IUCN criteria and categories. CNCFlora evaluates the Extent of Occurrence (EOO) and Area of Occupancy (AOO) using verified herbarium records and all assessments are reviewed by expert panels to ensure consistency and scientific validity, ensuring alignment with international standards [358].

**Figure 13:**
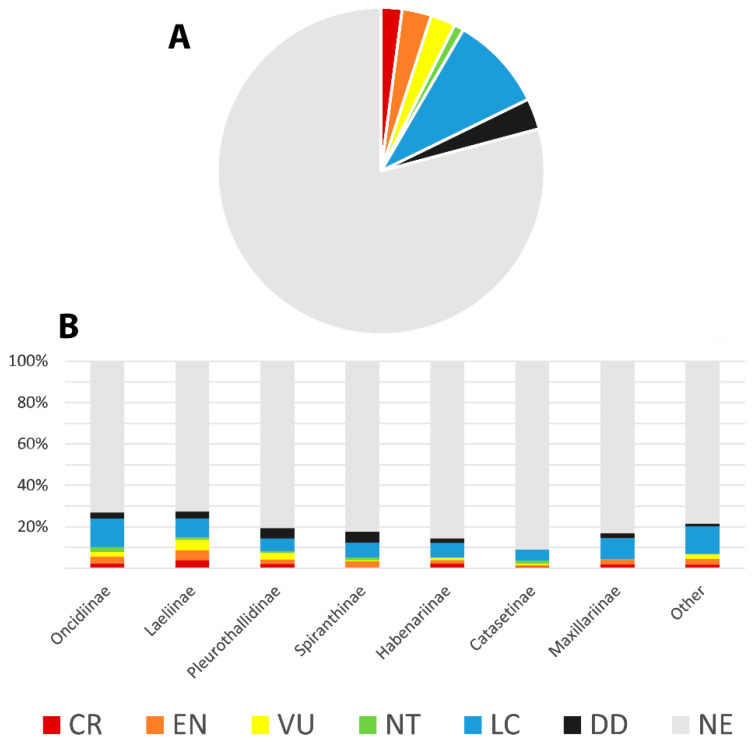
Proportion of Brazilian orchid species officially evaluated regarding their conservation statuses by CNCFlora [358]: (**A**) all Brazilian species; and (**B**) percentages by subtribe. NE: not evaluated; DD: data deficient; NT: near threatened; VU: vulnerable; EN: endangered; CR: critically endangered.

According to CNCFlora [358], only 447 orchid species (approximately 18%) have been formally assessed (excluding DD), and just 211 are currently classified within a threat category (NT, VU, EN, or CR) (Figure 13A). Thus, knowledge about the conservation status of orchids remains limited, with many species still awaiting evaluation. The understanding of extinction risk varies substantially across different Orchidaceae groups [358,359], only Oncidiinae and Laeliinae have percentages of officially assessed species above the national average in Brazil (Figure 13B).

Epidendroideae, which displays the greatest diversity and widest distribution in Brazil, contains the highest number of unofficially assessed species [208,215,216,217,218,360,361,362]. Similarly, Orchidoideae includes several frequently evaluated species, particularly those with restricted distributions or those threatened by habitat loss [123,147]. In contrast, conservation assessments for Vanilloideae and especially Cypripedioideae remain scarce. This low representation and limited data availability likely contribute to the infrequent evaluations of these subfamilies [359,363]. Moreover, conservation assessments are often overlooked by official regulatory bodies.

The Status Survey and Conservation Action Plan of the IUCN/SSC Orchid Specialist Group [364] emphasized habitat preservation, ex situ propagation, and community engagement as key conservation priorities. It highlights the urgent need to expand assessment efforts, especially given the high orchid diversity in Brazil and the increasing threats from habitat loss, fragmentation, and illegal collection [364]. The preservation of areas with high levels of endemism and species diversity represents one of the most critical strategies for the conservation of threatened species [365]. Nearly three decades after the publication of the IUCN/SSC Orchid Specialist Group [364], Amaral et al. [366] showed that, although the forest cover in the Atlantic Forest has remained relatively stable in recent decades, there has been a significant loss of mature forests. Regarding the Cerrado, Souza et al. [367] documented a substantial decline in herbaceous-shrub species richness and diversity over a 25-year period, caused by the reduced frequency of fire. The structural losses reported for the Atlantic Forest and the Cerrado—the two main biomes in terms of orchid diversity and endemism in Brazil—have direct implications for orchid conservation, particularly for epiphytic species in the Atlantic Forest that rely on large, mature trees to establish and reproduce [368]. Therefore, habitat degradation reduces the availability of suitable microhabitats, increases the number of fragmented populations, and may lead to genetic isolation, compromising the long-term viability of many species [369]. Furthermore, the fragmentation and structural impoverishment of habitats hinder seed dispersal and negatively impact essential ecological interactions for orchids, such as pollination and associations with mycorrhizal fungi [370]. Part of this degradation of mature forests occurs even within protected areas, underscoring shortcomings in their management and enforcement [366].

Additional initiatives for in situ conservation that integrate conservation education, the training of local communities to cultivate native plants propagated from seeds, the monitoring of wild populations, and the reintroduction of native plants into areas where they are threatened remain rare and scattered across the country. In the Atlantic Forest, in Serra da Cantareira, ref. [371] developed and implemented an integrated plan for orchid rescue and relocation. The plan included establishing a living collection, training environmental educators to incorporate orchids into activities with visitors, and preparing teaching materials. It emphasized the rescue of plants from fallen trees as a potential source of botanical material without the need to collect wild specimens and highlighted the orchid family as a valuable tool for educational activities. Plant reintroduction is a valuable strategy for enhancing the survival prospects of threatened species. For example, the reintroduction of *Cattleya intermedia* Graham ex Hook. (Laeliinae) in Rio Grande do Sul showed a survival rate of 60.23% after 720 days, highlighting the potential of reintroduction as a conservation tool [372].

Smidt et al. [352] studied the richness patterns, relationships of the Neotropical biomes, and complementarity analyses of *Bulbophyllum* by using a GIS framework, considering the proposed phytogeographical areas for the American Continent, showing how some tools can be useful for the fast identification of priority areas. They applied non-parametric species richness estimators to know how many species of *Bulbophyllum* (Dendrobiinae) are probable to be discovered in the Neotropical region, and which biomes are potentially richer, and a complementarity analysis in order to determine optimal locations for in situ reserves to conserve maximum species diversity.

Population genetic studies are an additional important source of information for orchid conservation, particularly for large-flowered species that are frequently targeted by illegal harvesting. Several studies have examined how habitat destruction and the removal of individuals and populations for commercial purposes affect the genetic variation within relict populations of high-value species subject to predatory collecting [163,225,315,326,339]. For example, research on Atlantic Forest orchids such as *Cattleya labiata* Lindl., *C. granulosa* Lindl., *C. coccinea* Lindl., *C. mantiqueirae* (Fowlie) Van den Berg (Laeliinae), and other congeners has revealed low genetic diversity and significant isolation among remnant populations [225,315,318,326,373]. These patterns likely reflect bottleneck events associated with recent and stochastic demographic declines, driven by reduced effective population sizes due to illegal trade and isolation in fragmented habitats. When applying these findings to the monitoring of Brazilian orchids, especially ornamental species, caution is warranted. In some cases, populations may retain moderate to high genetic variation, but this may reflect ancestral diversity rather than current resilience. A delayed genetic response to a reduced effective population size can obscure the true vulnerability of these species as their natural habitats continue to diminish and fragment.

Future challenges: The term ‘conservation’ is widely used in the scientific literature; however, most studies focus on specific aspects, such as diagnosing threats to habitats, documenting declines in wild populations, and emphasizing the need for genetic studies to enhance the effectiveness of conservation strategies. These findings highlight the importance of implementing integrated conservation actions [374].

A critical challenge in orchid conservation is the insufficient protection of natural habitats. Only 21 out of the 140 ‘Alliance for Zero Extinction’ (AZE) sites—key areas for orchid survival—in the Atlantic Forest and Cerrado, are legally protected through Conservation Units or private reserves [375]. This alarming gap underscores the urgent need to expand habitat protection to ensure the survival of wild orchid populations. In parallel, ex situ strategies must also be strengthened. Expanding germplasm banks and increasing the number of orchid species preserved in such facilities are essential for safeguarding genetic diversity. These efforts can support ecological restoration programs, species reintroductions, and even sustainable horticultural production. By providing legally sourced plants, ex situ conservation can alleviate pressure on wild populations while generating income for traditional communities [376,377].

Furthermore, conservation-oriented research must be prioritized, particularly studies on the effects of pollinator decline, habitat degradation, and climate change, which pose increasing threats to orchid populations [92,378,379,380]. These pressures are especially severe for species with narrow ecological requirements or those dependent on specific pollinators and/or mycorrhizal fungi [380]. Climate change introduces additional complexity by shifting species’ climatic niches, potentially leading to range contractions, local extinctions, and mismatches between orchids and their associated pollinators or mycorrhizal fungi [381,382,383]. In this context, ecological and reproductive biology data are crucial not only for assessing extinction risks but also for predicting species’ responses to environmental change and identifying priority areas for conservation.

Advances in orchid conservation depend on the integration of taxonomic, ecological, and genetic frameworks. Good species delimitation is essential to identify spatial prioritization. Genetic studies help to delimit cryptic species, and ecological data, such as habitat specificity, are key to understanding vulnerability and resilience to environmental change. Together, these dimensions provide more accurate assessments of priority areas for conservation.

### 5.6. Phytochemistry

Although species of Orchidaceae possess various aromatic flowers and are widely distributed in Brazil, little is known about the chemical composition of the volatile compounds present in the floral fragrance of most species. The first study in the country was conducted by Silva et al. [384], analyzing the chemical composition of the aroma of three species from the genus *Bulbophyllum* (Dendrobiinae), in which 28 compounds were identified, and, since then, other studies have also been carried out. More recent studies have predominantly focused on the Orchidaceae species occurring in the Brazilian Amazon (Supplementary Materials), and, to a lesser extent, southeast [384,385] regions. Among the 207 native or naturalized genera occurring in Brazil [13], only *Bulbophyllum*, *Catasetum* (Catasetinae), *Coryanthes* (Stanhopeinae), *Encyclia* (Laeliinae), *Gongora* (Stanhopeinae), *Notylia* (Oncidiinae), and *Stanhopea* (Stanhopeinae) have been studied, but only a few species had their compounds analyzed. The most comprehensive study was carried out with two species from the genus *Notylia* [386] and identified a total of 82 compounds that are directly related to the processes of pollinator attraction. The chemical composition of the floral fragrance in orchids pollinated by Euglossini bees, such as *Euglossa* and *Eulaema*, tends to be composed of monoterpenoids or sesquiterpenoids [387,388], which are synthesized in the osmophores found in the sepals or labellum and are key elements in the evolution of the Orchidaceae [389,390]. However, bees from the Euglossini are recognized as important pollinators of Neotropical Orchidaceae [391]; yet, their relationship with the compounds collected from the flowers of Orchidaceae has not been fully clarified., They are probably related to their reproductive behavior or the specificity of these bees to certain chemical compounds, which results in reproductive isolation in some closely related orchids [4,391]. Therefore, phytochemical studies can contribute to understanding the ecology and reproductive success of orchid species, for example, the evolutionary and reproductive processes related to variations in the chemical composition of fragrances and the relationship between orchids and their pollinators.

Borba et al. [169], in a taxonomic review using a multidisciplinary approach, characterized the leaf and flower alkaloids of 18 populations of four species of the *Acianthera teres* complex (Pleurothallidinae). The diastereoisomeric profile of the pyrrolizidine alkaloids, together with genetic and floral biology analyses, supported the distinction between two species previously considered synonymous, and also highlighted variations among disjunct conspecific populations occurring in different environments.

Future challenges: The phytochemistry of Brazilian orchids is still quite incipient, and the number of genera to be sampled is still high, as well as other species from the genera that have already been initially analyzed. The development of new techniques and equipment for extraction would be a viable alternative for the discovery of new compounds or for detecting some that are currently imperceptible to traditional methods. The improvement of gas chromatography methods is also necessary, as it has proven to be weakly effective in detecting compounds, especially in the extraction from small flowers [385].

### 5.7. Reproductive and Pollination Biology

Studies on orchid reproduction in Brazil date back to the 19th century, when pioneering works by Charles Darwin [3] and Fritz Müller [392,393] laid the groundwork for this field. In his seminal book “The Various Contrivances by which Orchids are Fertilised by Insects”, Darwin described the highly specialized pollination mechanisms of several Catasetinae (specifically, of *Catasetum*, *Mormodes*, and *Cycnoches*), which he regarded as “the most remarkable of all orchids”. He emphasized the extraordinary floral adaptations found in these genera. These early contributions, together with the ones of Stefan Vogel [387,394,395] and van der Pijl and Dodson [345], established a foundation for understanding orchid reproductive and pollination biology in the Neotropics and continue to inspire contemporary research on floral evolution and diversification [190,396,397,398,399,400,401,402]. Remarkably, studies on the reproductive system with controlled crosses and embryological studies were being pioneered in Brazilian orchids, focusing on Laeliinae, but also addressing some Cypripedioideae, *Cyrtopodium*, and *Eulophia* [312,403,404,405,406,407,408,409,410,411].

More than 150 years later after Darwin, studies on the reproductive and pollination biology of Brazilian orchids have increased considerably. Yet, our current knowledge of these systems remains surprisingly limited. Our literature review on the reproductive and pollination biology of Brazilian orchid species recovered 66 works and revealed an astonishing picture of only ca. 11% of the Brazilian species (290 spp.) which have had aspects of their reproductive and/or pollination biology investigated (Supplementary File S3). Moreover, scientific attention has been highly uneven across orchid lineages. At the subtribe level, the distribution of studies reveals a markedly unbalanced research effort (Supplementary File S3). Among the 23 subtribes that occur in Brazil, 10 have no recorded studies, representing complete knowledge gaps. There is a substantial heterogeneity in sampling coverage, with some subtribes demonstrating low species richness, such as Calypsoinae (1 spp.) and Bletiinae (2 spp.), that exhibit high proportional coverage (100% and 50%, respectively). This trend does not hold across all low-diversity groups; for example, Discyphinae and Xerorchideae remain completely neglected (Supplementary File S3).

**Figure 14:**
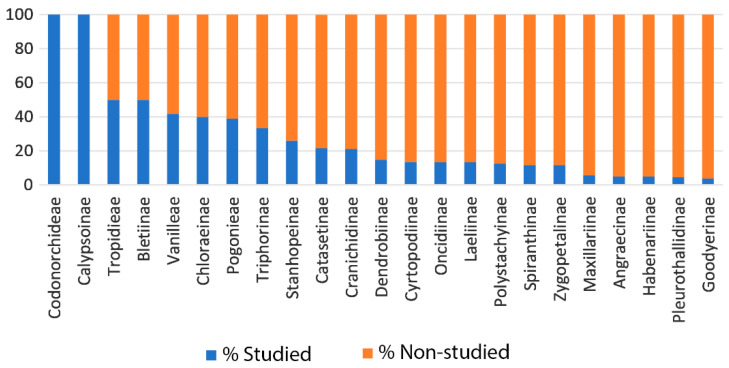
Proportion of Brazilian orchid species investigated for reproductive and/or pollination biology by taxa.

In contrast, some moderately diverse groups, such as the tribe Vanilleae (41.6%) and the subtribes Stanhopeinae (26%), Catasetinae (21.5%), and Cranichidinae (21.1%) are comparatively better studied. Interestingly, large and taxonomically important subtribes like Pleurothallidinae (629 spp.) and Laeliinae (387 spp.) have been studied in only 4.5% and 13.3% of their species, respectively (Supplementary File S3). The low percentages are clearly due to the high number of species in these groups. These patterns underscore a strong taxonomic bias in research effort and the absence of a systematic approach, with significant gaps even among the largest and most emblematic orchid groups (Figure 14).

The uneven research distribution becomes even more pronounced at the genus level. Among the 202 orchid genera occurring in Brazil, approximately 59% have not been the subject of any study on reproductive or pollination biology (Supplementary File S3). On average, only 14% (±26.4% SD) of the species within each genus have been investigated. Clearly, research has disproportionately focused on a few showy and emblematic genera, including *Stanhopea* (Stanhopeinae) (80%), *Vanilla* (Vanileae) (50%), *Catasetum* (Catasetinae) (38.4%), and *Cattleya* (Laeliinae) (22.4%). These genera are characterized by large, visually striking flowers with elaborate morphologies and, in many cases, intense floral fragrances [387,396,399,412,413]. By contrast, some of the most diverse genera in the Brazilian flora, especially the ones with inconspicuous-flowered species, remain strikingly underexplored [e.g., *Acianthera* (8.4%), *Octomeria* (7.4%), *Habenaria* (5.1%), *Anathallis* (4.2%), and *Pabstiella* (0.8%) (Supplementary File S4)].

It is important to note that addressing this bias is not merely a matter of shifting scientific interest; it is also constrained by practical limitations in the field. Many orchid species endemic to the Amazon occur in areas that are extremely difficult and costly to access [414]. In this context, citizen science initiatives [185,415,416,417,418], particularly involving experienced amateur orchid growers, may offer a valuable alternative by enabling data collection on phenology, floral visitors, and reproductive traits under cultivation or in more accessible regions, thus complementing formal fieldwork in hard-to-reach areas.

Another important finding emerging from our review is the incomplete nature of many published studies. Despite notable progress, truly integrative investigations that address both reproductive traits (e.g., breeding system and flowering phenology) and pollination biology (e.g., pollination mechanisms, floral visitors, and effective pollinators) remain exceedingly rare. Complete studies are available for only 5.7% of Brazilian orchids (134 species), representing nearly half (47.5%) of the studies in the area [396,400,401,419,420,421]. This pronounced shortfall has important implications for our understanding of orchid reproductive ecology. Many of the reproductive biology studies performed to date focus on breeding system assessments (such as tests for self-compatibility, autogamy, and fruit set under controlled pollination). Other studies often incorporate reproductive phenology, providing valuable insights into the flowering duration, synchrony, and temporal overlap with pollinator activity. However, when these phenological and breeding system data are not paired with field-based pollinator observations, they offer only a partial picture of reproductive success and evolutionary fitness under natural conditions.

Conversely, studies centered on pollinator behavior and visitation patterns frequently omit experimental confirmation of fruit production or compatibility systems [414,422]. As a result, we often cannot determine whether floral visitors are effective pollinators, nor whether plant reproductive success depends on their availability. This disconnection between ecological and experimental approaches hampers our ability to assess whether fruit production results from selfing, cross-pollination, or autonomous autogamy. It limits broader interpretations of pollination dependency, reproductive assurance, and the adaptive value of floral traits in Orchidaceae. Future studies should also address the underexplored role of cognitive ecology in Orchidaceae, particularly investigating how color and odor signals in specific genera influence pollinator behavior and reproductive strategies. Other key unresolved questions include whether the reported prevalence of self-compatibility (>80% of orchids) extends to Brazilian species, and if melittophily represents a basal trait in this family. Such insights could reveal novel adaptive mechanisms and clarify the evolution in the family.

Contrary to what was initially thought and accepted for more than a century in the basic Orchidaceae literature, the group is far from being strictly allogamous and may actually be one of the families with the greatest variation in reproductive systems among angiosperms. Autogamy, including that mediated by spontaneous self-pollination, self-incompatibility, and even agamospermy, should no longer be considered mere exceptions. Beyond simply reporting numerous new cases of all these different reproductive modes, we are beginning to realize in Orchidaceae that all of these comprise a wide range of mixed reproductive systems, which can coexist within the same or different conspecific populations [263]. In general, studies of mating systems in Brazilian orchids are not only scarce across several groups in the country, but also lacking comprehensive studies within specific groups, encompassing a large number of species, and especially associated with phylogeny and population genetics. Such studies are essential for understanding the lability and evolution of these mating systems in Orchidaceae. Borba et al. [423] addressed the determination of the reproductive system through experimental pollinations in all major lineages of Pleurothallidinae in order to determine in which group self-incompatibility has possibly appeared for the first time, and how many times it has evolved. The results indicate that self-incompatibility is a generalized widespread feature of the myophilous clade of the Pleurothallidinae and possibly evolved early in the subtribe. In a different scenario, both self-compatibility and self-incompatibility occur in the Neotropical lineage of *Bulbophyllum*, and these different reproductive modes appear to be relatively stable in different sections of the genus, which are associated with different pollination mechanisms [324,424].

Future challenges: Despite recent advances, orchid reproductive biology in Brazil remains a young and fragmented field. Addressing the uneven distribution of knowledge across subtribes and genera requires urgent, coordinated efforts. Future studies must strive for greater taxonomic and ecological representativeness, including underrepresented genera, inconspicuous small-flowered species, and lineages from neglected habitats. This imbalance is not limited to gaps in sampling, but reflects deeper structural patterns in how research attention has been historically distributed. One of the most pressing challenges is the strong bias toward taxa with showy flowers; it should be counterbalanced by focused efforts on hyperdiverse and poorly studied tiny flowered genera such as *Acianthera* (Pleurothallidinae), *Habenaria* (Habenariinae), and *Pabstiella* (Pleurothallidinae). These groups likely harbor a rich, unexplored repertoire of reproductive and pollination strategies, yet remain virtually absent from the literature. Meeting these challenges will require a multidisciplinary and inclusive approach, one that not only fills taxonomic and geographic gaps, but also situates orchid reproductive and pollination biology within broader ecological and conservation frameworks. Doing so will ensure a more complete understanding of Orchidaceae in Brazil and support efforts to safeguard this emblematic group in the face of growing environmental threats.

## 6. Conclusions

Over almost four centuries of orchid research established Brazil as one of the global centers of Orchidology. The accumulated knowledge has unveiled an exceptional diversity and high levels of endemism. We exemplified how diversified the Brazilian botanical community is by compiling the current data on classical taxonomy, molecular systematics, anatomy, cytogenetics, reproductive biology, population genetics, and conservation. Despite these advances, there are pronounced disparities among regions, taxa, and research approaches. We demonstrated that, while certain groups (e.g., Epidendreae and Cymbidieae) and areas (eg. Atlantic Forest) are relatively well-documented, vast portions of the country and certain taxa remain poorly explored. The persistence of the Linnean, Wallacean, and Darwinian shortfalls highlights the urgent need for integrative research frameworks. The progress in Brazilian orchidology depends on the strengthening of collaborative networks and interdisciplinary approaches. Future perspectives for each knowledge area were also provided, and, in addressing these challenges, Brazil can further consolidate its role as a global reference in Orchidaceae research, contributing not only to the advancement of taxonomy and systematics but also to the preservation of the most emblematic and diverse plant family.

## Figures and Tables

**Table 1:** Number of Brazilian species of Orchidaceae organized by subfamilies, tribes, and subtribes according to Chase et al. [13]. (N/A: not applicable).

Subfamilies		Tribes		Subtribes	
	Total (Endemic)		Total (Endemic)		Total (Endemic)
Cypripedioideae	11 (4)	N/A	-	N/A	-
Orchidoideae	387 (230)	Codonorchideae	1 (1)	N/A	
		Cranichideae	206 (112)	Cranichidinae	19 (13)
				Goodyerinae	27 (9)
				Spiranthinae	154 (90)
				Chloraeinae	5 (0)
				Discyphinae	1 (0)
		Orchideae	180 (117)	Habenariinae	180 (117)
Vanilloideae	54 (25)	Pogonieae	18 (11)	N/A	-
		Vanilleae	36 (14)	N/A	-
Epidendroideae	2063 (1281)	Cymbidieae	818 (440)	Catasetinae	186 (127)
				Coeliopsidinae	4 (1)
				Cyrtopodiinae	37 (19)
				Eriopsidinae	2 (0)
				Eulophiinae	3 (0)
				Maxillariinae	159 (71)
				Oncidiinae	283 (163)
				Stanhopeinae	73 (33)
				Zygopetalinae	71 (26)
		Epidendreae	1044 (748)	Bletiinae	2 (1)
				Pleurothallidinae	642 (484)
				Calypsoinae	1 (0)
				Ponerinae	2 (1)
				Laeliinae	397 (262)
		Gastrodieae	3 (2)	N/A	-
		Malaxideae	94 (49)	Dendrobiinae	81 (41)
				Malaxidinae	13 (8)
		Neottieae	8 (4)	N/A	-
		Sobralieae	33 (8)	N/A	-
		Triphoreae	9 (4)	Triphorinae	9 (4)
		Tropidieae	2 (0)	N/A	-
		Vandeae	48 (26)	Angraecinae	40 (24)
				Polystachynae	8 (2)
		Wullschlaegelieae	2 (0)	N/A	
		Xerorchideae	2 (0)	N/A	-
Total	2515 (1540)				

## Data Availability

Supporting information is included as Supplementary files.

## Supplementary Materials

The following supporting information can be downloaded at: https://www.mdpi.com/article/10.3390/plants14223520/s1, Supplementary File S1: The complete list of references we gathered; Supplementary File S2: Survey of chromosome data focused on Brazilian Orchidaceae; Supplementary File S3: Survey of studies on pollination focused on Brazilian Orchidaceae; Supplementary File S4: Checklist of Orchidaceae from Brazilian Pantanal.
