# Supplementary material for: An Overview of Orchidaceae from Brazil: Advances and Shortfalls After 400 Years of Studies
Source: Plants (Basel). 2025 Nov 18;14(22):3520. doi: 10.3390/plants14223520 (PMC12656190; doi:10.3390/plants14223520)
Supplement: Supplementary file 1 [file plants-14-03520-s001.zip › plants-3937747-supplementary/Supplementary file S1.pdf]

SYSTEMATICS, EVOLUTION AND BIOGEOGRAPHY

Abreu, N.L.; Menini–Neto, L.; Konno, T.U.P. Orchidaceae das Serras Negra e do Funil, Rio Preto, Minas Gerais, e similaridade florística entre formações campestres e florestais do Brasil. *Acta Bot. Bras.* **2011**, *25*, 58–70.

Abreu, N.L.; Menini-Neto, L. As subfamílias Vanilloideae e Orchidoideae (Orchidaceae) em um fragmento da Serra da Mantiqueira, Minas Gerais, Brasil. *Bol. Bot. Univ. São Paulo* **2010**, *28*, 15–33.

Abreu, N.L.; Alves, R.J.V.; Cardoso, S.R.S.: Bertrand, Y.J.K.; Sousa, F.; Hall, C.F.: Pfeil, B.E.; Antonelli, A. The use of chloroplast genome sequences to solve phylogenetic incongruences in *Polystachya* Hook (Orchidaceae Juss). *PeerJ* **2018**, *6*, e4916.

Afonso, E.A.L.; Koch, A.K; Costa, J.M. Flora preliminar de Orchidaceae no município de Abaetetuba, Pará, Brasil. *Biota Amaz.* **2016**, *6*, 107–118. http://dx.doi.org/10.18561/2179-5746/biotaamazonia.v6n1p107-118.

Almeida, P.R.M.; van den Berg, C.; Góes–Neto, A. Morphological and molecular characterization of species of *Tulasnella* (Homobasidiomycetes) associated with Neotropical plants of Laeliinae (Orchidaceae) occurring in Brazil. *Lankesteriana* **2007**, *7(1–2)*, 22–27.

Almeida, P.R.M.; López-Roberts, M.C.; Vigna, B.B.Z.; Souza, A.P.; van den Berg, C. Microsatellite markers for the endangered orchids *Cattleya labiata* Lindl. and *C. warneri* T. Moore (Orchidaceae). *Cons. Genet. Res.* **2013**, *5(3)*, 791–794.

Almeida, P.R.M.; van den Berg, C.; Góes-Neto, A. *Epulorhiza amonilioides* sp. nov.: a new anamorphic species of orchid mycorrhiza from Brazil. Neodiversity **2014**, *7(1)*, 1–10.

Araujo, A.M.; Farronay, F.; Perdiz, R.O.; Pessoa, E.M.; Giacomin, L. The discovery of *Scaphyglottis punctulata* (Laeliinae) in the highlands of Brazilian amazonia with a key to the species of the region. *Lankesteriana* **2022**, *22*, 123–131.

Arida, B.L.; Pinheiro, F.; Laccetti, L.; Camargo, M.G.G.; Freitas, A.V.L.; Scopece, G. The consequences of flower colour polymorphism on the reproductive success of a neotropical deceptive orchid. *Pl. Biol.* **2025**, https://doi.org/10.1111/plb.70020.

Arida, B.L.; Izquierdo, J.V.; Teixeira, M.C.; Turchetto, C.; Benitez-Vieyra, S.; Pinheiro, F. Different but not isolated: absence of reproductive barriers and strong floral divergence between ecotypes of *Epidendrum fulgens* (Orchidaceae). *Bot. J. Linn. Soc.* **2025**, *208*(3), 313–324. https://doi.org/10.1093/botlinnean/boae076.

Arida, B.L.; Scopece, G.; Machado, R.M.; Moraes, A.P.; Forni-Martins, E.; Pinheiro, F. Reproductive barriers and fertility of two Neotropical orchid species and their natural hybrid. *Evol. Ecol.* **2021**, *35*, 41–64. http://dx.doi.org/10.1007/s10682-020-10095-5.

Arrigoni-Blank, M.F., Santos, M.S., Blank, A.F., Rabbani, A.R.C., Silva-Mann, R., Santos, J.B., Costa, A.S.; Menezes, T.S.A. Analysis of genetic diversity of Laeliinae (Orchidaceae) in the State of Sergipe using ISSR markers. *Genet. Mol. Res.* **2016**, *15(2)*, 1–9. http://dx.doi.org/10.4238/gmr.15027997.

Azevedo, C.O.; van den Berg, C. A new combination in the genus *Anathallis* (Orchidaceae), and a new record for Bahia State, Brazil. *Kew Bull.* **2005**, *60(1)*, 137–138.

Azevedo, C.O.; van den Berg, C. Lectotypifications in *Prescottia. Kew Bull.* **2007**, *62*, 651–655.

Azevedo, C.O.; van den Berg, C. Análise comparativa de áreas de campo rupestre da Cadeia do Espinhaço (Bahia e Minas Gerais, Brasil) baseada em espécies de Orchidaceae. *Sitientibus Ci. Biol.* **2007**, *7*, 199–210.

Azevedo, C.O.; Smidt, E.C.; van den Berg, C. *Prescottia mucugensis*: a new species of *Prescottia* (Orchidaceae: Cranichidinae) from Bahia, Brazil. *Kew Bull.* **2010**, *65*, 263–267.

Azevedo, C.O.; van den Berg, C. A família Orchidaceae no Parque Municipal de Mucugê, Bahia, Brasil. *Hoehnea* **2007**, *34*, 1–47.

Azevedo, C.O.; Marinho, L.C. *Acianthera saurocephala* (Lodd.) Pridgeon; MW Chase (Orchidaceae: Pleurothallidinae): novo registro para o Nordeste brasileiro. *Rev. Bras. Bioci.* **2011**, *9(4)*, 554–554.

Azevedo, C.O.; Marinho, L.C. Novos registros de Orchidaceae para o Nordeste brasileiro: *Acianthera tricarinata* e *Cyclopogon variegatus*. *Sitientibus sér. Ciênc. Biol.* **2012**, *12(2)*, 339–344.

Azevedo, C.O.; Borba, E.L.; van den Berg, C. Evidence of natural hybridization and introgression in *Bulbophyllum involutum* Borba, Semir; F. Barros and *B. weddellii* (Lindl.) Rchb. f. (Orchidaceae) in the Chapada Diamantina, Brazil, by using allozyme markers. *Braz. J. Bot.* **2006,** *29,* 415–421. http://dx.doi.org/10.1590/s0100-84042006000300008

Azevedo, C.O.; van den Berg, C.; Barros, F. A revision of *Prescottia* (Orchidaceae: Orchidoideae, Cranichideae). *Phytotaxa* **2014**, *178*, 233–286. https://doi.org/10.11646/phytotaxa.178.4.1.

Azevedo, C.O.; Leoni, L.S.; van den Berg, C. Clarification on the circumscription of *Prescottia glazioviana* (Cranichidinae, Orchidaceae). *Phytotaxa* **2012**, *57*, 23–26.

Azevedo, M.T.A.; Borba, E.L.; Semir, J.; Solferini, V.N. High genetic variability in Neotropical myophilous orchids. *Bot. J. Linn. Soc.* **2007**, *153*, 33–40. https://doi.org/10.1111/j.1095-8339.2007.00589.x.

Barberena, F.F.V.A.; Gonzaga, D.R. A new species of *Epidendrum* (Epidendroideae; Orchidaceae) from the Brazilian Atlantic Forest. *Phytotaxa* **2016**, *284(3)*, 225–230.

Barberena, F.F.V.A.; Gastin, J.R.; Smidt, E.C.. Taxonomical remarks on *Promenaea microptera* (Orchidaceae: Epidendroideae): the rediscovery of a poorly known micro-endemic orchid from the Brazilian Atlantic Forest. *Phytotaxa* **2022**, *545*, 229–233.

Barberena, F.F.V.A. Revisão Taxonômica e Filogenia do Gênero *Promenaea* Lindl. (Orchidaceae). PhD dissertation, Universidade Federal do Rio de Janeiro, Rio de Janeiro, 2014.

Barbosa, A.R.; Silva-Pereira, V.; Borba, E.L. High Genetic variability in self-incompatible myophilous *Octomeria* (Orchidaceae, Pleurothallidinae) species. *Bras. J. Bot.* **2013**, *36*, 179–187. https://doi.org/10.1007/s40415-013-0027-0.

Barrera-Rojas, C.H.; van den Berg, C. A new natural hybrid of *Cattleya* (Orchidaceae: Laeliinae) from Bahia, Brazil. Phytotaxa **2025**, *689(1)*, 139–145.

Barros, F. Notas Taxonômicas para Espécies Brasileiras dos Gêneros *Epidendrum, Platystele, Pleurothallis* e *Scaphyglottis* (Orchidaceae). *Acta Bot. Bras.* **1996,** *10,* 139–151. https://doi.org/10.1590/S0102-33061996000100011.

Barros, F.; Batista, J. A.N.; Bianchetti, L.B. Epitypification and taxonomic elucidation of some Brazilian taxa of *Cyrtopodium* R.Br.(Orchidaceae). *Taxon* **2003**, *52(4)*, 841–849.

Barros, F.; Pinheiro, F. Flora de Grão-Mogol, Minas Gerais: Orchidaceae. *Bol. Bot. Univ. São Paulo*, **2004**, *22(2)*, 361–383.

Barros, F.; Barberena, F.F.V. Nomenclatural notes and new combinations on *Anathallis* and *Specklinia* (Orchidaceae). *Rodriguésia* **2010**, *61(1)*, 127–131.

Barros, F.; Hall, C.F. Novo sinônimo para o gênero *Acianthera* e novas combinações em *Pabstiella* (Pleurothallidinae: Orchidaceae). *Orquidário* **2012**, *26*, 27–32.

Barros, F.; Hall, C.F.; Paiva Neto, V.B.; Batista, J.A.N. Check-list das Orchidaceae do Estado de Mato Grosso do Sul, Brasil. *Iheringia, Sér. Bot.* **2018**, *73*, Supl., 287–296. https://doi.org/10.21826/2446-8231201873s287.

Barros, F.; Lourenço, R.A. Synopsis of the Brazilian orchid genus *Grobya*, with the description of two new species. *Bot. J. Linn. Soc.* **2004**, *145*, 119–127. https://doi.org/10.1111/j.1095-8339.2003.00277.x.

Bastos, C.A.; Meneguzzo, T.E.C.; van den Berg, C. A Taxonomic revision of the Brazilian species of *Encyclia* (Orchidaceae: Epidendroideae: Epidendreae). *Phytotaxa* **2018**, *342*, 1–84. https://doi.org/10.11646/phytotaxa.342.1.1.

Bastos, C.A.; van den Berg, C. Flora da Bahia: *Catasetum* (Orchidaceae). *Sitientibus Ciênc. Biol.* **2012**, *12(1)*, 83–89.

Bastos, C.A.; van den Berg, C.; Meneguzzo, T.E.C.. *Encyclia fimbriata* (Orchidaceae: Laeliinae), a new large-flowered species from Bahia, Brazil. *Phytotaxa* **2012**, *40(1)*, 26–30.

Bastos, C.A.; van den Berg, C. A família Orchidaceae no município de Morro do Chapéu, Bahia, Brasil. *Rodriguésia* **2012**, *63*, 883–927.

Bastos, C.A.; Meneguzzo, T.E.C.; van den Berg, C. Flora da Bahia: *Encyclia* (Orchidaceae). *Sitientibus ser. Cienc. Biol.* **2016**, *16*, 2–18.

Batista, J.A.N.; Bianchetti, L.B. Notes on *Habenaria orchiocalcar* (Orchidaceae), an overlooked species from central Brazil. *Lindleyana* **2002**, *17*, 115–121.

Batista, J.A.N.; Bianchetti, L.B. A review of *Habenaria* (Orchidaceae) in Pabst and Dungs' Orchidaceae brasilienses. *Lindleyana* **2002**, *17*, 75–84.

Batista, J.A.N.; Bianchetti, L.B. Taxonomy, distribution and new taxa from the *Habenaria crucifera* (section *Nudae*, Orchidaceae) aggregate from Brazil and the Guianas. *Brittonia* **2010**, *62*, 57–79. https://doi.org/10.1007/s12228-009-9099-3.

Batista, J.A.N., Bianchetti, L.B.; Gonçalves, E.G. An overlooked new species of *Habenaria* (Orchidaceae) from central Brazil. *Novon* **2003**, *13*, 397–402. https://doi.org/10.2307/3393369.

Batista, J.A.N.; Bianchetti, L.B. Three new taxa in *Cyrtopodium* (Orchidaceae) from central and southeastern Brazil. *Brittonia* **2004**, *56(3)*, 260–274.

Batista, J.A.N., Bianchetti, L.B., Nogueira, RE., Pellizzaro, K.F.; Ferreira, F.E. The genus *Habenaria* (Orchidaceae) in the Itacolomi state park, Minas Gerais, Brazil. *Sitientibus, sér. Ci. Biol.* **2004**, *4*, 25–36.

Batista, J.A.N.; Bianchetti, L.B. Two new taxa in *Cyrtopodium* (Orchidaceae) from southern Brazil. *Darwiniana* **2005**, *43(1/4)*, 74–83.

Batista, J.A.N.; Bianchetti, L.B. The Brazilian *Habenaria* (Orchidaceae) with hairy segments. *Sitientibus, sér. Ciênc. Biol.* **2006**, *6*, 9–23.

Batista, J.A.N., Bianchetti, L.B.; Miranda, Z.J. A revision of *Habenaria* section *Macroceratitae* (Orchidaceae) in Brazil. *Brittonia* **2006**, *58(1)*, 10–41. https://doi.org/10.1663/0007-196X(2006)58[10:AROHSM]2.0.CO;2.

Batista, J.A.N., Bianchetti, L.B.; Miranda, Z.J.G. Two new species of *Habenaria* (Orchidaceae) from the Brazilian cerrado and campo rupestre. *Kew Bull.* **2008**, *63*: 449–456. https://doi.org/10.1007/s12225-008-9054-6.

Batista, J.A.N., Mota, R.C. Abreu, N.L.; Menini-Neto, L. *Habenaria pseudoglaucophylla* (Orchidaceae), a new species from Minas Gerais, Brazil. *Novon* **2008**, *18*, 409–414. https://doi.org/10.3417/2006174.

Batista, J.A.N., Silva, J.B.F.; Bianchetti, L.B. The genus *Habenaria* (Orchidaceae) in the Brazilian Amazon. *Braz. J. Bot.* **2008**, *31*, 105–134. https://doi.org/10.1590/S0100-84042008000100011.

Batista, J.A.N., Carvalho, B.M., Ramalho, A.J.; Bianchetti, L.B. Three new species of *Habenaria* (Orchidaceae) from Serra da Canastra, Minas Gerais, Brazil. *Phytotaxa* **2010**, *13*, 27–39. https://doi.org/10.11646/phytotaxa.13.1.2.

Batista, J.A.N.; Bianchetti, L.B.; González-Tamayo, R.; Figueroa, X.M.C.; Cribb, P.J. A Synopsis of New World *Habenaria* (Orchidaceae) I. *Harv. Pap. Bot.* **2011**, *16*, 1–47. https://doi.org/10.3100/025.016.0101.

Batista, J.A.N.; Bianchetti, L.B.; González-Tamayo, R.; Figueroa, X.M.C.; Cribb, P.J. A Synopsis of New World *Habenaria* (Orchidaceae) II. *Harv. Pap. Bot.* **2011**, *16*, 233–273. https://doi.org/10.3100/0.25.016.0202.

Batista, J.A.N.; Meneguzzo, T.E.C.; Salazar, G.A.; Bianchetti, L.B.; Ramalho, A.J. Phylogenetic placement, taxonomic revision, and a new species of *Nothostele* (Orchidaceae), an enigmatic genus endemic to the cerrado of central Brazil. *Bot. J. Linn. Soc.* **2011**, *165*, 348–363. https://doi.org/10.1111/j.1095-8339.2011.01113.x

Batista, J.A.N., Menini-Neto, L.; Vale, A.A. Three new species, four new records and an updated checklist of *Habenaria* (Orchidaceae) from Rio Grande do Sul, Brazil. *Nordic J. Bot.* **2012**, *30*, 277–290. https://doi.org/10.1111/j.1756-1051.2012.01349.x.

Batista, J.A.N.; Borges, K.S.; Faria, M.W.F.; Proite, K.; Ramalho, A.J.; Salazar, G.A.; van den Berg, C. Molecular phylogenetics of the species-rich genus *Habenaria* (Orchidaceae) in the New World Based on Nuclear and Plastid DNA Sequences. *Mol. Phylogenet. Evol.* **2013**, *67*, 95–109. https://doi.org/10.1016/j.ympev.2013.01.008.

Batista, J.A.N.; Mota, A.C.M.; Proite, K.; Bianchetti, L.B.; Romero-González, G.A.; Espinoza, H.M.H.; Salazar, G.A. Molecular Phylogenetics of Neotropical *Cyanaeorchis* (Cymbideae, Epidendroideae, Orchidaceae): Geographical Rather Than Morphological Similarities Plus a New Species. *Phytotaxa* **2014**, *156*, 251–272. https://doi.org/10.11646/phytotaxa.156.5.1.

Batista, J.A.N., Vale, A.A., Carvalho, B.M., Proite, K., Ramalho, A.J., Munhoz, A.C.D., van den Berg, C.; Bianchetti, L.B. Four new species in *Habenaria* (Orchidaceae) from the Espinhaço Range, Brazil. *Syst. Bot.* **2016**, *41*, 275–292. https://doi.org/10.1600/036364416X691858.

Batista, J.A.N., Proite, K.; Bianchetti, L.B. Descriptions and phylogenetic relationships of four new species and a new name of *Habenaria* (Orchidaceae) from the cerrado and campos rupestres of Brazil. *Pl. Syst. Evol.* **2017**, *303*, 873–899. https://doi.org/10.1007/s00606-017-1415-x.

Batista, J.A.N., Reis, A.F.S., Leite Junior, J.L.; Bianchetti, L.B. Phylogeny and taxonomy of *Habenaria leprieurii*, *H. alpestris* and *H.* sect. *Microdactylae* (Orchidaceae), with descriptions of two new taxa. *Phytotaxa* **2018**, *373*, 241–271. https://doi.org/10.11646/phytotaxa.373.4.1.

Batista, J.A.N., Meyer, P.B., Cruz-Lustre, G.; Toscano de Brito, A.L.V. A remarkable new species of the *Habenaria nuda* Complex (Orchidaceae) from the Espinhaço Range of Eastern Brazil. *Syst. Bot.* **2021**, *46*, 790–798.

Batista, J.A.N., Ferreira, V.L., Alves, C.I.; Stehmann, J.R. A new species of *Habenaria* (Orchidaceae, Asparagales) and a checklist of Orchidaceae from limestone outcrops of Brazil. *Eur. J. Taxon.* **2022**, *828*, 16–44. https://doi.org/10.5852/ejt.2022.828.1855.

Batista, J.A.N.; Castro, C.; Sambin, A.; Cruz-Lustre, G.; Pansarin, E.R. Clarifying the identity of the *Cleistes rosea* complex (Orchidaceae) based on integrative taxonomy. *Syst. Biodivers.* **2023**, *21*, 2207575. https://doi.org/10.1080/14772000.2023.2207575.

Batista, J.A.N., Cruz-Lustre, G., Vale, A.A.; Bianchetti, L.B. Checklist and molecular phylogenetics reveal three taxonomic novelties in *Habenaria* (Orchidaceae, Orchidoideae) from Chapada dos Veadeiros, Goiás, Brazil. *Eur. J. Taxon.* **2023**, *891*, 51–86. https://doi.org/10.5852/ejt.2023.891.2271.

Bicalho, H.D. Contribuição à sistemática do gênero *Catasetum* L.C.Rich (Orchidaceae). PhD dissertation, Universidade de São Paulo, Piracicaba, 1960.

Bochorny, T., Monteiro, S.H.N.; Smidt, E.C. O gênero *Galeandra* (Orchidaceae: Catasetinae) no estado do Paraná, Brasil. *Rodriguésia* **2015**, *66*, 221–227.

Bolson, M., Toscano de Brito, A.L.V.; Smidt, E.C. A neglected name and new synonym in *Barbosella* (Pleurothallidinae, Orchidaceae). *Phytotaxa* **2018**, *340*, 246–254.

Borba, E.L., Felix, J.M., Semir, J.; Solferini, V.N. *Pleurothallis fabiobarrosii*, a new Brazilian species: morphological and genetic data with notes on the taxonomy of Brazilian rupicolous *Pleurothallis*. *Lindleyana* **2000**, *15(1)*, 2–9.

Borba, E.L.; Smidt, E.C. *Bulbophyllum filifolium* (Orchidaceae), a new species from southeastern Brazil. *Novon* **2004**, *14*, 29–32.

Borba, E.L. Novas combinações em *Acianthera* (*Pleurothallis s.l.*; Orchidaceae: Pleurothallidinae) ocorrentes nos campos rupestres brasileiros. *Sitientibus, ser. Ciênc. Biol.* **2003**, *3(1/2)*, 22–25.

Borba, E.L.;Funch, R.R.; Ribeiro, P.L.; Smidt, E.C.; Silva-Pereira, V. Demografia, variabilidade genética e morfológica e conservação de *Cattleya tenuis* (Orchidaceae), espécie ameaçada de extinção da Chapada Diamantina. *Sitientibus sér. Ciênc. Biol.* **2007**, *7*, 211–222.

Borba, E.L.; Funch, R.R.; Ribeiro, P.L.; Smidt, E.C.; Silva-Pereira, V. Demography, and genetic and morphological variability of the endangered *Sophronitis sincorana* (Orchidaceae) in the Chapada Diamantina, Brazil. *Pl. Syst. Evol.* **2007**, *267*, 129–146. https://doi.org/10.1007/s00606-007-0555-9.

Borba, E.L.; Salazar, G.A.; Mazzoni-Viveiros, S.; Batista, J.A.N. Phylogenetic position and floral morphology of the Brazilian endemic, monospecific genus *Cotylolabium*: a sister group for the remaining Spiranthinae (Orchidaceae). *Bot. J. Linn. Soc.* **2014**, *175*, 29–46. https://doi.org/10.1111/boj.12136.

Borba, E.L.; Shepherd, G.J.; van den Berg, C.; Semir, J. Floral and vegetative morphometrics of five *Pleurothallis* (Orchidaceae) species: correlation with taxonomy, phylogeny, genetic variability and pollination systems. *Ann. Bot.* **2002**, *90*, 1–12. https://doi.org/10.1093/aob/mcf168.

Brieger, F.G. Geographic distribution and phylogeny of orchids. *Proceedings of the 3rd World Orchid Conference, London.* Royal Horticulture Society, **1960**, pp. 328–333.

Brieger, F.G. Dispersão e migração na evolução das orquídeas americanas. *Publ. Cien. Inst. Gen.* **1961**, *2*, 69–82.

Buzatto, C.R., Freitas, E.M., Silva, A.P.M.; Lima, L.F.P. Levantamento da família Orchidaceae ocorrentes na Fazenda São Maximiano, município de Guaíba, Rio Grande do Sul. *Rev. Bras. Biociênc.* **2007**, *5(2/3)*, 19–25.

Buzatto, C.R., Ferreira, P.P.A, Welker, C.A.D., Seger, G.D.S., Hertzog, A.; Singer, R.B. O gênero *Cattleya* Lindl. (Orchidaceae: Laeliinae) no Rio Grande do Sul, Brasil. *Rev. Bras. Biociênc.* **2010**, *8(4)*, 388–398.

Buzatto, C.R., Singer, R.B.; van den Berg, C. O gênero *Capanemia* Barb. Rodr. (Oncidiinae: Orchidaceae) na Região Sul do Brasil. *Rev. Bras. Biociênc.* **2010**, *8(4)*, 309–323.

Buzatto, C.R.; Machado, M.C. *Capanemia* Barb. Rodr. (Orchidaceae: Oncidiinae), a new record from Bahia state, Brazil. *Acta Bot. Bras.* **2011**, *25*, 249–251.

Buzatto, C.R., Singer, R.B., Romero-González, G.A.; van den Berg, C. Typifications and new synonyms in *Capanemia* (Orchidaceae, Oncidiinae). *Novon* **2011**, *21(1)*, 28–33.

Buzatto, C.R., Singer, R.B., Romero-González, G.A., van den Berg, C.; Salazar, G.A. Typifications and taxonomic notes in species of Brazilian Goodyerinae and Spiranthinae (Orchidaceae) described by José Vellozo and Barbosa Rodrigues. *Taxon* **2013**, *62(3)*, 609–621.

Buzatto, C.R.; Sanguinetti, A.; Romero-González, G.A.; van den Berg, C.; Singer, R.B. A taxonomic synopsis of Brazilian Chloraeinae (Orchidaceae: Orchidoideae). *Phytotaxa* 2014, *158*, 1–22. https://doi.org/10.11646/phytotaxa.158.1.1.
Cantuaria, P.C., Krahl, D.R.P., Krahl, A.H., Chiron, G.,; Silva, J.B.F. The *Catasetum× sheyllae* (Orchidaceae: Catasetinae), a new natural hybrid from the Brazilian Amazon. *Phytotaxa* **2021**, *527(4)*, 257–265.

Cantuaria, P.C., Krahl, D.R.P., Schmal, P., Silva, J.B.F., Damasceno, T.S., Petcov, A.P., Krahl, A.H.; Chiron, G. A new form of *Galeandra styllomisantha* (Orchidaceae: Catasetinae) from the Brazilian Amazon. *Richardiana* **2024**, *8*, 8–20.

Camelo-Jr., A.E.; Ferreira, A.W.C.; Andrade, I.M.; Mayo, S.J.; Nollet, F.; Silva, J.L.; Barros, M.C.; Fraga, E.; Pessoa, E. M. Species delimitation in the *Trichocentrum cepula* (Oncidiinae, Orchidaceae) complex: a multidisciplinary approach. *Syst. Biodiv.* **2022**, *20*, 1–18. https://doi.org/10.1080/14772000.2022.2099478.

Campacci, M.A.; Romero-Gonzáles, G.; Silva, J.B.F. Novas espécies (e híbridos naturais). *Colet. Orq. Bras.* **2007**, *5*, 129–168.

Campacci, M.A.; Carr Jr., J.F.; Silva, J.B.F. Novas espécies (e um híbrido natural). *Colet. Orq. Bras.* **2008**, *6*, 169–208.

Campacci, M.A.; Bohnke, E.; Carr Jr., J.F.; Silva, J.B.F.; Campacci, T.V.S.; Laitano, T.L. Novas espécies (e um híbrido natural). *Colet. Orq. Bras.* **2009**, *7*, 209–260.

Campacci, M.A.; Bohnke, E.; Carr Jr., J.F.; Silva, J.B.F.; Oliveira, S.M. Novas espécies. *Colet. Orq. Bras.* **2010**, *8*, 261–308.

Campacci, M.A.; Docha Neto, A.; Baptista, D.H.; Leitão, F.D.V.; Harding, P.; Castro Neto, V.P.. Gêneros, espécies e híbridos naturais novos.. *Colet. Orq. Bras.* **2011**, *9*, 309–355.

Campacci, M.A.; Barrios, C.F.E.; Bohnke, E.; Carr Jr., G.F. Novas espécies (e híbridos naturais). *Colet. Orq. Bras.* **2014**, *10*, 357–404.

Campacci, M.A.; Bohnke, E.; Carr Jr., G.F., Silva, J.B.F.; Harding, P.A. Novas espécies. *Colet. Orq. Bras.* **2015**, *11*, 405–452.

Campacci, M.A.; Portalet, A.A.; Silva, J.B.F.; Moreno, J.S.; Locatelli, M.; Rosim, M.S.;Harding, P.A. Novas espécies. *Colet. Orq. Bras.* **2016**, *12*, 453–504.

Campacci, M.A.; Menezes, E.L.F.; Rosim, M.S.; Harding, P.A. Novas espécies (e híbridos naturais). *Colet. Orq. Bras.* **2018**, *13*, 505–544.

Campacci, M.A.; Silva, C.R.M.; Menezes, E.L.F.; Rosim, M.S.; Harding, P.A.; Castro Neto, V.P. Novas espécies (e híbridos naturais). *Colet. Orq. Bras.* **2018**, *14*, 545–584.

Campacci, M.A.; Silva, C.R.M.; Bohnke, E.;Menezes, E.L.F.; Silva, J.B.F.; Rosim, M.S.; Harding, P.A.; Tobias, S.L.X.; Ferreira, U.L.C. Novas espécies (e híbridos naturais). *Colet. Orq. Bras.* **2019**, *15*, 585–636.

Campacci, M.A.; Leitão, F.D.V.; Silva, C.R.M.; Rosim, M.S.; Harding, P.A.; Tobias, S.L.X. Novas espécies (e híbridos naturais). *Colet. Orq. Bras.* **2020**, *16*, 637–680.

Campacci, M.A.; Silva, C.R.M.; Gerlach, G.; Silva, J.B.F.; Rosim, M.S.; Harding, P.A.; Giória, R. Novas espécies (e combinações nomenclaturais). *Colet. Orq. Bras.* **2021**, *17*, 681–716.

Carlini-Garcia, L.; van den Berg, C.; Martins, P.S. A morphometric analysis of floral characters in *Miltonia spectabilis* and *Miltonia spectabilis* var. *moreliana* (Maxillarieae: Oncidiinae). *Lindleyana* **2002**, *17(3)*, 122–129.

Carneiro-Silva, M.Q., Koch, A.K., Viana, P.L.; Ilkiu-Borges, A.L. Oncidiinae (Orchidaceae) on the great curve of the Xingu River, Pará state, Brazil. *Braz. J. Biol.* **2015**, *75*, 222–237.

Carvalho, B.M., Ramalho, A.J.; Batista, J.A.N. O gênero *Habenaria* (Orchidaceae) na Serra da Canastra, Minas Gerais, Brasil. *Rodriguésia* **2013**, *64*, 223–245. https://doi.org/10.1590/S2175-78602013000200003.

Carvalho, D.N., Meneguzzo, T.E.C., Popovkin, A.; van den Berg, C. Orchidaceae of Bahia, Brazil: notes on taxonomy and nomenclature. *Phytotaxa* **2016**, *272(3)*, 231–234.

Carvalho, D.N., Meneguzzo, T.E.C.; van den Berg, C. *Vanilla calamitosa* (Orchidaceae), a new aphyllous species from eastern Brazil. *Kew Bull.* **2023**, *78*, 309–317.

Ceschin, F., Mancinelli, W.S.; Smidt, E.C. Flora Fanerogâmica do Estado do Paraná, Brasil: *Psilochilus* Barb. Rodr. (Orchidaceae, Triphorinae). *Acta Biol. Parana.* **2012**, *41*, 119–126.

Chacur, F. Análise da variação e da taxonomia no gênero *Brassavola* R.Br. (Orchidaceae – Epidendroideae). PhD dissertation, Universidade de São Paulo. Ribeirão Preto, 1973, 101 p.

Chiron, G.R. Riqueza e endemismo de espécies de *Baptistonia* (Orchidaceae), no Brasil. *Hoehnea* **2009**, *36*, 459–477.

Chiron, G.R.; Oliveira, R.P.; Santos, T.M.; Bellvert, F.; Bertrand, C.; van den Berg, C. Phylogeny and evolution of *Baptistonia* (Orchidaceae, Oncidiinae) based on molecular analyses, morphology and floral oil evidence. *Pl. Syst. Evol.* **2009**, *281(1)*, 35–49.

Chiron, G.R.; van den Berg, C. Révision taxinomique du genre *Acianthera* (Orchidaceae, Pleurothallidinae). *Richardiana* **2012**, *12*, 59–77.

Chiron, G.R.; Guiard, J.; van den Berg, C. Phylogenetic relationships in Brazilian *Pleurothallis* sensu lato (Pleurothallidinae, Orchidaceae): evidence from nuclear ITS rDNA sequences. *Phytotaxa* **2012**, *46*, 34–58.

Colla, F.B.; Waechter, J.L. O gênero *Stelis* Sw. (Orchidaceae) no Rio Grande do Sul, Brasil. *Rev. Bras. Biociênc.* **2013**, *11(1)*, 119–129.

Cruz, D.T., Borba, E.L.; van den Berg, C. O gênero *Cattleya* Lindl. (Orchidaceae) no estado da Bahia, Brasil. *Sitientibus, ser. Ciênc. Biol.* **2003**, *3(1/2)*, 26–34.

Cruz, D.T.; Selbach-Schnadelbach, A.; Lambert, S.M.; Ribeiro, P.L.; Borba, E.L. Genetic and morphological variability in *Cattleya elongata* Barb. Rodr. (Orchidaceae), endemic to the campo rupestre vegetation in northeastern Brazil. *Pl. Syst. Evol.* **2011**, *294*, 87–98. https://doi.org/10.1007/s00606-011-0444-0.

Cruz-Lustre, G., Castro, C., Borba, E.L.; Batista, J.A.N. Phylogenetics and taxonomy of *Habenaria* sect. *Micranthae* (Orchidaceae), with the description of an overlooked new species from the Espinhaço mountain range, Eastern Brazil. *Syst. Biodiv.* **2022**, *20*, 1–20. https://doi.org/10.1080/14772000.2022.2030424.

Cunha, M.F.B.; Forzza, R.C. 2007. Orchidaceae no Parque Natural Municipal da Prainha, RJ, Brasil. *Acta Bot. Bras.* **2007**, *21*, 383–400.

Engels, M.E.; Tardivo, R.C. O gênero *Isabelia* (Orchidaceae: Laeliinae) no estado do Paraná, Brasil. *Rodriguésia* **2013**, *64*, 369–377.

Engels, M.E.; Rocha, L.C.F. *Vanilla appendiculata* (Orchidaceae): primeiro registro para o estado do Mato Grosso, Brasil. *Rodriguésia* **2016**, *67(3)*, 855–858.

Engels, M.E.; Smidt, E.C. Orchidaceae de um fragmento campestre em Ponta Grossa, Paraná, Brasil. *Acta Biol. Parana.* **2016**, *45*, 11–19.

Engels, M.E., Barros, F.; Smidt, E.C. A subtribo Goodyerinae (Orchidaceae: orchidoideae) no estado do Paraná, Brasil. *Rodriguésia* **2016**, *67*, 917–952.

Engels, M.E., Rocha, L.C.F.; Petini-Benelli, A. A new species of *Catasetum* (Orchidaceae, Epidendroideae, Cymbidieae) from the Southern Brazilian Amazon. *Lankesteriana* **2016**, *16*, 329–333.

Engels, M.E.; Rocha, L.C.F. *Macroclinium chasei* (Orchidaceae, Oncidiinae): a new record for Brazil. *Lankesteriana* **2016**, *17*, 369–373.

Engels, M.E.; Rocha, L.C.F. *Maxillaria aureoglobula* (Orchidaceae, Maxillariinae): a new record from Brazil. *Lankesteriana* **2016**, *16*, 119–122.

Engels, M.E.; Rocha, L.C.F. Novos registros de distribuição geográfica em *Sobralia* (Orchidaceae: Epidendroideae) para a Região Centro-Oeste do Brasil. *Rodriguésia* **2017**, *68(4)*, 1483–1486.

Engels, M.E.; Rocha, L.C.F. Dois novos registros de distribuição geográfica em *Epidendrum* (Orchidaceae) para o Centro-Oeste brasileiro. *Rodriguésia* **2017**, *68*, 779–782.

Engels, M.E., Rocha, L.C.F.; Pessoa, E.M. O gênero *Coryanthes* (Orchidaceae-Stanhopeinae) no estado do Mato Grosso, Brasil. *Rodriguésia* **2017**, *68*, 489–501.

Engels, M.E., Rocha, L.C.F., Hall, C.F.; Koch, A.K. Primeiro registro de *Aganisia fimbriata* (Orchidaceae: Zygopetalinae) para a região Centro-Oeste do Brasil. Hoehnea **2018**, *45(2)*, 348–351.

Engels, M.E., Rocha, L.C.F.; Pessoa, E.M. Novo registro de *Paphinia* (Orchidaceae: Stanhopeinae) para a Região Centro-Oeste brasileira, estado de Mato Grosso. *Rodriguésia* **2018**, *69*, 2253–2257.

Engels, M.E., Salgado, T.L.; Batista, J.A.N. A spectacular new species of *Habenaria* (Orchidaceae) from southern Brazilian Amazon. *Syst. Bot.* **2019**, *44*, 775–782. https://doi.org/10.1600/036364419X15710776741387.

Engels, M.E., Rocha, L.C.F., Koch, A.K.; Gerlach, G. O gênero *Gongora* (Orchidaceae, Stanhopeinae) no estado de Mato Grosso, Brasil. *Rodriguésia* **2020**, *71*, e03132018.

Engels, M.E., Dias, D.C.; Koch, A.K. Increased morphological variation and a new record of *Vanilla labellopapillata* (Orchidaceae) from the Mato Grosso State, Brazil. *Phytotaxa* **2020**, *472*, 63–68.

Engels, M.E., Rocha, L.C.F.; Koch, A.K. Novidades em *Vanilla* Mill. (Orchidaceae) para a borda sul-amazônica, Estado de Mato Grosso, Brasil. *Hoehnea* **2020,** *47*, e032020.

Engels, M.E., Rocha, L.C.F.; Koch, A.K. Primeiro registro de *Chaubardia surinamensis* (Orchidaceae: Zygopetalinae) para o Mato Grosso, Brasil. *Acta Biol. Parana.* **2022**, *51*, 1–5. https://doi.org/10.5380/abp.v51i1.85594.

Engels, M.E., Rocha, L.C.F.; Smidt, E.C. A new species of *Mormodes* (Orchidaceae: Catasetinae) from southern Amazon, Brazil. *Braz. J. Bot.* 2020, *43(3)*, 555–561.

Engels, M.E.; Koch, A.K. *Vanilla ribeiroi* Hoehne (Orchidaceae: Vanilloideae): notes on taxonomy and geographical distribution. *Phytotaxa* **2021**, *490*, 99–106.

Engels, M.E., Silva, C.A.; Koch, A.K. The entity of the enigmatic Hoehne's *Sobralia cataractarum* (Orchidaceae) clarified. *Phytotaxa* **2021**, *525*, 247–250.

Engels, M.E., Smidt, E.C., Silva, C.A.; Koch, A.K. *Mormodes benelliana* (Catasetinae), a new species from Mato Grosso, Brazil. *Lankesteriana* **2024**, *24*, 222–231.

Engels, M.E.; Smidt, E.C. A new *Sarcoglottis* (Orchidaceae: Spiranthinae) from the southern Brazilian Amazon. *Phytotaxa* **2021**, *487*, 171–176.

Engels, M.E.; Smidt, E.C. Taxonomic and nomenclatural notes in *Microchilus longicornu* (Goodyerinae Orchidaceae). *Phytotaxa* **2022**, *568*, 296–300.

Engels, M.E.; Smidt, E.C. *Microchilus dasilvae*: a new species of Goodyerinae (Orchidaceae) from Southeastern Brazil. *Phytotaxa* **2022**, *555*, 252–258.

Engels, M.E., Carvalho, B.H.G., Liebsch, D.; Smidt, E.C. 2022. Novo registro do gênero *Bletia* Ruiz; Pav. (Orchidaceae: Epidendroideae: Bletiinae) no Estado do Paraná, Brasil. *Acta Biol. Parana.* **2022**, *51*, 1–6.

Engels, M.E.; Smidt, E.C. *Maxillaria luizotavioi* (Orchidaceae: Maxillariinae): a new species from the Oriental Brazilian Amazon. *Nordic J. Bot.* **2023**, e03815

Engels, M.E., Silva-Pereira, V.; Smidt, E.C. *Eltroplectris paranaensis*: a new species of Spiranthinae (Orchidaceae) from the nebular Atlantic Rain Forest. *Phytotaxa* **2023**, *609*: 115–123.

Engels, M.E.; Smidt, E.C. *Microchilus culuenensis* (Orchidaceae, Goodyerinae): a new species from Southearn Brazilian Amazon. *Phytotaxa* **2024**, *644*, 56–62.

Estevinho, T.F., Toscano de Brito, A.L.V.; Smidt, E.C. O gênero *Ornithocephalus* Hook. (Orchidaceae: Oncidiinae) nos estados do Paraná e Santa Catarina. *Acta Biol. Parana.* **2016**, *45*, 1–9.

Fajardo, C.G.; Vieira, F.A.; Molina, W.F. Interspecific genetic analysis of orchids in Brazil using molecular markers. *Pl. Syst. Evol.* **2014**, *300*, 1825–1832. https://doi.org/10.1007/s00606-014-1009-9.

Ferreira, U.L.C.; Filho, R.M.C Two new natural hybrids in *Catasetum* (Orchidaceae) from Brazil. *Richardiana* **2019**, *3*, 39–49.

Ferreira, A.W.C., Baptista, D.H.; Pansarin, E.R. *Triphora uniflora* A.C.Ferreira, Baptista, Pansarin (Orchidaceae: Triphoreae): uma nova espécie e primeiro registro do gênero *Triphora* Nutt. para o estado de São Paulo, Brasil. *Acta Bot. Bras.* **2010**, *24*, 288–291.

Ferreira, A.W.C., Lima, M.I.S.; Pansarin, E.R. Orchidaceae in the central portion of São Paulo State, Brazil. *Rodriguésia* **2010**, *61*, 243–259.

Ferreira, A.W.C., Lima, M.I.S.; Pansarin, E.R. A new species of *Pelexia* (Orchidaceae, Spiranthinae) from São Paulo, Brazil. *Novon* **2012**, *22(2)*, 155–159.

Ferreira, A.W.C., Oliveira, M.S., Silva, E.O., Campos, D.S., Pansarin, E.R.; Guarçoni, E.A.E. *Vanilla bahiana* Hoehne and *Vanilla pompona* Schiede (Orchidaceae, Vanilloideae): two new records from Maranhão state, Brazil. *Check List* **2017**, *13(6)*, 1131–1137.

Ferreira, A.W.C., Oliveira, M.S., Engels, M.E.; Pessoa, E.M. Found in Brazil again! Expanding the distribution of *Maxillaria aureoglobula* Christenson (Orchidaceae, Maxillariinae) and a key to the species of *Maxillaria* sect. *Rufescens* Christenson from Brazil. *Check List* **2019**, *15*, 1107–1112.

Ferreira, A.W.C., Franken, E.P.; Pansarin, E.R. Confirmation of the presence of *Vanilla hartii* Rolfe (Orchidaceae, Vanilloideae) in Brazil. *Check List* **2020**, *16(4)*, 951–956.

Fiorini, C.F.; Borba, E.L.; Resende-Moreira, L.C.; Smidt, E. C.; Knowles, L.L. Geographic isolation alone does not explain divergence of a group of orchid species across Brazil's campos rupestres sky-islands. *Evolution* **2023**, *77*, 946–958. https://doi.org/10.1093/evolut/qpad010.

Forster, W. Estudo taxonômico das espécies com folhas planas a conduplicadas do gênero *Octomeria* R.Br. (Orchidaceae). PhD dissertation, Universidade Estadual de Campinas, 2007.

Forster, W.; Souza, V.C. *Epidendrum caparaoense* (Orchidaceae), a new species from Minas Gerais, Brazil. *Bot. J. Linn. Soc.* **2007**, *155(1)*, 157–159.

Forster, W.; Souza, V.C. Laeliinae (Orchidaceae) do Parque Nacional do Caparaó, Estados do Espírito Santo e Minas Gerais, Brasil. *Hoehnea* **2013**, *40*, 701–726.

Forster, W., Souza, V.C.; Barros, F. *Octomeria lilliputana* (Orchidaceae), a new species from Brazilian Atlantic Forest, state of Paraná, Brazil. *Phytotaxa* **2013**, *105(2)*, 39–44. https://doi.org/10.11646/phytotaxa.105.2.2.

Fraga, C.N.; Peixoto, A.L. Florística e ecologia das Orchidaceae das restingas do estado do Espírito Santo. *Rodriguésia* **2004**, *55*, 5–20.

Fraga, C.N. Notas taxonômicas para espécies brasileiras de *Vanilla* Mill. (Orchidaceae). Bol. Mus. Biol. Mello Leitão **2002**, *13*, 45–52.

Fraga, C.N.; Smidt, E.C. *Bulbophyllum arianeae* (Orchidaceae), a new species from the Atlantic Forest of Espírito Santo, Brazil. *Harv. Pap. Bot.* **2004**, *9(1)*, 7–9.

Fraga, C.N.; Kollmann, L.J.C. Three new species of *Pabstiella* (Pleurothallidinae, Orchidaceae) from Brazilian Atlantic Forest. *Harv. Pap. Bot.* **2010**, *15(1)*, 171–178.

Gomes, G.D.S., Ferreira, A.W.C., Silva, M.J.C.D., Conceição, G.M.D.; Pessoa, E.M.Taxonomic study of *Epidendrum* (Laeliinae–Orchidaceae) in the state of Maranhão, northeastern Brazil. *Rodriguésia* **2021**, *72*, e01492020.

Gomes, P.C.L.; Smidt, E.C.; Fraga, C.N.; Silva-Pereira, V. High genetic variability is preserved in relict populations of *Cattleya lobata* (Orchidaceae) in the Atlantic Rainforests inselbergs. *Braz. J. Bot.* **2018**, *41*, 185–195. https://doi.org/10.1007/s40415-017-0422-z.

Gomes, S.S.; Vidal, J.D.; Neves, C.S.; Zorzatto, C.; Campacci, T.V.S.; Lima, A.K.; Koehler, S.; Viccini, L.F. Genome size and climate segregation suggest distinct colonization histories of an orchid species from Neotropical high-elevation rocky complexes. *Biological Journal of the Linnean Society*, **2018**, *124*, 456–465. https://doi.org/10.1093/biolinnean/bly065.

Gonçalves, C.N.; Waechter, J.L. Sinopse do gênero *Acianthera* Scheidw. (Orchidaceae) no Rio Grande do Sul, Brasil. *Rev. Bras. Biociênc.* **2011**, *9(2)*, 143–155.

Goncalves, G.F., Mauad, A.V.S.R., Taques, G., Smidt, E.C.; Barros, F. Molecular and morphological phylogenetic analysis and taxonomic revision of the genus *Orleanesia* (Laeliinae, Epidendroideae, Orchidaceae). *Phytotaxa* **2019**, *392*, 1–18.

Guimarães, L.R.S.; Salazar, G.; Barros, F. Lectotypifications and taxonomic notes in the *Stenorrhynchos* clade (Spiranthinae, Orchidaceae). *Phytotaxa* **2019**, *394*, 111–117. https://doi.org/10.11646/phytotaxa.394.1.9.

Gutiérrez-Morales, N., Toscano de Brito, A.L.V.; Smidt, E.C. *Pabstiella hileiaensis* (Orchidaceae Pleurothallidinae), a new species from the Atlantic Rainforest in southern Bahia, Brazil. *Phytotaxa* **2021**, *518*, 61–68.

Gutiérrez-Morales, N., Toscano de Brito, A.L.V.; Smidt, E.C. Novelties and nomenclatural notes in *Pabstiella* (Orchidaceae) from the Brazilian Atlantic Rainforest of Bahia and Espírito Santo. *Phytotaxa* **2023**, *625*, 161–179.

Gutiérrez-Morales, N., Toscano de Brito, A.L.V., Mauad, S.R., Victoria, A.; Smidt, E.C. Molecular phylogeny and biogeography of *Pabstiella* (Pleurothallidinae: Orchidaceae) highlight the importance of the Atlantic Rainforest for speciation in the genus. *Bot. J. Linn. Soc.* **2021**, *195(4)*: 568–587.

Hagsater, E.; Krahl, A.H. *Epidendrum deditae*, a new species of Orchidaceae (Laeliinae) from northern Brazil. *Phytotaxa*, **2020**, *440(3)*, 225–231.

Hall, C.F. Orchidaceae do Parque Estadual da Serra de Caldas Novas, Goiás, Brasil. J. Neotrop. Biol. **2009**, *6(1)*, 87–88.

Hall, C.F., Klein, V.L.G.; Barros, F. Orchidaceae no município de Caldas Novas, Goiás, Brasil. *Rodriguésia* 2013, *64*, 685–704.

Hall, C.F., Koch, A.K., Francener, A.; Barros, F. First record of the genus *Cranichis* Sw. (Orchidaceae) for the state of Pará, Brazil. *Bol. Mus. Paraense Emílio Goeldi* **2014**, *9*: 233–236.

Hall, C.F., Koch, A.K., Vieira, T.L.; Barros, F. *Koellensteinia dasilvae* sp. nov. (Zygopetalinae-Orchidaceae) from the Brazilian Amazon. *Nordic J. Bot.* **2015**, *33(6)*, 729–732. https://doi.org/10.1111/njb.00864.

Heberle, W., Freitas, E.M.D.; Jasper, A. A família Orchidaceae no Jardim Botânico de Lajeado, Rio Grande do Sul, Brasil. *Pesquisas, Bot.* **2012**, *3*, 189–199.

Ignowski, H., Toscano de Brito, A.L.V., Bona, C.; Smidt, E.C. Nomenclatural notes on *Stelis ciliaris* Lindl. (Orchidaceae: Pleurothallidinae). *Phytotaxa* **2015**, *218*, 30–38.

Illg, R.D. Aspectos evolutivos em algumas *Maxillarias* brasileiras. PhD dissertation, Universidade Estadual de Campinas, Campinas, 1975.

Imig, D.C., Mancinelli, W.S.; Smidt, E.C. A new *Dryadella* Luer (Orchidaceae: Pleurothallidinae) from the South Atlantic Rainforest. *Phytotaxa* **2021**, *508*, 1–7.

Imig, D.C., Mauad, A.V.S.R., Silva-Pereira, V., Toscano de Brito, A.L.V.; Smidt, E.C. Taxonomic update of *Dryadella* (Orchidaceae: Pleurothallidinae) based on morphometric analyses of three species of the Brazilian Atlantic rainforest. *Feddes Repert.* **2023**, *134*, 66–79.

Imig, D.C., A.L.V.T.; Smidt, E.C.The genus *Dryadella* (Orchidaceae, Pleurothallidinae) in Brazil. *Rodriguésia* **2024**, *75*, 1–43.

Imig, D.C., Engels, M.E.; Smidt, E.C. New synonyms in *Dryadella* (Orchidaceae: Pleurothallidinae) from the Brazilian Atlantic Forest. *Acta Biol. Parana.* **2025**, *54*, e98025.

Klein, V.P.; Pessoa, E.M.; Demarchi, L.O.; Sader, M.; Piedade, M.T.F. *Encyclia, Epidendrum*, or *Prosthechea*? Clarifying the phylogenetic position of a rare Amazonian orchid (Laeliinae-Epidendroideae-Orchidaceae). *Syst. Bot.* **2019**, *44*, 297–309. https://doi.org/10.1600/036364419X15562054132983.

Koehler, S.; Amaral, M.C.E. A taxonomic study of the South American genus *Bifrenaria* Lindl. (Orchidaceae). *Brittonia* **2004**, *56*, 314–345. https://doi.org/10.1663/0007-196X(2004)056[0314:ATSOTS]2.0.CO;2.

Koehler, S.; Cabral, J.S.; Whitten, W.M.; Williams, N.H.; Singer, R.B.; Neubig, K.M.; Guerra, M.; Souza, A.P.; Amaral, M.C.E. Molecular phylogeny of the Neotropical genus *Christensonella* (Orchidaceae, Maxillariinae): species delimitation and insights into chromosome evolution. *Ann. Bot.* **2008**, *102*, 491–507. https://doi.org/10.1093/aob/mcn128.

Koehler, S.; Singer, R.B.; Amaral, M.C.E. Taxonomic revision of the Neotropical genus *Christensonella* (Maxillariinae, Orchidaceae). *Bot. J. Linn. Soc.* **2012**, *168*, 449–472. https://doi.org/10.1111/j.1095-8339.2012.01218.x.

Koehler, S.; Williams, N.H.; Whitten, W.M.; Amaral, M.C.E. Phylogeny of the *Bifrenaria* (Orchidaceae) complex based on morphology and sequence data from nuclear rDNA internal transcribed spacers (ITS) and chloroplast *trnL-trnF* region. *Int. J. Pl. Sci.* **2002**, *163*, 1055–1066. https://doi.org/10.1086/342035.

Koch, A.K., Fraga, C.N., Santos, J.U.M.; Ilkiu-Borges, A.L. Taxonomic notes on *Vanilla* (Orchidaceae) in the Brazilian Amazon, and the description of a new species. *Syst. Bot.* **2013**, *38(4)*, 975–981.

Koch, A.K., Santos, J.U.M.; Ilkiu-Borges, A.L. Sinopse das Orchidaceae holoepífitas e hemiepífitas da Floresta Nacional de Caxiuanã, PA, Brasil. *Hoehnea* **2014**, *41*, 129–148. https://doi.org/10.1590/S2236-89062014000100012.

Koch, A.K., Hall, C.F., Smidt, E.C., Almeida, C.B.R., Bolson, M.; Silva, C.A. First record of the genus *Tropidia* Lindl. (Orchidaceae) for Brazil. *Hoehnea* **2016**, *43*, 521–528.

Koch, A.K., Miranda, J.C., Hall, C.F. Flora das cangas da Serra dos Carajás, Pará, Brasil: Orchidaceae. *Rodriguésia* **2018**, *69*, 165–188.

Kollmann, L.J.C. *Acianthera fornograndensis* L. Kollmann; AP Fontana (Orchidaceae), uma nova espécie da Floresta Atlântica do Estado do Espírito Santo, Brasil. *Bol. Mus. Biol. Mello Leitão* **2006**, *20*, 27–31.

Kollmann, L.J.C. *Specklinia fragae* Kollmann & Fontana (Orchidaceae), uma nova espécie da Mata Atlântica do Espírito Santo, Brasil. *Bol. Mus. Biol. Mello Leitão* **2008**, *23*, 15–19.

Kollmann, L.J.C. New combinations and description of two new species in *Pabstiella* Brieger; Senghas (Orchidaceae) from Brazil. *Candollea* **2010**, *65(1)*, 95–100.

Krahl, A.H., Cogo, A.J.D.; Valsko, J.J. Orchidaceae em um fragmento de Floresta Semidecídua de encosta na região sul do Estado do Espírito Santo, Sudeste do Brasil. *Hoehnea* **2014**, *41*, 247–268.

Krahl, A.H., Valsko, J.J., Holanda, A.S.S.; Chiron, G. A new species of *Dichaea* (Orchidaceae) from Brazil with special reference to its foliar anatomy. *Phytotaxa* **2016,** *265(2)*, 145. https://doi.org/10.11646/phytotaxa.265.2.6.

Krahl, A.H., Krahl, D.R.P., Cantuária, P.C.; Silva, J.B.F.*Catasetum saracataquerense* (Orchidaceae, Catasetinae), a new species of Brazilian Amazon. *Richardiana* **2021**, *5*, 206–216.

Krahl, A.H., Cordeiro, J.M.; Hagsater, E.*Epidendrum dayseae*, a new species of Orchidaceae (Laeliinae) from northern Brazil. *Phytotaxa* **2022**, *530(1)*: 95–102.

Krahl, D.R.P., Krahl, A.H.; Chiron, G.*Catasetum ×louisiae* (Orchidaceae: Catasetinae), a new natural hybrid for the Brazilian Amazon. *Richardiana* **2020**, *4*, 214–223.

Krahl, D.R.P., Oliveira, M.S., Silva, J.B.F., Chiron, G.; Cantuária, P.C. *Catasetum krahlii* (Orchidaceae, Catasetinae): a new and threatened species from the Brazilian Amazon. *Acta Bot. Bras.* **2023**, *37*: e20220258.

Krahl, D.R.P., Schmal, P., Chiron, G., Silva, J.B.F., Krahl, A.H.; Cantuária, P.C. Taxonomic notes on *Catasetum meeae* (Orchidaceae: Catasetinae). *Phytotaxa* **2023**, *609(3)*, 240–246.

Krahl, D.R.P., Schmal, P., Chiron, G., Silva, J.B.F., Krahl, A.H.; Cantuaria, P.C.*Catasetum× grasineideae* (Orchidaceae: Catasetinae), a new nothospecies from Brazilian Amazon and taxonomic notes for the genus. *Phytotaxa* **2023**, *594(2)*, 89–104.

Krahl, D.R.P., Chiron, G., Silva, J.B.F., Krahl, A.H.; Cantuária, P.C.*Catasetum dianneae* (Orchidaceae, Catasetinae): a new species of the *C. barbatum* complex for the Brazilian Amazon. *Richardiana* **2023**, *7*, 187–201.

Krahl, D.R.P., Schmal, P., Chiron, G., Silva, J.B.F., Krahl, A.H.; Cantuaria, P.C. *Catasetum queirozii* (Orchidaceae: Catasetinae): a new species from the Brazilian Amazon. *Acta Amaz.* **2024**, *54*: e54bc23180.

Krahl, D.R.P.; Oliveira, M.S.; Schmal, P.; Chiron, G.; Krahl, A.H.; Silva, J.B.F.; Cantuária, P.C. Revealing the true taxonomic status of *Catasetum joaquinianum* (Orchidaceae: Catasetinae). *Phytotaxa* **2024**, *664*, 123–131. https://doi.org/10.11646/phytotaxa.664.2.4.

Krahl, D.R.P., Silva, J.B.F., Damián-Parizaca, A., Flanagan, N.S., Chiron, G., Krahl, A.H., ...; Pansarin, E.R. Revelando a identidade taxonômica de *Vanilla sprucei* (Orchidaceae, Vanilloideae): sua posição filogenética e um novo registro para o Brasil. *Acta Amaz.* **2025**, *55*, e55bc23227. https://doi.org/10.1590/1809-4392202302272.

Lara, M.; Pessoa, E. Synopsis of *Epidendrum* (Laeliinae) from the state of Mato Grosso, Brazil: taxonomy and distribution. *Lankesteriana* **2025**, *25*, 21–42.

Lau, B.L.; Batista, J.A.N.; Massensini-Junior, A.; Whitten, W.M.; Borba, E.L. Unravelling the *Habenaria repens* (Orchidaceae) complex in Brazil: a biosystematic and molecular phylogenetic approach. *Bot. J. Linn. Soc.* **2021**, *197*, 229–248. https://doi.org/10.1093/botlinnean/boab022.

Leal, B.S.; Ambrosano, G.B.; Margarido, G.R.A.; Palma-Silva, C.; Pinheiro, F. Interploidy gene flow does not prevent adaptive genetic differentiation in sympatric populations of *Epidendrum fulgens* and *E. puniceoluteum* (Orchidaceae). *Bot. J. Linn. Soc.* **2025**, boaf015.

Leal, B.S.; Brandão, M.M.; Palma-Silva, C.; Pinheiro, F. Differential gene expression reveals mechanisms related to habitat divergence between hybridizing orchids from the Neotropical coastal plains. *BMC Pl. Biol.* **2020**, *20*, 554. http://dx.doi.org/10.1186/s12870-020-02757-x.

Leal, B.S.; Chaves, C.J.; Koehler, S.; Borba, E.L. When hybrids are not hybrids: a case study of a putative hybrid zone between *Cattleya coccinea* and *C. brevipedunculata* (Orchidaceae). *Bot. J. Linn. Soc.* **2016**, *181*, 621–639.

Leles, B.; Chaves, A.V.; Russo, P.; Batista, J.A.; Lovato, M.B. Genetic structure is associated with phenotypic divergence in floral traits and reproductive investment in a high-altitude orchid from the Iron Quadrangle, southeastern Brazil. *PLoS One* **2015**, *10*, e0120645. https://doi.org/10.1371/journal.pone.0120645.

Lima, A.P.; Silva, J.R.S.; Siqueira, G.B.; van den Berg, C. Morphometric analyses as a tool for the delimitation of a species complex of rupicolous *Cattleya* orchids from the Brazilian campos rupestres of Minas Gerais and Bahia. *Phytotaxa* **2024**, *672*, 64–78. https://doi.org/10.11646/phytotaxa.672.1.

Lima, T.M.; Silva, S.F.; Ribeiro, R.V.; Sánchez-Vilas, J.; Pinheiro, F. Short-term salt spray reveals high salt tolerance in a neotropical orchid species. *Theor. Exp. Plant. Physiol*. **2023**, *35*, 355–362. https://doi.org/10.1007/s40626-023-00291-3.

Lima, T.M.; Silva, S.F.; Ribeiro, R.V.; Sánchez-Vilas, J.; Pinheiro, F. Salt tolerance in a neotropical orchid in the absence of local adaptation to salt spray. *Am. J. Bot*. **2024**, *111*, e16373. https://doi.org/10.1002/ajb2.16373.

Lima, T.M.; Silva, S.F.; Sánchez-Vilas, J.; Júnior, W.L.S.; Mayer, J.L.S.; Ribeiro, R.V.; Pinheiro, F. Phenotypic plasticity rather than ecotypic differentiation explains the broad realized niche of a Neotropical orchid species. *Plant Biol*. **2024**, *26*, 989–997. https://doi.org/10.1111/plb.13684.

Luz, A.L.S.; Costa, A.A.S.; Moreira, C.R.; Barberena, F.F.V.A. Vascular epiphytes in the Amazon: main gaps, limitations and perspectives for studies on the subject. *Acta Bot. Bras*. **2023**, *37*, e20220311. https://doi.org/10.1590/1677-941X-ABB-2022-0311.

Luz, A.L.S.; Costa, D.L.L.; Pacheco, J.R.V.; Barberena, F.F.V.A. Orchidaceae in the state of Pará, Brazilian Amazon: an updated checklist reveals underestimated species richness. *Acta Bot. Bras*. **2024**, *38*, e20240011. https://doi.org/10.1590/1677-941X-ABB-2024-0011.

Machnicki-Reis, M.; Engels, M.E.; Petini-Benelli, A.; Smidt, E.C. O gênero *Catasetum* Rich. ex Kunth (Orchidaceae, Catasetinae) no Estado do Paraná, Brasil. *Hoehnea* **2015**, *42*, 185–194. http://dx.doi.org/10.1590/2236-8906-44/2014.

Mancinelli, W.S.; Smidt, E.C. O gênero *Bulbophyllum* (Orchidaceae) na região sul do Brasil. *Rodriguésia* **2012**, *63*, 803–815.

Mancinelli, W.S.; Smidt, E.C. *Sarcoglottis catharinensis* (Orchidaceae): a new species from Brazilian Atlantic Forest. *Kew Bull.* **2013**, *68*, 305–308.

Mancinelli, W.S.; Smidt, E.C. *Homalopetalum joinvillense* (Orchidaceae): a new species from Southern Brazil. *Phytotaxa* **2015a**, *202*, 279–283.

Mancinelli, W.S.; Smidt, E.C. *Homalopetalum leochilus* (Rchb.f.) Soto Arenas (Orchidaceae): complete description and first illustration. *Neodiv.* **2015b**, *8*, 1–7.

Mancinelli, W.S.; Esemann-Quadros, K. Orchidaceae Flora of Joinville, Santa Catarina, Brazil. *Acta Biol. Catarin.* **2016**, *3*, 36–48.

Macagnan, T.A., Smidt, E.C.; Azevedo, C.O.D. A subtribo Cranichidinae Lindl. (Orchidaceae) no Estado do Paraná, Brasil. *Braz. J. Bot.* **2011**, *34*, 447–461.

Martins, A.C., Bochorny, T., Perez-Escobar, O.A., Chomicki, G., Monteiro, S.H.N.; Smidt, E.C. 2018. From tree tops to the ground: Reversals to terrestrial habit in *Galeandra* orchids (Epidendroideae: Catasetinae). *Mol. Phylogenet. Evol.* **2018**, *127*, 952–960.

Martins, P.S. Análise de clines e revisão taxonômica da espécie *Miltonia spectabilis* Ldl. (Orchidaceae – Oncidieae). MSc thesis, Universidade de São Paulo, Piracicaba, 1967.

Martins, P.S. Estudo da variação intra-específica no gênero *Miltonia* Ldl. (Orchidaceae – Oncidieae) Phd dissertation, Universidade de São Paulo,Piracicaba, 1970.

Mattos, J.S.; Pinheiro, F.; Luize, B.G.; Chaves, C.J.N.; Lima, T.M.; Palma-Silva, C.; Leal, B.S.S. The relative role of climate and biotic interactions in shaping the range limits of a neotropical orchid. *J. Biogeogr*. **2023**, *50*, 1315–1328. https://doi.org/10.1111/jbi.14629.

Mauad, A.V.S.R.; Vieira, L.N.; Bolson, M.; Baura, V.A.; Balsanelli, E.; Souza, E.M.; Toscano-de-Brito, A.L.V.; Camargo, E. Complete chloroplast genome of *Anathallis obovata* (Orchidaceae: Pleurothallidinae). *Braz. J. Bot*. **2019**, *42*, 345–352. https://doi.org/10.1007/s40415-019-00524-3.

Mauad, A.V.S.R., Vieira, L.N., Valter, A.B., Balsanelli, E., Souza, E.M., Chase, M.W.; Smidt, E.C. Plastid phylogenomics of Pleurothallidinae (Orchidaceae): Conservative plastomes, new variable markers, and comparative analyses of plastid, nuclear, and mitochondrial data. *PLoS One* **2021**, *16*, e0256126.

Mauad, A.V.S.R., Petini-Benelli, A., Izzo, T.J.; Smidt, E.C. Phylogenetic and molecular dating analyses of *Catasetum* (Orchidaceae) indicate a recent origin and artificial subgeneric groups. *Braz. J. Bot.* **2022**, *3*, 1–13.

Meneguzzo, T.E.C. Mudanças nomenclaturais em Goodyerinae do novo mundo (Orchidaceae). *Orquidário* **2012**, *20*, 86–91.

Meneguzzo, T.E.C. New combinations in Brazilian Orchidaceae. *Neodiv.* **2010**, *5*, 23–25.

Meneguzzo, T.E.C., Bianchetti, L.B.; Proença, C.E. O gênero *Encyclia* (Orchidaceae) no Distrito Federal, Goiás e Tocantins. *Rodriguésia* **2012**, *63*, 277–292.

Meneguzzo, T.E.C.; Baumgratz, J.F.A.; van den Berg, C. Taxonomic studies in the *Aganisia* complex (Orchidaceae, Zygopetalinae). *Phytotaxa* **2015**, *238*, 1–39. https://doi.org/10.11646/phytotaxa.238.1.1.

Meneguzzo, T.E.C. Taxonomic and nomenclatural notes on Zygopetalinae infraspecies (Orchidaceae). *Heringeriana* **2020**, *14*, 157–191. https://doi.org/10.17648/heringeriana.v14i2.917939.

Meneguzzo, T.E.C., Carvalho, B.M., Batista, J.A.N., Gomes, S.M.; Proença, C.B. *Brachystele guayanensis* is a *Cyclopogon* (Orchidaceae): notes on its biology and taxonomy. *Phytotaxa* **2024**, *658*, 109–119.

Meneguzzo, T.E.C., Chase, M.W. An enlarged circumscription of *Bifrenaria* (Orchidaceae: Maxillariiinae). *Phytotaxa* **2024**, *638*, 143–154. https://doi.org/10.11646/phytotaxa.638.2.3.

Meneguzzo, T.E.C., Chase, M.W. An expanded circumscription of *Trichocentrum* (Orchidaceae: Oncidiinae) to include *Grandiphyllum* and *Saundersia*. *Phytotaxa* **2024**, *646*, 82–86. https://doi.org/10.11646/phytotaxa.646.1.7

Meneguzzo, T.E.C.; van den Berg, C. Chimaeras and ghosts: solving a chimaeric specimen and two neglected orchid names. *Willdenowia* **2020**, *50*, 139–146.

Menezes, E.L.F., Giordani, S.C.O., Mendes, J.C.R., Abreu, C.M., Spínola-Filho, P.R.C.; Viotti, J. *Cattleya ninae*, a new species of Orchidaceae (Laeliinae) in the northern Espinhaço region of Minas Gerais, Brazil. *Phytotaxa* **2025**, *693(2)*, 146–156.

Menini-Neto, L., Alves, R.J.V.; Forzza, R.C. A subtribo Pleurothallidinae (Orchidaceae) no Parque Estadual de Ibitipoca, Minas Gerais, Brasil. *Bol. Bot. Univ. São Paulo* **2007**, *25(2)*, 253–278.

Menini-Neto, L.; Docha, A. Redescoberta e tipificação de *Octomeria leptophylla* Barb.Rodr. (Orchidaceae), micro-orquídea endêmica de Minas Gerais, Brasil. *Rodriguésia* **2009**, *60(2)*, 461–465.

Menini-Neto, L., Forzza, R.C.; van den Berg, C. Taxonomic revision of *Pseudolaelia* Porto; Brade (Laeliinae, Orchidaceae). *Acta Bot. Bras.* **2013**, *27*, 418–435.

Menini Neto, L.; van den Berg, C.; Forzza, R.C. Linear and geometric morphometrics as tools to resolve species circumscription in the *Pseudolaelia vellozicola* complex (Orchidaceae, Laeliinae). Pl. *Ecol. Evol*. **2019**, *152*, 53–67. https://doi.org/10.5091/plecevo.2019.1531.

Menini Neto, L.; Forzza, R.C.; van den Berg, C. Taxonomic revision of *Pseudolaelia* Porto; Brade (Laeliinae, Orchidaceae). *Acta Bot. Bras.* **2013**, *27*, 418–435. https://doi.org/10.1590/S0102-33062013000200015.

Menini-Neto, L.; Forzza, R.C. Biogeography and conservation status assessment of *Pseudolaelia* (Orchidaceae). *Bot. J. Linn. Soc*. **2012**, *171*, 191–200. https://doi.org/10.1111/j.1095-8339.2012.01304.x.

Miranda, M.R.; Menini-Neto, L. New Record in the Brazilian Atlantic Forest: *Specklinia aristata* (Orchidaceae: Pleurothallidinae). *Richardiana* **2014**, *14*, 198–204.

Monteiro, S.H.N.; Silva, J.B.F Nova espécie de *Galeandra* Lindl. (Orchidaceae) da Amazônia Brasileira. *Bol. Mus. Para. Emílio Goeldi* **2002**, *18(1)* : 79–84.

Monteiro, S.H.N.; Silva, J.B.F Notas sobre o gênero *Galeandra* Lindl. *Orquidário* **2004**, *18(1)*, 5–9.

Monteiro, S.H.N., Silva, M.F.F.D.; Secco, R.S. O gênero *Galeandra* (Orchidaceae) na Amazônia Brasileira. *Acta Amaz.* **2009**, *39*, 21–33.

Monteiro, S.H.N., Silva, T.C., Santos, L.A.S., Nascimento Junior, J.E.; Prata, A.P.N. Survey of Orchidaceae from the State of Sergipe, Brazil. *Biota Neotrop.* **2012**, *12*, 167–174.

Monteiro, S.H.N., Schnadelbach, A.S., Oliveira, R.P.; van den Berg, C. Molecular Phylogenetics of *Galeandra* (Orchidaceae: Catasetinae) based on Plastid and Nuclear DNA Sequences. *Syst. Bot.* **2010**, *35*, 476–486.

Monteiro, S.H.N., Santos, L.A.S.; Silva, T.C. Expanding the distribution of *Encyclia alboxanthina* Fowlie. *Rev. Bras. Bioci.* **2012**, *10*, 248–250.

Moreira, L.H.L., Soares, R.L.; Barbosa, M.R.D.V. Flora da Mata do Buraquinho, João Pessoa, Paraíba: Orchidaceae. *Rodriguésia* **2020**, *71*, e00362018.

Moreira, L.H.L.; van den Berg, C.; Vasconcelos, M.R. Synopsis of Orchidaceae Juss. in the northern lowland Atlantic Forest. *Acta Bot. Bras.* **2022**, *36*, e20220098.

Mota, R.C., Barros, F.; Stehmann, J.R. Two new species of Orchidaceae from Brazil: *Bulbophyllum carassense* and *Lepanthopsis vellozicola*. *Novon* **2009**, *19(3)*, 380–387.

Nardy, C., Furtado, S.G., Salimena, F.R.G.; Menini-Neto, L. As subtribos Laeliinae e Ponerinae (Epidendroideae, Orchidaceae) no Parque Estadual do Ibitipoca, Minas Gerais, Brasil. *Bol. Bot. Univ. São Paulo* **2016**, *34(1)*, 27–47.

Nazareno, A.G.; Neto, L.M.; Buzatti, R.S.; Berg, C.V.; Forzza, R.C. Four raised to one equals one: A genetic approach to the *Pseudolaelia vellozicola* complex does not follow a math rule. *Ecol. Evol.* **2020**, *10*, 4562–4569. https://doi.org/10.1002/ece3.6148.

Oliveira, M.S., Ferreira, A.W.C., Oliveira, H.C.; Pessoa, E. Orchids of the central region of eastern Maranhão, Brazil. *Rodriguésia* **2020**, *72*, e02582019.

Oliveira, M.S.; Pessoa, E.; Ferreira, A.W.F.; Meneguzzo, T.E.C.; Viana, P.L. Taxonomic revision of *Notylia* Lindl. (Orchidaceae: Oncidiinae) from Brazil. *Phytotaxa* (in press), 2025a.

Pansarin, E.R. Taxonomic notes on Vanilleae (Orchidaceae: Vanilloideae): *Vanilla dietschiana*, a rare south American taxon transferred from *Dictyophyllaria*. *Selbyana* **2010**, *30(2)*, 203–207.

Pansarin, E.R. *Cleistes pusilla* (Orchidaceae): A new species from Central Brazil. *Kew Bull.* 2004, *59(4)*, 555–558.

Pansarin, E.R.; Salatino, A.; Salatino, M.L.F. Phylogeny of South American Pogonieae (Orchidaceae, Vanilloideae) based on sequences of nuclear ribosomal (ITS) and chloroplast (psaB, rbcL rps16, and trnL-F) DNA with emphasis on *Cleistes* and discussion of biogeographic implications. *Org. Divers. Evol.* **2008**, *8*, 171–181. https://doi.org/10.1016/j.ode.2007.09.003.

Pansarin, E.R.; Miranda, M.R. A new species of *Vanilla* (Orchidaceae: Vanilloideae) from Brazil. *Phytotaxa* **2016**, *267(1)*, 84–88.

Pansarin, E.R., Aguiar, J.M.; Ferreira, A.W. A new species of *Vanilla* (Orchidaceae: Vanilloideae) from São Paulo, Brazil. *Brittonia* **2012**, *64*, 157–161.
Pansarin, E.R.; Menezes, E.L.F. A new remarkable *Vanilla* Mill.(Orchidaceae) species endemic to the Espinhaço Range, Brazil: its phylogenetic position and evolutionary relationships among Neotropical congeners. *PhytoKeys* **2023**, *227*, 151.

Pansarin, E.R. Rediscovery and revalidation of the Brazilian endemic *Vanilla schwackeana* Hoehne (Orchidaceae): its distribution and phylogenetic position. *Plant Ecol. Evol.* **2024**, *157*, 32–41. https://doi.org/10.5091/plecevo.110331.

Pansarin, E.R. *Vanilla lindmaniana* and *V. palmarum* (Orchidaceae) are distinct allopatric species. *Plant Ecol. Evol.* **2025a**, *158*, 53–62. https://doi.org/10.5091/plecevo.134103.

Pansarin, L.M., Pansarin, E.R.; Sazima, M. Osmophore structure and phylogeny of *Cirrhaea* (Orchidaceae, Stanhopeinae). Bot. J. Linn. Soc. **2014**, *176(3)*, 369–383.

Pansarin, E.R. Systematics of the *Vanilla chamissonis* complex (Orchidaceae): a study based on integrative taxonomy. *Pl. Ecol. Evol.* **2025**, *158(2)*, 260–278.

Pedron, M.; Buzatto, C.R.; Ramalho, A.J.; Carvalho, B.M.; Radin, J.A.; Singer, R.B.; Batista, J.A.N. Molecular phylogenetics and taxonomic revision of *Habenaria* section *Pentadactylae* (Orchidaceae, Orchidinae). *Bot. J. Linn. Soc.* **2014**, *175*, 47–73. https://doi.org/10.1111/boj.12161.

Perini, V.R.; Leles, B.; Furtado, C.; Prosdocimi, F. Complete chloroplast genome of the orchid *Cattleya crispata* (Orchidaceae:Laeliinae), a Neotropical rupiculous species. *Mitochondrial DNA A DNA Mapp. Seq. Anal.* **2016**, *27(6)*, 4075–4077.

Pessoa, E.M.; Alves, M. Orchidaceae Juss. na Serra de Itabaiana, Sergipe, Brasil. *Rev. Caatinga* **2011**, *24(4)*, 102–114.

Pessoa, E.M.; Alves, M. Flora da Usina São José, Igarassu, Pernambuco: Orchidaceae. *Rodriguésia* **2012**, *63*, 341–356.

Pessoa, E.; Alves, M. *Lockhartia viruensis* (Orchidaceae-Oncidiinae), a new species from Roraima state, Brazilian Amazonia region. *Brittonia* **2012**, *64*, 162–164.

Pessoa, E., Barros, F.; Alves, M. Novelties in Orchidaceae from the Brazilian Amazon. *Check List* **2013**, *9(4)*, 823–825.

Pessoa, E.M., Alves, M.; Silva, I.A.A. Aspects of Orchidaceae distribution in Costa Rica and northwestern South America: a study on similarity with emphasis on the Amazonian Region. *Hoehnea* **2014**, *41*, 623–630.

Pessoa, E.; Alves, M. Orchidaceae em afloramentos rochosos do estado de Pernambuco, Brasil. *Rodriguésia* **2014**, *65*, 717–734.

Pessoa, E.; Alves, M. A new species and a new combination in *Stelis* (Pleurothallidinae-Orchidaceae) from South America. *Brittonia* **2014**, *66*, 156–159.

Pessoa, E., Felix, L.P.; Alves, M. A new *Epidendrum* (Laeliinae-Orchidaceae) from the Atlantic Forest of northeastern Brazil: evidence from morphology and cytogenetics. *Brittonia* **2014**, *66*, 347–352.

Pessoa, E., Barros, F.; Alves, M. *Specklinia integripetala* and *S. ianthina* spp. nov. (Orchidaceae–Pleurothallidinae) from northeastern Brazil. *Nordic J. Bot.* **2014**, *32(2)*. 129–132.

Pessoa, E.M.; Alves, M.; Alves-Araújo, A.; Palma-Silva, C.; Pinheiro, F. Integrating different tools to disentangle species complexes: a case study in *Epidendrum* (Orchidaceae). *Taxon* **2012**, *61*, 721–734. https://doi.org/10.1002/tax.614002.

Pessoa, E.M.; Maciel, J.R.; Alves, M. *Campylocentrum brevifolium* (Lindl.) E.M. Pessoa; M. Alves, a neglected and critically endangered orchid from the Atlantic Forest of Brazil. *Kew Bull.* **2015**, *70*, 43. https://doi.org/10.1007/s12225-015-9594-5.

Pessoa, E.M.; Viruel, J.; Alves, M.; Bogarín, D.; Whitten, W.M.; Chase, M.W. Evolutionary history and systematics of *Campylocentrum* (Orchidaceae: Vandeae: Angraecinae): a phylogenetic and biogeographical approach. *Bot. J. Linn. Soc.* **2018**, *186*, 158–178. https://doi.org/10.1093/botlinnean/box089.

Pessoa, E.M.; Cordeiro, J.M.P.; Felix, L.P.; Almeida, E.M.; Costa, L.; Nepomuceno, A.; Souza, G.; Chase, M.W.; Alves, M.; van den Berg, C. Too many species: morphometrics, molecular phylogenetics and genome structure of a Brazilian species complex in *Epidendrum* (Laeliinae; Orchidaceae) reveal fewer species than previously thought. *Bot. J. Linn. Soc.* **2021**, *195*, 161–188. https://doi.org/10.1093/botlinnean/boaa071.

Pessoa, E.M.; Cordeiro, J.M.P.; Felix, L.P.; Lemes, P.; Viruel, J.; Alves, M.; Chase, M.W.; van den Berg, C. The role of Quaternary glaciations in shaping biogeographic patterns in a recently evolved clade of South American epiphytic orchids. *Bot. J. Linn. Soc.* **2022**, *199*, 252–266. https://doi.org/10.1093/botlinnean/boab039.

Pessoa, E., Valsko, J.J., Vasconcelos, S., Benko-Isepon, A.M.; Alves, M. *Anathallis roseopapillosa* (Orchidaceae - Pleurothallidinae), a new species from the Central Amazon Region. *Syst. Bot.* **2014**, *39*, 1070–1075,

Pessoa, E.; Alves, M.Three new species of *Campylocentrum* (Vandeae, Orchidaceae) from Brazil. *Phytotaxa* **2015**, *217(3)*, 265–272.

Pessoa, E.; Alves, M. Synopsis of Orchidaceae from Serra do Urubu: an area of montane forest, Pernambuco State, Brazil. *Hoehnea* **2015**, *42*, 109–133.

Pessoa, E.; Alves, M. The identity of *Campylocentrum sellowii* (Angraecinae-Orchidaceae): clarifying on its circumscription and lectotypifications. *Feddes Repert.* **2015**, *126(3–4)*, 67–72.

Pessoa, E.; Alves, M. Notes on *Campylocentrum* (Vandeae; Epidendroideae; Orchidaceae): Re-establishment of two South American species. *Phytotaxa* **2015**, *204*. 85–90.

Pessoa, E.; Alves, M.A new *Campylocentrum* (Vandeae; Epidendroideae; Orchidaceae) from submontane Atlantic Forest of northeastern Brazil. *Phytotaxa* **2015,** *197*, 54–58.

Pessoa, E.; Alves, M. A new *Anathallis* (Orchidaceae: Pleurothallidinae) from the Brazilian Amazon. *Phytotaxa* **2015**, *73*, 13–16.

Pessoa, E., Barros, F.; Alves, M. Orchidaceae from Viruá National Park, Roraima, Brazilian Amazon. *Phytotaxa* **2015**, *192*, 61–96.

Pessoa, E.M., Maciel, J.R.; Alves, M.*Campylocentrum brevifolium* (Lindl.) E.M.Pessoa; M.Alves, a neglected and critically endangered orchid from the Atlantic Forest of Brazil. *Kew Bulletin* **2015**, *70*, 42–53.

Pessoa, E, Miranda, M.R.; Alves, M. A new whitish flowered *Epidendrum* (Laeliinae-Orchidaceae) from the Atlantic Forest of southeastern Brazil. *Brittonia* **2016**, *68*, 115–119.

Pessoa, E.; Alves, M. Taxonomical revision of *Campylocentrum* sect. *Dendrophylopsis* Cogn. (Orchidaceae-Vandeae-Angraecinae). *Phytotaxa* **2016**, *286*, 131–152.

Pessoa, E.; Alves, M. Taxonomic Revision of *Campylocentrum* (Orchidaceae-Vandeae-Angraecinae): species with terete leaves. *Syst. Bot.* **2016**, *41*, 700–713.

Pessoa, E.; Alves, M. A new combination in Angraecoides (Angraecinae, Orchidaceae): a forgotten name. *Phytotaxa* **2016**, *269*, 131–132.

Pessoa, E.; Alves, M. Taxonomic revision of *Campylocentrum* sect. *Campylocentrum* Cogn. (Orchidaceae-Vandae-Angraecinae) in Brazil. *Phytotaxa* **2018**, *362(1)*, 1–20.

Pessoa, E.; Alves, M. Taxonomic Revision of *Campylocentrum* sect. *Laevigatum* E.M. Pessoa; M.W. Chase (Orchidaceae-Vandae-Angraecinae). *Syst. Bot.* **2019**, *44*, 115–132.

Pessoa, E.M., Miranda, M.R.; Alves, M. *Campylocentrum benellii* and *C. paludosum* spp. nov. (Angraecinae–Orchidaceae): two new leafless species from Brazil. *Nordic J. Bot.* **2016**, *34(3)*, 376–379. https://doi.org/10.1111/njb.00922.

Pessoa, E.M., Brito, V., Ralf-Neto, F., Costa, W.T., Holanda, G.; Alves, M. Expanding the distribution of *Macroclinium* (Oncidiinae, Orchidaceae) and rediscovery of *M. roseum*. *Brittonia* **2018**, *70*, 1–5.

Pessoa, E.M.; Christenhusz, M.J.M. Molecular phylogenetics provides support for the current circumscription of *Rodriguezia* (Oncidiinae-Orchidaceae) and for a new infrageneric classification of the genus. *Pl. Syst. Evol.* **2024**, *310*, 46.

Pessoa, E.M.; Karremans, A.P. A new *Masdevallia* (Pleurothallidinae: Orchidaceae) from the White-Sand Forests of the Central Amazon, Brazil. *Syst. Bot.* **2022**, *47*, 293–298.

Pessoa, E.M., Engels, M.E.; Alves, M. Novelties in Pleurothallidinae (Orchidaceae) from the Brazilian Amazon. *Phytotaxa* **2018**, *369*, 295–300.

Pessoa, E.M.; Pedrosa, L. A New (Laeliinae-Orchidaceae) from the Extensively Collected Itacolomi peak, Minas Gerais, Brazil. *Syst. Bot.* **2022,** *47*, 938–942.

Pessoa, E.M., Brito, V., Ralf-Neto, F., Fernandes, G., Gomes, G., Fraga, E.; Barros, M.C. Description and phylogenetic relationships of a new, purple-flowered, endangered *Schomburgkia* (Laeliinae; Orchidaceae) from north-eastern Brazil. *Phytotaxa* **2022**, *561*, 151–161.

Pessoa, E.M., Zocal, K.H.; Feitoza, L.H.J.; Rondon-Anjos, M.V.; Pinho, M.F.; Ribeiro, M.G.; Cabral, T.P.S.; Koch, A.K. Typifications in *Epidendrum* species (Laeliinae, Orchidaceae) endemic to Brazil. *Phytotaxa* **2024**, *665(2)*, 167–173. https://doi.org/10.11646/phytotaxa.665.2.7.

Peraza-Flores, L.N.; Carneváli, G.; van den Berg, C. A molecular phylogeny of the *Laelia* alliance (Orchidaceae) and a reassessment of *Laelia* and *Schomburgkia. Taxon* **2016**, *65,* 1249–1262.

Perleberg, T.D., Hentschke, G.S.; Singer, R.B. O gênero *Corymborkis* Thouars (Orchidaceae: Tropidieae) no Rio Grande do Sul, Brasil. *Braz. J. Biosci.* **2008**, *6(4),* 375–379.

Petini-Benelli, A. Orchidaceae da região do Rio Juruena, Mato Grosso, Brasil. *Orquidário* **2014**, *28(1),* 23–31.

Petini-Benelli, A. Novelties in *Catasetum* (Orchidaceae) in the state of Rondônia, Brazil. *Feddes Repert.* **2014**, *125(1/2),* 14–24.

Petini-Benelli, A.; Castro, A.S.F. New record in *Catasetum* (Orchidaceae) for the State of Ceará, Brazil. *Feddes Repert.* **2015**, *126(1/2),* 16–21.

Petini-Benelli, A.; Grade, A. A new species of *Catasetum* (Orchidaceae) for the State of Mato Grosso, Brazil. *Edinburgh J. Bot.* **2008**, *65(1)*, 27–34.

Petini-Benelli, A.; Grade, A. Novo híbrido natural de *Catasetum* LC Rich ex Kunth (Orchidaceae) para o Norte de Mato Grosso, Brasil. *Orquidário* **2010,** *24(4)*, 124–132.

Petini-Benelli, A.; Soares-Lopes, C.R.A. New taxa of *Catasetum* (Orchidaceae, Catasetinae) from Mato Grosso, Brazil. *Richardiana n. ser.* **2017**, *1*: 33–41.

Petini-Benelli, A.; Smidt, E.C. New distribution records of *Catasetum confusum* G.A. Romero-González (Cymbidieae, Epidendroideae, Orchidaceae) from Brazil. *Check List* **2017**, *13,* 2148.

Petini-Benelli, A.; Pessoa, E.M. A new non-Andean South American *Chysis* (Bletiinae-Orchidaceae) with pale flowers. *Phytotaxa* **2019**, *420*, 84–88.

Pinheiro, F.; Barros, F.*Epidendrum puniceoluteum*, uma nova espécie de Orchidaceae do litoral brasileiro. *Hoehnea* **2006**, *33(2)*, 247–250.

Pinheiro, F., Barros, F., Palma-Silva, C., Meyer, D., Fay, M.F., Suzuki, R.M., Lexer, C.; Cozzolino, S. Hybridization and introgression across different ploidy levels in the Neotropical orchids *Epidendrum fulgens* and *E. puniceoluteum* (Orchidaceae). *Mol. Ecol.* **2010**, *19(18)*, 3981–3994.

Pinheiro, L.R., Rabbani, A.R.C., Silva, A.V.C., Silva-Lúdo, A., Pereira, K.L.G.; Diniz, L.E.C. Genetic diversity and population structure in the Brazilian *Cattleya labiata* (Orchidaceae) using RAPD and ISSR markers. *Pl. Syst. Evol.* **2012**, *298*, 1815–1825.

Pinheiro, F.; Koehler, S.; Corrêa, A.M.; Salatino, M.L.F.; Salatino, A.; de Barros, F. Phylogenetic relationships and infrageneric classification of *Epidendrum* subgenus *Amphiglottium* (Laeliinae, Orchidaceae). *Pl. Syst. Evol.* **2009**, *283*, 165–177. https://doi.org/10.1007/s00606-009-0224-2.

Pinheiro, F.; Barros, F.; Palma-Silva, C.; Meyer, D.; Fay, M.F.; Suzuki, R.M.; Lexer, C.; Cozzolino, S. Hybridization and introgression across different ploidy levels in the Neotropical orchids *Epidendrum fulgens* and *E. puniceoluteum* (Orchidaceae). *Mol. Ecol.* **2010**, *19*, 3981–3994. https://doi.org/10.1111/j.1365-294X.2010.04780.x.

Pinheiro, F.; Cozzolino, S. *Epidendrum* (Orchidaceae) as a model system for ecological and evolutionary studies in the Neotropics. *Taxon* **2013**, *62*, 77–88. https://doi.org/10.1002/tax.621007.

Pinheiro, F.; Cozzolino, S.; de Barros, F.; Gouveia, T.M.Z.M.; Suzuki, R.M.; Fay, M.F.; Palma-Silva, C. Phylogeographic structure and outbreeding depression reveal early stages of reproductive isolation in the Neotropical orchid *Epidendrum denticulatum*. *Evolution* **2013**, *67*, 2024–2039.

Pinheiro, F.; Cardoso-Gustavson, P.; Suzuki, R.M.; Abrao, M.C.R.; Guimaraes, L.R.; Draper, D.; Moraes, A.P. Strong postzygotic isolation prevents introgression between two hybridizing Neotropical orchids, *Epidendrum denticulatum* and *E. fulgens*. *Evol. Ecol.* **2015**, *29*, 229–248.

Pinheiro, F.; de Melo e Gouveia, Z.; Manuel, T.; Cozzolino, S.; Cafasso, D.; Cardoso-Gustavson, P.; Suzuki, R.M.; PalmaSilva, C. Strong but permeable barriers to gene exchange between sister species of *Epidendrum*. *Am. J. Bot.* **2016**, *103*, 1472–1482. https://doi.org/10.3732/ajb.1600064.

Pinheiro, F.; Cozzolino, S.; Draper, D.; Barros, F.; Felix, L.P.; Fay, M.F.; Palma-Silva, C. Rock outcrop orchids reveal the genetic connectivity and diversity of inselbergs of northeastern Brazil. *BMC Ecol. Evol.* **2014**, *14*, 49. https://doi.org/10.1186/1471-2148-14-49.

Pinheiro, F.; Dantas-Queiroz, M.V.; Palma-Silva, C. Plant species complexes as models to understand speciation and evolution: a review of South American studies. *Crit. Rev. Plant Sci.* **2018**, *37*, 54–80. https://doi.org/10.1080/07352689.2018.1471565.

Pinheiro, F.; Veiga, G.S.; Chaves, C.J.N.; da Costa Cacossi, T.; da Silva, C.P. Reproductive barriers and genetic differentiation between continental and island populations of *Epidendrum fulgens* (Orchidaceae). *Pl. Syst. Evol.* **2021**, *307*, 2407. https://doi.org/10.1007/s00606-021-01759-y.

Rêgo, H.T.; Azevedo, C.O. Sinopse das Orchidaceae do Parque Nacional de Boa Nova, BA, Brasil. *Hoehnea* **2017**, *44(1)*, 70–89.

Reis, M.M.R.; Smidt, E.C. O gênero *Polystachya* (Orchidaceae, Polystachyinae) no estado do Paraná, Brasil. *Rodriguésia* **2019**, *70*, 1–9. https://doi.org/10.1590/2175-7860201970075.

Reis, M.M.R., Engels, M.E., Petini-Benelli, A.; Smidt, E.C. O gênero *Catasetum* Kunth. (Orchidaceae, Catasetinae) no estado do Paraná. *Hoehnea* **2015**, *42*, 185–194.

Ribeiro, P.L., Borba, E.L.; Toscano-de-Brito, A.L. O gênero *Bulbophyllum* Thouars (Orchidaceae) na Chapada Diamantina, Bahia, Brasil. *Braz. J. Bot.* **2005**, *28*, 423–439.

Ribeiro, P.L.; Borba, E.L.; Smidt, E.C.; Lambert, S.M.; Selbach-Schnadelbach, A.; van den Berg, C. Genetic and morphological variation in the *Bulbophyllum exaltatum* (Orchidaceae) complex occurring in the Brazilian ''Campos rupestres'': implications for taxonomy and biogeography. *Pl. Syst. Evol.* **2008**, *270*, 109–137. https://doi.org/10.1007/s00606-007-0603-5.

Rocha, A.E.S.; Afonso, E.L. *Eltroplectris guimaraesii* (Spiranthinae, Orchidoideae, Orchidaceae), nova espécie para a Amazônia brasileira. *Bol. Mus. Para. Emílio Goeldi* **2022**, *17(2)*, 283–288.

Rodriguez, D.P., Barros, F., Damasceno Junior, G.A.; Bortolotto, I.M. Levantamento da família Orchidaceae no Morro Santa Cruz, Municípios de Corumbá e Ladário, Mato Grosso do Sul, Brasil. *Hoehnea* **2009**, *36*, 613–636.

Rodrigues, V.T., Smidt, E.C.; Barros, F. Revisão taxonômica de *Acianthera* sect. *Pleurobotryae* (Orchidaceae, Pleurothallidinae). *Hoehnea* **2015**, *42*, 615–627.

Rodrigues, V.T. Smidt, E.C., Bolson, M.; Barros, F. Phylogeny of *Acianthera* sect. *Pleurobotryae* (Orchidaceae: Pleurothallidinae), an endemic group of the Atlantic Forest. *Braz. J. Bot.* **2017**, *40*, 1–7.

Rodrigues, J.F.; van den Berg, C.; Abreu, A.G.; Novello, M.; Veasey, E.A.; Oliveira, G.C.; Koehler, S. Species delimitation of *Cattleya coccinea* and *C. mantiqueirae* (Orchidaceae): insights from phylogenetic and population genetics analyses. *Pl. Syst. Evol.* **2015**, *301*, 1345–1359. https://doi.org/10.1007/s00606-014-1156-z.

Rodrigues, J.G.; Borba, E.L. Variation in self-incompatibility and interspecific compatibility in a lineage of the mostly self-compatible genus *Bulbophyllum* (B. *sect. Micranthae*–Orchidaceae). *Pl. Syst. Evol.* **2023**, *309*, 9. https://doi.org/10.1007/s00606-023-01846-2.

Royer, C.A., Toscano de Brito, A.L.V.; Smidt, E.C. *Centroglossa tripollinica* (Barb.Rodr.) Barb.Rodr. (Orchidaceae: Oncidiinae): lectotypification and rediscovery in the State of Paraná, Brazil. *Hoehnea* **2017**, *44*, 139–144.

Royer, C.A., Toscano de Brito, A.L.V.; Smidt, E.C. Nomenclatural notes in the Clade (Oncidiinae: Orchidaceae). *Syst. Bot.* **2024**, *49*, 293–294.

Royer, C.A., Toscano de Brito, A.L.V.; Smidt, E.C. O gênero *Phymatidium* (Orchidaceae: Oncidiinae) no estado do Paraná. **Rodriguésia 2014**, *65*, 251–260.

Royer, C.A., Toscano de Brito, A.L.V.; Smidt, E.C. O gênero *Zygostates* (Orchidaceae: Oncidiinae) no estado do Paraná, Brasil. *Rodriguésia* **2017**, *68*, 1431–1446.

Royer, C.A., Toscano de Brito, A.L.V. Mauad, A.V.S.R.; Smidt, E.C. Phylogenetic position of and (Clade: Oncidiinae: Orchidaceae) based on molecular and morphological data. *Syst. Bot.* **2022**, 47, 927–937.

Salazar, G.A.; van den Berg, C.; Popovkin, A. Phylogenetic relationships of *Discyphus scopulariae* (Orchidaceae, Cranichideae) inferred from plastid and nuclear DNA sequences: evidence supporting recognition of a new subtribe, Discyphinae. *Phytotaxa* **2014**, *173*, 127–139.

Santos, I.S.; Silva, M.J. O gênero *Bulbophyllum* Thouars (Orchidaceae, Epidendroideae) no Parque Nacional da Chapada dos Veadeiros, Goiás, Brasil. *Hoehnea* **2019**, *46*, e872018.

Santos, I.S.; Silva, M.J. *Epidendrum* L. (Orchidaceae, Epidendroideae) no Parque Nacional da Chapada dos Veadeiros, Estado de Goiás, Brasil. *Hoehnea* **2020**, *47*, e202020.

Santos, I.S.; Silva, M.J. O gênero *Galeandra* Lindl. (Orchidaceae, Epidendroideae) no Distrito Federal e no Estado de Goiás, Brasil. *Hoehnea* **2020**, *47*: e462020.

Santos, I.S.; Silva, M.J. New record of *Scaphyglottis livida* (Lindl.) Schltr. (Orchidaceae, Epidendroideae) in Goiás, and a key to *Scaphyglottis* species in the Central-West Region of Brazil. *Check List* **2020**, *16(1)*, 9–15.

Santos, I.S., Alonso, A.A.; Silva, M.J. Uma nova ocorrência de *Bulbophyllum* (Orchidaceae, Epidendroideae) para o estado de Goiás, Brasil. *Rodriguésia* **2020**, *71*, e02262018.

Santos, I.S.; Silva, M.J. *Campylocentrum* Benth. (Orchidaceae, Epidendroideae) no Distrito Federal e no Estado de Goiás, Brasil. *Hoehnea* **2022**, *49*, e762021.

Santos, I.S.; Silva, M.J. Sinopse taxonômica do gênero *Cyrtopodium* R. Br. (Orchidaceae, Epidendroideae) para o município de Niquelândia, incluindo uma nova ocorrência para o Estado de Goiás, Brasil. *Hoehnea* **2023**, *50*, e802021.

Santos, I.S.; Silva, M.J. Novelties in *Brachystele* Schltr. (Orchidaceae, Orchidoideae): a new synonym and a lectotype, including notes on geographic distribution, phenology, relationships and conservation status. *Phytotaxa* **2024**, *642*, 265–272.

Santos, M.C., Toscano de Brito, A.L.V., Amano, E.; Smidt, E.C. Nomenclatural notes in *Anathallis microphyta* (Pleurothallidinae, Orchidaceae). *Phytotaxa* **2018**, *346*, 104–112.

Santos, M.C., Toscano de Brito, A.L.V.; Smidt, E.C. *Anathallis* (Orchidaceae: Pleurothallidinae) no estado do Paraná, Brasil. *Rodriguésia* **2019**, 70: e02722017.

Santos, M.G.; de Azevedo, C.O. New records of Orchidaceae for the Northeast of Brazil. *Paubrasilia* **2022**, *5*, e88–e88.

Santos, T.F., Toscano de Brito, A.L.V.; Smidt, E.C. *Octomeria* (Orchidaceae: Pleurothallidinae) no estado do Paraná, Brasil. *Rodriguésia* **2020**, *71*, e00752018.

Santos, T.F., Toscano de Brito, A.L.V.; Smidt, E.C. A new *Octomeria* (Orchidaceae: Pleurothallidinae) and nomenclatural notes for related Southern Brazilian species. *Phytotaxa* **2022**, *572*, 275–282.

Santos, T.F., Toscano de Brito, A.L.V.; Smidt, E.C. A new (Orchidaceae: Malaxidinae) from the Campos de Altitude of the Atlantic rainforest in southern Brazil. *Nordic J. Bot.* **2023**, *2023(12)*, e04164

Santos, T.F., Coan, A.I.; Smidt, E.C. Revision of *Liparis* (Orchidaceae, Epidendroideae, Malaxidinae) in Brazil. *Rodriguésia* **2023**, *74*, 1–20.

Santos, T.F.; Smidt, E.C. *Malaxis engelsii* (Malaxidinae), a new species from the upper montane forest of the Atlantic Rainforest in Southern Brazil. *Lankesteriana* **2024**, *24*, 61–68.

Silva, M.F.F.; Oliveira, A.T. *Catasetum seccoi*, *Catasetum carrenhianum* e *Catasetum albuquerquei*: novas espécies de Orchidaceae para o Estado do Maranhão, Brasil. *Bol. Mus. Para. Emílio Goeldi* **1999**, *15(2)*, 105–115.

Silva, M.F.F.; Oliveira, A.T.*Catasetum caxarariense*, *Catasetum osakadianum* e *Catasetum alatum*: novas espécies de orchidaceae Juss. para o Estado de Rondônia, Brasil. *Bol. Mus. Para. Emílio Goeldi* **2001**, *17(2)*, 355–366.

Silva, M.F.F.; Silva, J.B.F. Duas novas ocorrências de Orchidaceae para a Flora Brasileira. *Acta Amaz.* **2000**, *30(2)*, 181–186.

Siqueira, C.E.; Zanin, A.; Menini Neto, L. Orchidaceae in Santa Catarina: Update, geographic distribution and conservation. *Check List* **2014**, *10*, 1452–1478. https://doi.org/10.15560/10.6.1452.

Siqueira, C.E., Pessoa, E., Zanin, A.; Alves, M. The smallest Angraecoid species from the Neotropics: a new *Campylocentrum* (Orchidaceae) from a Brazilian subtropical forest. *Syst. Bot.* **2015**, *40*, 79–82.

Smidt, E.C. Orquídeas do Parque Estadual da Ilha Anchieta (PEIA). *Bol. CAOB* **2004**, *53(1)*, 3–9.

Smidt, E.C.; Brito, A.T. A new species of *Sarcoglottis* (Orchidaceae: Spiranthinae), from the Chapada Diamantina, Bahia, Brazil. *Kew Bull.* **2004**, *59(4)*, 569–571.

Smidt, E.C.; Borba, E.L. A new *Bulbophyllum* (Orchidaceae) species in honor of F.C. Hoehne. *Orchids* **2007**, *76*, 934–936.

Smidt, E.C., Silva-Pereira, V., Borba, E.L.; van den Berg, C. Richness, distribution and important areas to preserve *Bulbophyllum* in the Neotropics. *Lankesteriana* **2007**, *7*, 107–113.

Smidt, E.C.; Borba, E.L.A new species of *Bulbophyllum* Thouars (Orchidaceae) from Minas Gerais, Brazil. *Braz. J. Bot.* **2008**, *31*, 453–456.

Smidt, E.C.; Borba, E.L. Two new species of *Bulbophyllum* (Orchidaceae) from Brazil. *Novon* **2009**, *19(1)*, 122–126.

Smidt, E.C.; Borba, E.L. Nomenclatural notes on the Neotropical species of the genus *Bulbophyllum* Thouars (Orchidaceae). *Rodriguésia* **2009**, *60*, 633–639.

Smidt, E.C., Borba, E.L., Gravendeel, B., Fischer, G.A.; van den Berg, C. Molecular phylogeny of the Neotropical sections of *Bulbophyllum* (Orchidaceae) using nuclear and plastid spacers. *Taxon* **2011**, *60*, 1050–1064.

Smidt, E.C., Engels, M.E., Bolson, M.; van den Berg, C. A new species of *Uleiorchis* (Orchidaceae, Gastrodieae) from the Atlantic Forest of Brazil. *Phytotaxa* **2015**, *197*, 257.

Smidt, E.C., Engels, M.E.; Miranda, M.R. A new *Aspidogyne* (Orchidaceae: Goodyerinae) from the Brazilian Atlantic Forest. *Phytotaxa* **2016**, *289(3)*, 279–284.

Smidt, E.C., Toscano de Brito, A.L.V., Martins, A.C., Royer, C.A., Whitten, W.M.; Chase, M.W. Phylogenetics, biogeography and character evolution in the *Ornithocephalus* clade (Orchidaceae, Oncidiinae). *Bot. J. Linn. Soc.* **2018**, *188(4)*, 339–354. https://doi.org/10.1093/botlinnean/boy067.

Smidt, E.C., Toscano de Brito, A.L.V., Mauad, A.V.S.R., Gutiérrez-Morales, N. An expanded concept of *Madisonia* includes *Miscellaneous* species of Pleurothallidinae (Orchidaceae): evidence from molecular analysis. *Phytotaxa* **2021**, *505(1)*, 71–84. https://doi.org/10.11646/phytotaxa.505.1.5.

Smidt, E.C.; Salazar, G.A.; Mauad, A.V.S.R.; Engels, M.E.; Viruel, J.; Clements, M.; Pérez, I.J.; Chase, M.W. An Indomalesian origin in the Miocene for the diphyletic New World jewel orchids (Goodyerinae, Orchidoideae): molecular dating and biogeographic analyses document non-monophyly of the Neotropical genera. *Bot. J. Lin. Soc.* **2021a,** *197,* 322–349. https://doi.org/10.1093/botlinnean/boab028.

Soto-Arenas, M.A.; Salazar, G.A.; van den Berg, C. New combinations in Domingoa, Homalopetalum (Orchidaceae: Laeliinae), and Nemaconia (Orchidaceae: Ponerinae). *Neodiv.* **2007**, *2,* 7–9.

Toscano-de-Brito, A.L.V.; Cribb, P. *Orquídeas da Chapada Diamantina*. Nova Fronteira: São Paulo, Brasil, 2005.

Toscano-de-Brito, A., Felix, L.P.; Dornelas, G.V. *Zygostates aderaldoana*—A New Species in the Ornithocephalus Group of Subtribe Oncidiinae (Orchidaceae) from Paraíba, Northeast Brazil. *Selbyana* **2008**, *29(1)*, 125–127.

Toscano de Brito, A.L.V., Royer, C.A.; Smidt, E.C. Nomenclatural and taxonomic notes in *Phymatidium geiselii* (Oncidiinae, Orchidaceae). *Lankesteriana* **2016**, *16*, 13–19.

Toscano de Brito, A.L.V., Royer, C.A.; Smidt, E.C. Nomenclatural notes in *Miscellaneous* species of *Acianthera* (Pleurothallidinae, Orchidaceae). *Phytotaxa* **2023**, 620, 175–180.

Valsko, J.J., Krahl, A.H., Holanda, A.S.S.; Zartman, C.E. A new species of *Dichaea* (Orchidaceae) for northern Brazil. *Acta Amaz.* **2014**, *44*, 397–401.

Valsko, J.J., Krahl, A.H., Petini-Benelli, A.; Chiron, G.*Catasetum sophiae*, a new species of Orchidaceae (Catasetinae) from northern Brazil. *Phytotaxa* **2019**, *402(2)*, 114–120.

van den Berg, C.; Higgins, W.E.; Dressler, R.L.; Whitten, W.M.; Soto-Arenas, M.A.; Culham, A.; Chase, M.W. A phylogenetic analysis of Laeliinae (Orchidaceae) based on sequence data from internal transcribed spacers (ITS) of nuclear ribosomal DNA. *Lindleyana* **2000**, *15(2)*, 96–114.

van den Berg, C.; Chase, M.W. Nomenclatural notes on Laeliinae-I. *Lindleyana* **2000**, *15(2)*, 115–119.

van den Berg, C.; Chase, M.W. Nomenclatural notes on Laeliinae-II. Additional combinations and notes. *Lindleyana* **2001**, *16(2)*, 109–112.

van den Berg, C.; Chase, M.W. Um novo genero de Laeliinae do Brasil: *Cattleyella* Van den Berg & W. Chase. *Bol. CAOB* **2004**, *52*, 99–101.

van den Berg, C.; Chase, M.W. Nomenclatural Notes on Laeliinae (Orchidaceae): IV. New Combinations in *Laelia* and *Sophronitis. Kew Bull.* **2004**, *59(4)*, 565–567.

van den Berg, C.; Gonçalves, C.N. *Adamantinia*, a showy new genus of Laeliinae from Eastern Brazil. *Orch. Dig.* **2004**, *68*, 230–232.

van den Berg, C.; Azevedo, C.O. Orquídeas. In: *Biodiversidade e Conservação da Chapada Diamantina*, Ministério do Meio Ambiente, Brasília, 2005, pp. 195–208.

van den Berg, C., Smidt, E.C.; Marçal, S. *Leptotes vellozicola*: a new species of Orchidaceae from Bahia, Brazil. *Neodiv.* **2006**, *1(1)*, 1–5.

van den Berg, C.; Higgins, W.E.; Dressler, R.L.; Whitten, W.M.; Soto-Arenas, M.A.; Chase, M.W. A phylogenetic study of Laeliinae (Orchidaceae) based on combined nuclear and plastid DNA sequences. *Ann. Bot.* **2009**, *104*, 417–430. https://doi.org/10.1093/aob/mcp101.

van den Berg, C. Reaching a compromise between conflicting nuclear and plastid phylogenetic trees: a new classification for the genus *Cattleya* (Epidendreae; Epidendroideae; Orchidaceae). *Phytotaxa* **2014**, *186*, 75–86. https://doi.org/10.11646/phytotaxa.186.2.2.

van den Berg, C.; Martins, P.S. Biogeography of Brazilian Cattleyas: Geographic distribution, morphological variability, evolutionary and taxonomic consequences. *Proceedings of the 15th World Orchid Conference*, Rio de Janeiro, 1996.

van den Berg, C. Nomenclatural notes on Laeliinae-III. Notes on *Cattleya* and *Quisqueya*, and a new combination in *Prosthechea. Lindleyana* **2001**, *16(3)*, 142–143.

van den Berg, C. Estudo dos padrões de variabilidade intra e interespecífica em espécies brasileiras de *Cattleya* Lindley (Orchidaceae-Laeliinae), Msc thesis, Universidade Estadual de Campinas, Campinas, 1996.

van den Berg, C. New combinations in the genus *Cattleya* Lindl.(Orchidaceae). *Neodiv.* **2008**, *3*, 3–12.

van den Berg, C. New combinations in the genus *Cattleya* (Orchidaceae). II. Corrections and combinations for hybrid taxa. *Neodiv.* **2010**, *13*, 5–17.

van den Berg, C. Nomenclatural notes on Laeliinae-V. New combinations for invalid names in *Prosthechea* (Orchidaceae). *Phytotaxa* **2015**, *239*, 297–299.

van den Berg, C. Nomenclatural notes on Laeliinae-VI. Further combinations in Cattleya (Orchidaceae). *Neodiv.* **2016**, *9*, 4–5.

van den Berg, C. Nomenclatural notes on Laeliinae-VII. New combinations in *Cattleya* for species and nothospecies originally described in *Hoffmannseggella* (Orchidaceae). *Neodiv.* **2018**, *11*, 1–4.

van den Berg, C. Phylogeny and systematics of *Cattleya* and *Sophronitis*. *Proceedings of the 19th World Orchid Conference*, **2019**, Miami, American Printing Arts, pp. 319–323.

van den Berg, C. Nomenclatural notes on Laeliinae. VIII. Overlooked and new combinations in *Cattleya*, and new infrageneric nothotaxa. *Neodiv.* **2019,** *12*, 1–5.

van den Berg, C.; Menini-Neto, L. The confused taxonomy of *Pseudolaelia irwiniana* (Orchidaceae) with two new synonyms. *Phytotaxa* **2021**, *482*, 297–299.

van den Berg, C. Nomenclatural notes on Laeliinae-IX. New combinations in *Cattleya* and *Laelia* (Orchidaceae, Laeliinae). *Neodiv.* **2022,** *15,* 1–2.

Vencovsky, R (1960) Aplicação de alguns métodos estatísticos à sistemática. PhD thesis, Universidade de São Paulo, Piracicaba, 1960.

Vieira, T.L.; Barros, F. Orchidaceae na Serra do Ouro Branco, Minas Gerais, Brasil. *Rodriguésia* **2017**, *68(2)*, 691–747.

Vieira, T.L., Hall, C.F.; Barros, F. First record of *Triphora* Nutt. (Orchidaceae) for Northeastern Brazil. *Hoehnea* **2017**, *44*, 246–250.

Vieira, T.L.; van den Berg, C. Typification of three names of Brazilian *Prosthechea* (Orchidaceae: Laeliinae). *Phytotaxa* **2020**, *438*, 213–220.

Vieira, T.L., Morais, I.L.; van den Berg, C. Rediscovery of *Prosthechea sessiliflora* (Orchidaceae, Laeliinae) in the Brazilian Cerrado domain provides valuable taxonomic data and allows nomenclatural adjustments. *Phytotaxa* **2023**, *620*, 94–100.

Vieira, T.L., Salazar, G.A.; van den Berg, C. Phylogeny of *Prosthechea* (Laeliinae, Orchidaceae) based on nrITS and plastid DNA sequences: Reassessing the lumper-splitter debate and shedding light on the evolution of this Neotropical genus. *Taxon* **2024**, *73(1)*, 142–160. https://doi.org/10.1002/tax.13124.

Vieira, T.L., Viotti, J.; Menezes, E.L. A new nothospecies of *Prosthechea* (Orchidaceae: Laeliinae) from the Diamantina Plateau, Minas Gerais, Brazil. *Phytotaxa* **2024**, *665(3)*, 282–290.

Wangler, M.S.; Meneguzzo, T.E.C.; van den Berg, C.; Baumgratz, J.F.A. Untangling the type collection and recircunscription of *Pseudolaelia corcovadensis*: a threatened orchid species from Brazilian Atlantic Rain Forest. *Phytotaxa* **2020**, *433*, 265–276.

Zubek, H., Curtivo, L.B., Pessoa, E.M., Caxambu, M.G, Dettke, G., Temponi, L.G.; Silva, S.M. Orchidaceae in Iguaçu National Park, Paraná, Brazil. *Rodriguésia* **2024**, *75*, e01252023. https://doi.org/10.1590/2175-7860202475064.

ANATOMY AND PHYTOCHEMISTRY

Almeida, A.B.R.D., Smidt, E.C.; Amano, E. Development and function of root hairs in *Acianthera* Scheidw. (Orchidaceae: Pleurothallidinae). *Aust. J. Basic Appl. Sci.* **2016**, *10*, 122–126.

Almeida, A.B.R.D., Smidt, E.C.; Amano, E. Evolution of anatomical characters in *Acianthera* section *Pleurobotryae* (Orchidaceae: Pleurothallidinae). *Plos One* **2019**, *14(3)*, e0212677.

Alves, M.F.; Pinheiro, F.; Niedzwiedzki, M.P.; Mayer, J.L.S. First Record of Ategmic Ovules in Orchidaceae Offers New Insights Into Mycoheterotrophic Plants. *Front. Plant Sci.* **2019**, *10*, 1447. https://doi.org/10.3389/fpls.2019.01447.

Alves, M.F.; Pinheiro, P.; Toni, K.L.G. de; Baumgratz, J.F.A. Anatomical Features of Pollinia and Caudicle in *Epidendrum* (Orchidaceae; Epidendroideae). *Bras. J. Bot*. **2024**, *47*, 219–228. https://doi.org/10.1007/s40415-023-00963-z.

Andreota, R.C., Barros, F.; Sajo, M.G. Root and leaf anatomy of some terrestrial representatives of the Cranichideae tribe (Orchidaceae). *Braz. J. Bot.* **2015**, *38*, 367–378.

Andriolli, B.V.; Corredor-Prado, J.P.; Pescador, R.; Montoya-Serrano, F.S.; Vesco, L.L. Dal; Suzuki, R.M. Morpho-Anatomy of in vitro germination and cryopreservation of the orchid *Cattleya crispa* (Orchidaceae). *Rev. Biol. Trop.* **2023**, *71*, e52338. https://doi.org/10.15517/rev.biol.trop..v71i1.52338.

Anjos, J. dos S.; Stefanello, C.A.; Vieira, L. do N.; Polesi, L.G.; Guerra, M.P.; Fraga, H.P. de F. The Cytokinin 6 Benzylaminopurine Improves the Formation and Development of *Dryadella zebrina* (Orchidaceae) In Vitro Shoots. *Bras. J. Bot.* **2021**, *44*, 811–819. https://doi.org/10.1007/s40415-021-00753-5.

Arévalo-Rodrigues, G.; Barros, F.; Davis, A.R.; Cardoso-Gustavson, P. Floral glands in myophilous and sapromyophilous species of Pleurothallidinae (Epidendroideae, Orchidaceae) osmophores, nectaries, and a unique sticky gland. *Protoplasma* **2021**, *258*, 1061–1076. https://doi.org/10.1007/s00709-021-01624-2.

Avi, R.C.; Rodrigues, A.C. Comparative anatomy of the leaf and ramicaul in Pleurothallidinae (Orchidaceae) species. *J. Torrey Bot. Soc.* **2019**, *146(3)*, 198–212.

Barretta-dos-Santos, L.E., Sant'Ana, J., Petini-Benelli, A.; Pedroso-de-Moraes, C. Root anatomy of *Galeandra leptoceras* (Orchidaceae). *Lankesteriana* **2015**, *15(2)*, 159–164.

Bernal, A.A., Smidt, E.C.; Bona, C. Spiral root hairs in Spiranthinae (Cranichideae: Orchidaceae). *Braz. J. Bot.* **2015**, *38*, 411–415.

Blanco, G.D., Hanazaki, N.; Rodrigues, A.C. Anatomical study of Orchidaceae epiphytes species occurring in indigenous territory in the Parque Estadual da Serra do Tabuleiro (PEST), Santa Catarina, Brazil. *Rodriguésia* **2021**, *72*, e02052019. https://doi.org/10.1590/2175-7860202172026.

Bona, C., Engels, M. E., Pieczak, F.S.; Smidt, E.C. Comparative vegetative anatomy of Neotropical Goodyerinae Klotzsch (Orchidaceae Juss.: Orchidoideae Lindl.). *Acta Bot. Bras.* **2020**, *34*, 530–539.

Bonates, L.C.M. Ecophysiological studies of Orchidaceae in Amazonia. II- Ecology of leaf anatomy of species with CAM metabolism in a Central Amazonian white sand campina. *Acta Amaz.* **1993**, *23*, 315–348.

Bonfante, N.O., Smidt, E.C.; Bona, C.Evolution of vegetative morphoanatomical characters in *Pabstiella* (Pleurothallidinae: Orchidaceae). *Flora* **2024**, *317*, 152529. https://doi.org/10.1016/j.flora.2024.152529.

Borba, E.L.; Trigo, J.R.; Semir, J. Variation of diastereoisomeric pyrrolizidine alkaloids in *Pleurothallis* (Orchidaceae). *Biochem. Syst. Ecol.* **2001,** *29*, 45–52. http://dx.doi.org/10.1016/S0305-1978(00)00023-5

Cardoso, P. R. Desenvolvimento floral em espécies de Pleurothallidinae (Orchidaceae) com ênfase nas estruturas secretoras. Phd dissertation, Instituto de Botânica, São Paulo, 2014.

Cardoso-Gustavson, P., Campbell, L.M., Mazzoni-Viveiros, S.C., & de Barros, A.F. Floral colleters in Pleurothallidinae (Epidendroideae: Orchidaceae). *Amer. J. Bot.*, **2014**, *101(4)*, 587–597.

Cardoso-Gustavson, P., Davis, A. R., Bona, C., Campbell, L. M., & De Barros, F. The rostellum, stigma, style and ovarian transmitting tissue in Pleurothallidinae (Orchidaceae: Epidendroideae). *Bot. J. Linn. Soc.* **2017**, *185(3)*, 393–412.

Casique, J.V.; Andrade, E.H.A.; Aguiar-Dias, A.C.A.; Mastroberti, A.A. Novelties in the secretory structures of three species of *Gongora* (Orchidaceae: Stanhopeinae). *Bot. J. Linn. Soc.* **2020**, *10*, 1–20. https://doi.org/10.1093/botlinnean/boaa074.

Casique, J.V.; Silva, E.F.; Andrade, E.H.A.; Mastroberti, A.A.; Aguiar-Dias, A.C.A. Anatomical analyses of floral and extrafloral secreting structures indicate the presence of nectaries and colleters in *Stanhopea grandiflora* Lindl. *Braz. J. Bot.* **2018**, *41*, 725–738. https://doi.org/10.1007/s40415-018-0469-5.

Casique, J.V.; Soares, M.V.B.; Silva, E.F.; Kikuchi, T.Y.; Andrade, E.H.D.A.; Mastroberti, A.A. *Coryanthes macrantha* (Orchidaceae: Stanhopeinae) and their floral and extrafloral secretory structures: an anatomical and phytochemical approach. *Plants* **2022**, *14*, plac039. https://doi.org/10.1093/aobpla/plac039.

Cassola, F.; Nunes, C.E.P.; Lusa, M.G.; Garcia, V.L.; Mayer, J.L.S. Deep in the jelly: Histochemical and functional aspects of mucilage-secreting floral colleters in the orchids *Elleanthus brasiliensis* and *E. crinipes*. *Frontiers in Plant Science* **2019**, *10*, 518. https://doi.org/10.3389/fpls.2019.00518.

Colleta, D., Lopes, R.C.; Silva, I.V. Morfoanatomia foliar de microorquídeas de *Ornithocephalus* Hook. e *Psygmorchis* Dodson; Dressler. *Acta Bot. Bras.* **2008**, *22*, 1068–1076.

Costa, G.V.; Alves, M.F.; Duarte, M.O.; Caetano, A.P.S.; Koehler, S.; Mayer, J.L.S. Apomixis beyond trees in the Brazilian savanna: new insights from the orchid *Zygopetalum mackayi*. AoB *Plants* **2024**, *16*, plae037. https://doi.org/10.1093/aobpla/plae037.

Costa, N. M., Silva, N. A., de Carvalho, R., Saoncella, A. L., de Souza-Leal, T., & Pedroso-de-Moraes, C. Anatomia radicial de *Oncidium flexuosum* Sims. e *Oncidium* Sharry Baby (Orchidaceae). *Nat. Online* **2012**, *10(4)*, 179–182.

Dettke, G.A., Sanches-Marques, Â.M.M.; Milaneze-Gutierre, M.A. Anatomia vegetativa de três espécies de *Maxillaria* Ruiz et Pavón (Orchidaceae). *Rev. Bras. Bioci.* **2007**, *5(S1)*, 531–533.

Dettke, G.A., Sanches-Marques, Â.M.M., Fernandes, M.; Milaneze-Gutierre, M.A. Morfoanatomia dos órgãos vegetativos de *Miltonia regnellii* (Lindl.) Rchb. f. (Oncidiinae, Orchidaceae). *Acta Sci. Biol. Sci.* **2008**, *30(1)*, 9–16.

Donates, L.C.M. Estudos ecofisiológicos de Orchidaceae da Amazônia. II-Anatomia ecológica foliar de espécies com metabolismo CAM de uma campina da Amazônia Central. *Acta Amaz.* **1993**, *23(4)*, 315–348.

Ferreira, W.D.M., Oliveira, S.P.D., Suzuki, R.M., Silva, K.L.F., & Soares Júnior, J.W.P. (2018). Germination, growth and morpho-anatomical development of *Catasetum macrocarpum* (Orchidaceae) in vitro. *Rodriguésia* **2018**, *69*, 2137–2151.

Franco-Pinheiro, A.S., Filho, J.P.L.; Isaias, R.M.S. Structural adaptations of two sympatric epiphytic orchids (Orchidaceae) to a cloudy forest environment in rocky outcrops of Southeast Brazil. *Rev. Biol. Trop.* **2013**, *61(3)*, 1053–1065.

Hengling, M.M.; Gianeti, T.M.R.; Hosomi, S.T.; Machado-Neto, N.B.; Custódio, C.C. Storage of Brazilian *Cattleya* seeds from diverse biomes: lipid composition and effects on germination. *Plant Biosystems - An International Journal Dealing with All Aspects of Pl. Biol.* **2020**, *155*, 487–497. https://doi.org/10.1080/11263504.2020.1762781.

Hosomi, S.T.; Custódio, C.C.; Seaton, P.T.; Marks, T.R.; Machado-Neto, N.B. Improved assessment of viability and germination of *Cattleya* (Orchidaceae) seeds following storage. *In Vitro Cellular; Developmental Biology – Plant* **2012**, *48*, 127–136. https://doi.org/10.1007/s11627-011-9404-1.

Imig, D.C., Junior, J.A.J., Mauad, A.V.S.R., Amano, É.; Smidt, E.C. Vegetative anatomy and its systematic significance in the *Dryadella* Luer (Orchidaceae: Pleurothallidinae). *Feddes Repert.* **2020**, *131(3)*, 175–187.

Joca, T.A.C., de Oliveira, D.C., Zotz, G., Winkler, U., & Moreira, A.S.F.P. The velamen of epiphytic orchids: variation in structure and correlations with nutrient absorption. *Flora*, **2017**, *230*, 66–74.

Kedrovski, H.R.; Sajo, M.G. What are tilosomes? An update and new perspectives. *Acta Bot. Bras.* **2018**, *33*, 106–115.

Kerbauy, G.B.; Estelita, M.E.M. Formation of protocorm-like bodies from sliced root apexes of *Clowesia warscewiczii*. *Revista Brasileira de Fisiologia Vegetal* **1996**, *8*, 157–159.

Krahl, A.H.; Krahl, D.R.P. Anatomia foliar de *Orleanesia yauaperyensis* Barb. Rodr. (Orchidaceae). *Biota Amaz.* **2017**, *7(4)*: 63–65.

Leitão, C.A.E.; Cortelazzo, A.L. Structural and histochemical characterisation of the colleters of *Rodriguezia venusta* (Orchidaceae). *Aust. J. Bot.* **2008**, *56*, 161–165. https://doi.org/10.1071/BT07114.

Leitão, C.A.E.; Cortelazzo, A.L. Structure and histochemistry of the stigmatic and transmitting tissues of *Rodriguezia venusta* (Orchidaceae) during flower development. *Aust. J. Bot.* **2010**, *58*, 233–240. https://doi.org/10.1071/BT09178.

Leite, V.M.C.; Oliveira, P.L. Morfo-anatomia foliar de *Cattleya intermedia* (Orchidaceae). Napea **1987**, *2*, 1–10.

Lima, J. F., Oliveira, D.C., Kuster, V.C.; Moreira, A.S.F.P. Aerial and terrestrial root habits influence the composition of the cell walls of *Vanilla phaeantha* (Orchidaceae). Protoplasma **2024**, *262(1)*, 87–98

Lima, J.F., & Moreira, A.S.F.P. Structural plasticity in roots of the hemiepiphyte *Vanilla phaeantha* Rchb. f.(Orchidaceae): a relationship between environment and function. *Sci. Nat.* **2022**, *109(5)*, 46.

Link, H.F. Elementa philosophiae botanicae. Haude and Spenersche, Berlin, 1824.

Liu, J.W.; Milet-Pinheiro, P.; Gerlach, G.; Ayasse, M.; Nunes, C.E.P.; Alves-dos-Santos, I.; Ramírez, S.R. Macroevolution of floral scent chemistry across radiations of male euglossine bee-pollinated plants. *Evolution* **2024**, *78*, 98–110. https://doi.org/10.1093/evolut/qpad194.

Mayer, J.L.S.; Cardoso-Gustavson, P.; Appezzato-da-Glória, B. Colleters in monocots: new record for Orchidaceae. *Flora* **2011**, *206*, 185–190. https://doi.org/10.1016/j.flora.2010.09.003.

Mayer, J.L.S.; Carmello-Guerreiro, S.M.; Appezzato-da-Glória, B. Anatomical development of the pericarp and seed of *Oncidium flexuosum* Sims (Orchidaceae). *Flora* **2011**, *206*, 601–609. https://doi.org/10.1016/j.flora.2011.01.009.

Mayer, J.L.S.; Scopece, G.; Lumaga, M.R.B.; Coiro, M.; Pinheiro, F.; Cozzolino, S. Ecological and phylogenetic constraints determine the stage of anthetic ovule development in orchids. *Am. J. Bot.* **2021**, *108*, 2405–2415. https://doi.org/10.1002/ajb2.1770.

Mayer, J.L.S.; Stancato, G.C.; Appezzato-da-Glória, B. Direct regeneration of protocorm-like bodies (PLBs) from leaf apices of *Oncidium flexuosum* Sims (Orchidaceae). *PCTOC* **2010**, *103*, 411–416. https://doi.org/10.1007/s11240-010-9782-9.

Melo, M.C., Borba, E.L.; Paiva, E.A.S. Morphological and histological characterization of the osmophores and nectaries of four species of *Acianthera* (Orchidaceae: Pleurothallidinae). *Pl. Syst. Evol.* **2010**, *286*, 141–151. http://dx.doi.org/10.1007/s00606-010-0294-1

Melo, M.C.; Borba, E.L. Morphological variability in rupicolous species of the *Acianthera prolifera* complex (Orchidaceae) occurring in southeastern Brazil. *Pl. Syst. Evol.* **2011**, *293*, 135–145. https://doi.org/10.1007/s00606-011-0435-1.

Moreira, A.S.F.P.; Isaias, R.M.S. Comparative anatomy of the absorption roots of terrestrial and epiphytic orchids. *Braz. Arch. Biol. Tech.* **2008**, *51*, 83–93.

Moreira, A.S.F.P., Lemos Filho, J.P., Zotz, G.; Isaias, R.M.S. Anatomy and photosynthetic parameters of roots and leaves of two shade-adapted orchids, *Dichaea cogniauxiana* Schltr. and *Epidendrum secundum* Jacq. *Flora* **2009**, *204*(8), 604–611.

Moreira, A.S.F.P., Lemos Filho, J.P.; Isaias, R.M.S. Structural adaptations of two sympatric epiphytic orchids (Orchidaceae) to a cloudy forest environment in rocky outcrops of Southeast Brazil. *Rev. Biol. Trop.* **2013**, *61*(3): 1053–1065.

Moreira, D.M., Boff, L., Araújo, G.D.A.C.; Silva, S.M. Ecological inferences in Orchidaceae species from the Brazilian subtropical Atlantic Forest based on morphological and functional anatomical traits. *Flora* **2024**, *317*, 152558.

Moreira, A.S.F.P.; Borba, E.L., Oliveira, D.C.; Isaias, R.M.S.; Ducatti, C.; Lemos-Filho, J.P. Intermediate C 3 -CAM metabolism in *Bulbophyllum involutum*: A species with limited leaf morphological variation in relation to light. *South African Journal of Botany* **2017,** *113*, 40–46. http://dx.doi.org/10.1016/j.sajb.2017.07.015

Moreira, D.M., Boff, L., Araújo, G.D.A.C. & Silva, S.M. Ecological inferences in Orchidaceae species from the Brazilian subtropical Atlantic Forest based on morphological and functional anatomical traits. *Flora* **2024**, *317*, 152558.

Noguera-Savelli, E., & Jáuregui, D. Anatomía foliar comparada y relaciones filogenéticas de 11 especies de Laeliinae con énfasis en Brassavola (Orchidaceae). *Rev. Biol. Trop.* **2011**, *59(3)*, 1047–1059.

Nunes, E.L., Smidt, E.C., Stützel, T.; Coan, A.I. What do floral anatomy and micromorphology tell us about Neotropical *Bulbophyllum* section *Didactyle* (Orchidaceae: Bulbophyllinae)? *Bot. J. Linn. Soc.* **2014**, *175(3)*, 438–452.

Nunes, E.L., Smidt, E.C., Stützel, T., & Ike Coan, A. Comparative floral micromorphology and anatomy of species of *Bulbophyllum* section *Napelli* (Orchidaceae), a Neotropical section widely distributed in forest habitats. *Bot. J. Linn. Soc.* **2015**, *177(3)*, 378-394.

Nunes L.P., E., Emmerich Maldonado, P., C. Smidt, E., Stützel, T., & Ike Coan, A. Floral micromorphology and anatomy and its systematic application to Neotropical *Bulbophyllum* section *Micranthae* (Orchidaceae). *Bot. J. Linn. Soc.* **2017**, *183(2)*, 294–315.

Nunes, C.E., Castro, M. D. M., Galetto, L., & Sazima, M. Anatomy of the floral nectary of ornithophilous *Elleanthus brasiliensis* (Orchidaceae: Sobralieae). *Bot. J. Linn. Soc.* **2013,** *171(4)*, 764–772.

Nunes, C.E.P., Gerlach, G., Bandeira, K. D. O., Gobbo-Neto, L., Pansarin, E. R.; Sazima, M. Two orchids, one scent? Floral volatiles of *Catasetum cernuum* and *Gongora bufonia* suggest convergent evolution to a unique pollination niche. *Flora* **2017**, *232*, 207–216. https://doi.org/10.1016/j.flora.2016.11.016

Oliveira, D.C., Isaias, R.M.S., Ducatti, C.; Lemos-Filho, J.P. Intermediate C3-CAM metabolism in *Bulbophyllum involutum*: A species with limited leaf morphological variation in relation to light. *S. Afr. J. Bot.* **2017**, *113*: 40–46.

Oliveira, M.S., Ferreira, A.W.C.; Oliveira, H.C. Anatomia foliar e radicular comparativa de sete espécies de *Catasetum* Rich. ex Kunth (Orchidaceae: Catasetinae). *Sci. Plena* **2021**, *17(12)*, 1–13.

Oliveira, V.C.; Sajo, M.G. Anatomia foliar de espécies epífitas de Orchidaceae. *Braz. J. Bot.* **1999**, *22*, 365–374.

Oliveira, V.C.; Sajo, M.G. Morfo-anatomia caulinar de nove espécies de Orchidaceae. *Acta Bot. Bras.* **2001**, *15*, 177–188.

Oliveira, V.D.C., & Sajo, M.D.G. Root anatomy of nine Orchidaceae species. *Braz. Arch. Biol. Tech.* **2011** , *42 (4)*, 1–9.

Pansarin, L.M.; de Moraes Castro, M.; Sazima, M. Osmophore and elaiophores of *Grobya amherstiae* (Catasetinae, Orchidaceae) and their relation to pollination. *Bot. J. Linn. Soc.* **2009**, *159(3)*, 408–415.

Pedroso-de-Moraes, C., Leal, T.S., Brescansin, R.L., Benelli, A.P.; Sajo, M.G. Radicular anatomy of twelve representatives of the Catasetinae subtribe (Orchidaceae: Cymbidieae). *An. Acad. Bras. Ciên.* **2012**, *84(2)*, 455–467.

Pedroso-de-Moraes, C., Souza-Leal, T., Barros, F.; Sajo, M.G. Vegetative anatomy of some Brazilian Zygopetalinae (Orchidaceae). *Iheringia* **2018**, *73(2)*, 159–175.

Pena-Passos, M.; Sisti, L.S.; Mayer, J.L.S. Microscopy techniques for interpreting fungal colonization in Mycoheterotrophic plants tissues and symbiotic germination of seeds. *J. Vis. Exp.* **2022**, *183*, e63777. https://doi:10.3791/63777.

Pereira, O.L.; Kasuya, M.C.M.; Rollemberg, C.L.; Chaer, G.M. Isolamento e identificação de fungos micorrízicos rizoctonióides associados a três espécies de orquídeas epífitas neotropicais no Brasil. *Rev. Bras. Cienc. Solo* **2005**, *29*, 191–197. https://doi.org/10.1590/S0100-06832005000200004.

Pereira, M.C.; Pereira, O.L.; Costa, M.D.; Rocha, R.B.; Kasuya, M.C.M. Diversidade de fungos micorrízicos Epulorhiza spp. isolados de *Epidendrum secundum* (Orchidaceae). *Rev. Bras. Cienc. Solo* **2009**, *33*, 1387-1397. https://doi.org/10.1590/S0100-06832009000500012.

Pereira, M.C.; Rocha, D.I.; Veloso, T.G.R.; Pereira, O.l.; Francino, D.M.T.; Meira, R.M.S.A.; Kasuya, M.C.M. Characterization of seed germination and protocorm development of *Cyrtopodium glutiniferum* (Orchidaceae) promoted by mycorrhizal fungi *Epulorhiza* spp. *Acta bot. bras*. 2015, *29*, 567–574. doi: 10.1590/0102-33062015abb0078.

Pessoa, E., Arruda, E., Pereira, F. F. D. S. D.; Alves, M. The odd roots of *Campylocentrum* (Angraeciinae-Orchidaceae): an anatomical study of its morphologically variable roots. *Rodriguésia* **2017**, *68*: 1207–1215.

Piazza, L., Smidt, E.C.; Bona, C. Anatomia comparada dos órgãos vegetativos de espécies de *Bulbophyllum* seção *Didactyle* (Lindl.) Cogn. e *Bulbophyllum* seção *Xiphizusa* Rchb. f. (Orchidaceae). *Hoehnea* **2015**, *42(1)*, 171–183.

Pieczak, F.S., Smidt, E.C., Engels, M.E., Machado, R.G.P.; Bona, C. Floral micromorphology and anatomical diversity in *Microchilus* (Orchidaceae: Goodyerinae). *Flora* **2022**, *290*, 152045.

Picolotto, D.R.N.; Neto, V.B. de P.; Barros, F. de.; Padilha, D.R.C.; Cruz, A.C.F. da.; Otoni, W.C. Micropropagation of *Cyrtopodium paludicolum* (Orchidaceae) from root tip explants. *Crop Crop Breed. Appl. Biotechnol.* **2017**, *17*, 191–197 https://doi.org/10.1590/1984-70332017v17n3a30.

Pires, M.D.F.D.O., Semir, J., Pinna, G.F.D.A.M.D.; Felix, L.P. Taxonomic separation of the genera *Prosthechea* and *Encyclia* (Laeliinae: Orchidaceae) using leaf and root anatomical features. *Bot. J. Linn. Soc.* **2003**, *143(3)*, 293–303.

Reposi, S. D.; Gotelli, M. M.; Torretta, J. P. (2021). Anatomy and ultrastructure floral osmophores of *Catasetum fimbriatum* (Orchidaceae). *Protoplasma* **2021**, *258(5)*, 1091–1102. https://doi.org/10.1007/s00709-021-01625-1

Ribeiro, J.P.O., Paula-Souza, J.D.; Silva, C.J.D. Morfoanatomia de órgãos vegetativos de duas espécies de *Cattleya* (Orchidaceae) nativas do Brasil. *Rodriguésia* **2020**, *71*, e01672017.

Ricci, N.A.P.; Bento, J.P.S.P.; Mayer, J.L.S.; Singer, R.B.; Koehler, S. Gametophytic self-incompatibility in Maxillariinae orchids. *Protoplasma* **2024**, *261*, 271–279 https://doi.org/10.1007/s00709-023-01895-

Rodrigues, A.C., Oliveira, F.M.C., Kedrovski, H.R.; Cruz, R. Within the roots of Pleurothallidinae (Orchidaceae): An evolutionary analysis. *Flora* **2021**, *282*, 151883.

Royer, C.A., Toscano de Brito, A.L.V., Stützel, T., Smidt, E.C.; Nunes, E.L.P. Floral development of the *Ornithocephalus* clade (Oncidiinae, Orchidaceae): the origin of the tabula infrastigmatica, gynostemium appendices and labellar callus. *Bot. J. Linn. Soc.* **2020**, *195(4)*, 636–649. https://doi.org/10.1093/botlinnean/boaa089

Santos, I.S., Alonso, A.A.; Silva, M.J. Anatomia comparada dos órgãos do sistema vegetativo de três espécies de *Encyclia* (Orchidaceae: Epidendroideae). *Iheringia* **2021**, 76, e2021003.

Santos, I.S.; Silva, M.J. Anatomy and histochemistry of the vegetative system of *Brachystele guayanensis* (Lindl.) Schltr. (Orchidaceae), a potential medicinal species. Plants **2023**, *12(14)*, 2635.

Santos, I.S., Melo-de-Pinna, G.F.A.; Silva, M.J. Comparative anatomy of the vegetative systems of eight *Cyrtopodium* R. Br. species (Orchidaceae, Epidendroideae) occurring in Central Brazilian savannas. *Flora* **2023**, *307*, 152384.

Santos, T. F., Amano, E., dos Santos Forstner, A. C., Toscano de Brito, A. L. V., & Smidt, E. D. C. Floral Studies in Octomeria R. BR.(Orchidaceae: Pleurothallidinae). *Feddes Repert.* **2020**, *131(2)*, 101–110.

Saoncella, A.L., Marteline, M.A.; Moraes, C.P. Anatomia dos órgãos vegetativos de *Cattleya violacea* (Kunth) Rolfe (Orchidaceae). *Iheringia* **2017**, *72(1)*, 114–126.

Seixas, F.C., Monteiro, S.H.N., Silva, L.B.; Leite, K.R.B. Leaf anatomy of *Prosthechea moojenii* (Pabst) W. E. Higgins and *P. silvana* Cath.; V. P. Castro (Orchidaceae) as an approach to taxonomy of species occurring in biomes Cerrado and Atlantic Forest of the Bahia. *Amer. J. Pl. Sci.* **2012**, *3*, 1752–1758.

Scatena, V.L.; Nunes, Á.C. Anatomia de "*Pleurothallis rupestris*" Lindl. (Orchidaceae) dos campos rupestres do Brasil. *Bol. Bot. Univ. São Paulo* **1996**, *15*, 35–43.

Silva, C.I.; Milaneze-Gutierre, M.A. Caracterização morfo-anatômica dos órgãos vegetativos de *Cattleya walkeriana* Gardner (Orchidaceae). *Acta Scient.* **2004**, *26(1)*, 91–100.

Silva, F.M.T., Barbosa, W.M.; Ferreira-Júnior, W.G.F. Morphoanatomical characterization of *Brassavola tuberculata* Hook´s (Orchidaceae) leaf and root upon the rocky outcrop. *Rev. Agrogeoamb.* 2021, *13(4):* 671–680.

Silva, I.V., Meira, R.M.S.A., Azevedo, A.A.; Euclydes, R.M.A. Estratégias anatômicas foliares de treze espécies de Orchidaceae ocorrentes em um campo de altitude no Parque Estadual da Serra do Brigadeiro (PESB) – MG, Brasil. *Acta Bot. Bras.* **2006**, *20*, 741–750.

Silva, I.V., Meira, R.M.S.A.; Azevedo, A.A. Anatomia de raízes de espécies de Orchidaceae do Parque Estadual da Serra do Brigadeiro, Minas Gerais. *Hoehnea* **2010**, *37*, 147–161.

Silva, I.V., Oliveira, R.M., Rossi, A.A.B, Silva, A.B., Oliveira, D.M. Use of anatomical root markers for species identification in *Catasetum* (Orchidaceae) at the Portal da Amazônia region, MT, Brazil. *Acta Amaz.* **2015**, *451(1)*, 21–28.

Silva, U.F.; Borba, E.L.; Semir, J.; Marsaioli, A.J. A simple solid injection device for the analyses of *Bulbophyllum* (Orchidaceae) volatiles. *Phytochemistry* **1999**, *50*, 3–34. https://doi.org/10.1016/S0031-9422(98)00459-2.

Silva, P. A., da Silva, A. C. C., de Araújo, H. H., de Castro, R. A., da Costa Ferreira, S., Neto, L. M., & Dias-Pereira, J. Leaf anatomy of five epiphytic *Bulbophyllum* Thouars (Orchidaceae) species from the Cerrado of southeastern Brazil: adaptive strategies and taxonomic value. *Flora* **2025**, *332*, 152831.

Silva Júnior, J. M. D., Rodrigues, M., Castro, E. M. D., Bertolucci, S. K. V., & Pasqual, M. Changes in anatomy and chlorophyll synthesis in orchids propagated in vitro in the presence of urea. Acta Scient. Agron. **2013**, *35*, 65–72.

Sisti, L.S.; Flores-Borges, D.N.A.; de Andrade, S.A.L.; Koehler, S.; Bonatelli, M.L.; Mayer, J.L.S. The role of non-mycorrhizal fungi in germination of the Mycoheterotrophic orchid *Pogoniopsis schenckii* Cogn. *Front. Plant Sci.* **2019**, *29*, 1589. https://doi.org/10.3389/fpls.2019.01589.

Smidt, E.C., Gallo, L.W.; Scatena, V.L. Leaf anatomical and molecular studies in *Bulbophyllum* section *Micranthae* (Orchidaceae) and their implications for systematics. *Braz. J. Bot.* **2013**, *36*, 75–82.

Sousa, K.C.I.; de Araújo, L.G.; Silva, C. S.; Carvalho, J.C.B.; Sibov, S.T.; Gonçalves, L. A.; Pereira, M.C.; Gonçalves, F.J.; Filippi, M.C.C. Seed germination and development of orchid seedlings (*Cyrtopodium saintlegerianum*) with fungi. *Rodriguésia* **2019**, *70*, e02302016 http://dx.doi.org/10.1590/2175-7860201970004.

Soares, J.D.R., Pasqual, M., Araujo, A.G.D., Castro, E.M.D., Pereira, F.J., & Braga, F.T. Leaf anatomy of orchids micropropagated with different silicon concentrations. *Acta Scient. Agron.* **2012**, *34*, 413–421.

Stancato, G.C., Mazzoni-Viveiros, S.C.; Luchi, A.E. Stomatal characteristics in different habitat forms of Brazilian species of *Epidendrum* (Orchidaceae). *Nordic J. Bot.* **1999**, *19(3)*, 271–275.

Teixeira, S.P., Borba, E.L.; Semir, J. Lip anatomy and its implications for the pollination mechanisms of *Bulbophyllum* species (Orchidaceae). *Ann. Bot.* **2004**, *93(5)*, 499–505.

Vasconcelos, F.M.; Andrade, E.H.A.; Teixeira, L.O.A.; Maia, J.G.S. Volatile constituents of floral scents from *Encyclia cordigera* (Kunth) Dressler and *E. randii* (Barb. Rodr.) Porto; Brade (Orchidaceae). *J. Braz. Chem. Soc.* **2022**, *33*, 96–101. https://doi.org/10.21577/0103-5053.20210127.

Vasconcelos, F.M.; Andrade, E.H.A.; Teixeira, L.O.A.; Figueiredo, P.L.B.; Maia, J.G.S. Volatile constituents from *Catasetum* (Orchidaceae) species with occurrence in the Brazilian Amazon. *Plants* **2023**, *12*, 1–22. https://doi.org/10.3390/plants12040703.

Zanenga-Godoy, R.; Costa, C.G. Anatomia foliar de quatro espécies do gênero *Cattleya* Lindl. (Orchidaceae) do Planalto Central Brasileiro. *Acta Bot. Bras.* **2003**, *17*, 101–118.

REPRODUCTIVE BIOLOGY AND POLINATION

Abreu, C.R.M.,; Vieira, M.F. Os beija-flores e seus recursos florais em um fragmento florestal de Viçosa, sudeste brasileiro. Lundiana **2004**, *5(2)*, 129–134.

Aguiar, J.M.R.B.V., Pansarin, L.M., Ackerman, J.D.; Pansarin, E.R. Biotic versus abiotic pollination in *Oeceoclades maculata* (Lindl.) Lindl.(Orchidaceae). *Pl. Sp. Biol.* **2012**, *27(1)*, 86–95.

Aguiar, J.M.R.B.V.; Pansarin, E.R. Deceptive pollination of *Ionopsis utricularioides* (Oncidiinae: Orchidaceae). *Flora* **2019**, *250*, 72–78.

Albuquerque, N.S.L., Milet-Pinheiro, P., Cruz, D.D., Pimentel, G.M., Sousa, A.C., Carneiro, A.M.; Machado, I.C. Phenology, abundance and efficiency of pollinators drive the reproductive success of *Sarcoglottis acaulis* (Orchidaceae) at the Atlantic Forest. *Acta Bot. Bras.* **2022**, *36*, e2021abb0121.

Albuquerque, N.S.L.; Milet-Pinheiro, P.; Cruz, D.D.; Navarro, D.M.A.F.; Machado, I.C. Pollination of the strongly scented *Sarcoglottis acaulis* (Orchidaceae) by male orchid bees: nectar as resource instead of perfume. *Pl. Biol.* **2021,** *23*, 719–727. https://doi.org/10.1111/plb.13297.

Amorim, F. W., Wyatt, G. E.,; Sazima, M. (2014). Low abundance of long-tongued pollinators leads to pollen limitation in four specialized hawkmoth-pollinated plants in the Atlantic Rain forest, Brazil. Naturwissenschaften, 101(11), 893–905. https://doi.org/10.1007/s00114-014-1230-y

Anjos, A.M., Barberena, F.F.V.A.; Pigozzo, C.M. Biologia reprodutiva de *Vanilla bahiana* Hoehne (Orchidaceae). *Orquidário* **2016**, *30*, 67–79.

Barbosa, A.R., Melo, M.C.; Borba, E.L. Self-incompatibility and myophily in *Octomeria* (Orchidaceae, Pleurothallidinae) species. *Pl. Syst. Evol.* **2009**, *283*, 1–8.

Borba, E. L.,; Semir, J. Wind-assisted fly pollination in three Bulbophyllum (Orchidaceae) species occurring in the Brazilian campos rupestres. *Lindleyana* **1998**, *13*, 203–218.

Borba, E.L.; Semir, J. Pollinator specificity and convergence in fly-pollinated *Pleurothallis* (Orchidaceae) species: a multiple population approach. *Ann. Bot.* **2001**, *88(1)*, 75–88.

Borba, E.L., Semir, J.; Shepherd, G.J. Self-incompatibility, inbreeding depression, and crossing potential in five Brazilian *Pleurothallis* (Orchidaceae) species. *Ann. Bot.* **2001**, *88(1)*, 89–99.

Borba, E.L.; Braga, P.I.S. Biologia reprodutiva de *Pseudolaelia corcovadensis* (Orchidaceae): melitofilia e autocompatibilidade em uma Laeliinae basal. *Rev. Bras. Bot.* **2003**, *26(4)*, 541–549.

Borba, E.L., Barbosa, A.R., Melo, M.C., Gontijo, S.L.; Oliveira, H.O. Mating systems in the Pleurothallidinae (Orchidaceae): evolutionary and systematic implications. *Lankesteriana* **2011,** *11*, 207-221.

Borba, E.L.; Felix, J.M.; Solferini, V.N.; Semir, J. Fly-pollinated *Pleurothallis* (Orchidaceae) species have high genetic variability: evidence from isozyme markers. *Amer. J. Bot.* **2001**, *88*, 419–428. https://doi.org/10.2307/2657106.

Borba, E.L.; Shepherd, G J.; Semir, J. Reproductive systems and crossing potential in three species of *Bulbophyllum* (Orchidaceae) occurring in Brazilian 'campo rupestre' vegetation. *Pl. Syst. Evol.* **1999**, *217*, 205–214. https://doi.org/10.1007/BF00984366.

Braga, P.I.S. Estudos da flora orquidológica do Amazonas I - Descrição e observação da biologia floral de *Stanhopea candida* Barb. Rodr. *Acta Amaz.* **1976**, *6*, 433–438. https://doi.org/10.1590/1809-43921976064433.

Braga, P. I. S. Aspectos biológicos das Orchidaceae de uma campina da Amazônia Central. *Acta Amaz.* **1977**, *7(2) (Supl.)*, 1–89.

Brandt, K.; Machado, I.C.; Navarro, D.M.A.F.; Dötterl, S.; Ayasse, M.; Milet-Pinheiro, P. Sexual dimorphism in floral scents of the neotropical orchid *Catasetum arietinum* and its possible ecological and evolutionary significance. *AoB Plants* **2020**, *12*, 4. https://doi.org/10.1093/aobpla/plaa030.

Buzatto, C. R., Nervo, M. H., Sanguinetti, A., van den Berg, C.; Singer, R. B. Efficient pollination and high reproductive success in two Brazilian Spiranthinae orchids: Insights on the evolutionary history of pollination within the Pelexia clade. *Pl. Sp. Biol.* **2022**, *37(2)*, 182–196. https://doi.org/10.1111/1442-1984.12366

Caballero-Villalobos, L.; Silva-Arias, G.A.; Buzatto, C.R.; Nervo, M.H.; Singer, R. B. Generalized food-deceptive pollination in four *Cattleya* (Orchidaceae: Laeliinae) species from Southern Brazil. *Flora* **2017**, *234*, 195–206. https://doi.org/10.1016/j.flora.2017.07.014.

Cabral, P. R. M.,; Pansarin, E. R. Biologia reprodutiva de *Campylocentrum micranthum* (Orchidaceae, Angraecinae). *Rodriguésia*, **2016**, *67(2)*, 379–386. https://doi.org/10.1590/2175-7860201667209.

Cardoso, J. C. F., Johnson, S. D., Rezende, U. C.,; Oliveira, P. E. The lady's "slippery" orchid: functions of the floral trap and aphid mimicry in a hoverfly-pollinated *Phragmipedium* species in Brazil. *Ann. Bot.*, **2023**, *131(2)*, 275–286. https://doi.org/10.1093/aob/mcac140

Cardoso-Gustavson, P., Saka, M.N., Pessoa, E.M., Palma-Silva, C.; Pinheiro, F. Unidirectional transitions in nectar gain and loss suggest food deception is a stable evolutionary strategy in *Epidendrum* (Orchidaceae): insights from anatomical and molecular evidence. *BMC Pl. Biol.* **2018**, *18*, 179. https://doi.org/10.1186/s12870-018-1398-y.

Carvalho, R.; Machado, I.C. Pollination of *Catasetum macrocarpum* (Orchidaceae) by *Eulaema bombiformis* (Euglossini). *Lindleyana* **2002**, *17*, 85–90.

Carvalho, R.; Machado, I.C. *Rodriguezia bahiensis* Rchb. f.: biologia floral, polinizadores e primeiro registro de polinização por moscas Acroceridae em Orchidaceae. *Braz. J. Bot.* **2006**, *29*, 461–470.

Castro, J. B., Perdomo, O.; Singer, R. B.. Pollination biology and reproductive success in four Brazilian species of Gomesa (Orchidaceae: Oncidiinae): Specific pollinators, but high pollen loss and low fruit set. *Pl. Sp. Biol.* **2022**, *37(1)*, 132–147. https://doi.org/10.1111/1442-1984.12361

Clemente, M. A., Lange, D., Del-Claro, K., Prezoto, F., Campos, N. R.,; Barbosa, B. C. (2012). Flower-visiting social wasps and plants interaction: Network pattern and environmental complexity. *Psyche* **2012**, *2012*, 78431 https://doi.org/10.1155/2012/478431

Coleta, MHD; Stort, MNS (1976) Estudo da possível influência do comprimento do órgão reprodutor feminino como barreira de isolamento reprodutivo em orquídeas. *Cien. Cult.* **1976**, *28(8)*, 936–939.

Custodio, C.C.; Machado-Neto, N.B.; Singer, R.B.; et al. Storage of orchid pollinia with varying lipid thermal fingerprints. *Protoplasma* **2020**, *257*, 1401–1413. https://doi.org/10.1007/s00709-020-01514-z.

Davies, K. L., Pansarin, E. R.,; Stpiczyńska, M. Labellar structure of the *Maxillaria splendens* alliance (Orchidaceae: Maxillariinae) indicates floral polyphenols as a reward for stingless bees. *Plants* **2023**, *12(4)*, 921. https://doi.org/10.3390/plants12040921

Duarte, M.O.; Oliveira, D.M.T.; Borba, E.L. Ontogenesis of ovary and fruit of *Acianthera johannensis* (Pleurothallidinae, Orchidaceae) reveals a particular female embryology. *Flora* **2019**, *259*, 151462. https://doi.org/10.1016/j.flora.2019.151462.

Duarte, M.O.; Oliveira, D.M.T.; Borba, E.L. Two self-incompatibility sites occur simultaneously in the same *Acianthera* species (Orchidaceae, Pleurothallidinae). *Plants* **2020**, *9*, 1758. https://doi.org/10.3390/plants9121758.

Ferreira, N. P., Chiavelli, L. U. R., Savaris, C. R., Oliveira, S. M., Lucca, D. L., Milaneze-Gutierre, M. A., Faria, R. T.,; Pomini, A. M.. Chemical study of the flowers of the orchid *Oncidium baueri* Lindley and their visiting bees *Trigona spinipes* Fabricius. *Biochem. Syst. Ecol.* **2019**, 86, 103918. https://doi.org/10.1016/j.bse.2019.103918

Hall, C. F., Gomes-Klein, V. L.,; Barros, F. . Biologia floral e reprodutiva de *Cyrtopodium eugenii* Rchb.f. & Warm. (Orchidaceae). Anais Do 6-o Congresso Nacional de Botânica, 2009.

Hunhoff, V. L., Silva, C. A., Lage, L. A., Krause, W. K.,; Palu, E. G. Biologia, morfologia floral e potencial ornamental de Cyrtopodium saintlegerianum (Orchidaceae). *Revista Agro@Mbiente On-Line* **2017**, *10*(4), 358. https://doi.org/10.18227/1982-8470ragro.v10i4.3925

Krahl, A. H., Krahl, D. R. P., Valsko, J. J., Webber, A. C.,; Pansarin, E. R. Evidence of reward production and pollination by Centris in Encyclia (Orchidaceae: Laeliinae): the reproductive biology of *Encyclia mapuerae*. *Aust. J. Bot.* **2017**, *65(3)*, 225–232. https://doi.org/10.1071/BT16253

Maciel, A. A., Cardoso, J. C. F.; Oliveira, P. E. On the low reproductive success of two Cyrtopodium species (Orchidaceae: Cyrtopodiinae): The relative roles of biotic and abiotic pollination. *Pl. Sp. Biol.* **2020**, *35(1)*, 49–58. https://doi.org/10.1111/1442-1984.12260

Martini, P., Schlindwein, C.; Montenegro, A. Pollination, flower longevity, and reproductive biology of *Gongora quinquenervis* Ruíz and Pavón (Orchidaceae) in an Atlantic forest fragment of Pernambuco, Brazil. *Pl. Biol.* **2003**, *5(5)*, 495–503.

Matias, L. Q., Braga, P. I. S.,; Freire, A. Biologia reprodutiva de *Constantia cipoensis* Porto & Brade (Orchidaceae) endêmica da Serra do Cipó, Minas Gerais. *Rev. Bras. Bot.* **1996**, *19(1)*, 119–125

Melo, M.C., Taucce, P.P.G.; Borba, E.L. Reproductive biology and isolation mechanisms in rupicolous species of the *Acianthera prolifera* complex (Orchidaceae) occurring in southeastern Brazil. *Pl. Syst. Evol.* **2011**, *293*, 161–176.

Mickeliunas, L., Pansarin, E. R.,; Sazima, M. Biologia floral, melitofilia e influência de besouros Curculionidae no sucesso reprodutivo de *Grobya amherstiae* Lindl. (Orchidaceae: Cyrtopodiinae). *Rev. Bras. Bot.* **2006**, *29(2)*, 251–258. https://doi.org/10.1590/S0100-84042006000200006

Milet-Pinheiro, P.; Gerlach, G. Biology of the Neotropical orchid genus *Catasetum*: A historical review on floral scent chemistry and pollinators. *Perspect. Plant Ecol. Evol. Syst.* **2017**, *27*, 23–34. https://doi.org/10.1016/j.ppees.2017.05.004.

Milet-Pinheiro, P.; Navarro, D.M.A.F.; Dötterl, S.; Carvalho, A.T.; Pinto, C.E.; Ayasse, M.; Schlindwein, C. Pollination biology in the dioecious orchid *Catasetum uncatum*: How does floral scent influence the behaviour of pollinators? *Phytochem.* **2015**, *116*, 149–161. https://doi.org/10.1016/j.phytochem.2015.02.027.

Milet-Pinheiro, P.; Silva, J.B.F.; Navarro, D.M.A.F.; Machado, I.C.S.; Gerlach, G. Notes on pollination ecology and floral scent chemistry of the rare neotropical orchid *Catasetum galeritum* Rchb.f. *Pl. Sp. Biol.* **2018**, *33*, 158–163. https://doi.org/10.1111/1442-1984.12202.

Nunes, C.E.P.; Amorim, F.W.; Mayer, J.L.S.; Sazima, M. (2015). Pollination ecology of two species of Elleanthus (Orchidaceae): novel mechanisms and underlying adaptations to hummingbird pollination. *Pl. Biol*. **2015**, *18*, 15–25. https://doi.org/10.1111/plb.12312.

Nunes, C.E.P.; Gerlach, G.; Bandeira, K.D.O.; Gobbo-Neto, L.; Pansarin, E.R.; Sazima, M. Intriguing chemical similarity: floral scents of *Catasetum cernuum* and *Gongora bufonia* suggest convergent evolution to a unique pollination niche. *Flora* **2017**, *232*, 207–216. https://doi.org/10.1016/j.flora.2016.11.016.

Nunes, C. E. P., Peñaflor, M. F. G. V, Bento, J. M. S., Salvador, M. J.,; Sazima, M.. The dilemma of being a fragrant flower: the major floral volatile attracts pollinators and florivores in the euglossine-pollinated orchid Dichaea pendula. *Oecologia* **2016**, *182(4)*, 933–946. https://doi.org/10.1007/s00442-016-3703-5

Nunes, C. E. P., Wolowski, M., Pansarin, E. R., Gerlach, G., Aximoff, I., Vereecken, N. J., Salvador, M. J.,; Sazima, M. More than euglossines: the diverse pollinators and floral scents of Zygopetalinae orchids. *Sci. Nat.* **2017**, *104(11–12)*, 92. https://doi.org/10.1007/S00114-017-1511-3

Oliveira, M.S.; Oliveira, M.S.; Nascimento, L.D.; Pessoa, E.; Aguiar, A.E.H.; Viana, P.L. First comprehensive report on the chemical composition of the floral perfume of *Notylia* (Orchidaceae). *Nat. Prod. Res.* **2025**, *1*, 1–7. https://doi.org/10.1080/14786419.2024.2448851.

Oliveira, R. T., Silva Oliveira, J. P.,; Macedo, A. F. *Vanilla* beyond *Vanilla planifolia* and *Vanilla ×tahitensis*: taxonomy and historical notes, reproductive biology, and metabolites. *Plants* **2022**, *11(23)*, 3311. https://doi.org/10.3390/plants11233311

Paiva, V.B., Correa, A.P.M., de Barros, F., Padilha, D.R.C.; Borges, M.C.R.Z. The *Bletia catenulata* ornamental orchid is self-compatible but pollinator-dependent for reproduction. *Pesq. Agrop. Trop.* 2015, *45(4)*, 473–479. https://doi.org/10.1590/1983-40632015v4538410

Pansarin, L.M., Pansarin, E.R.; Sazima, M. Reproductive biology of *Cyrtopodium polyphyllum* (Orchidaceae): a Cyrtopodiinae pollinated by deceit. *Pl. Biol.* **2008**, *10(5)*, 650–659.

Pansarin, L.M., Castro, M.C.; Sazima, M.Osmophore and elaiophores of *Grobya amherstiae* (Catasetinae, Orchidaceae) and their relation to pollination. *Bot. J. Linn. Soc.* **2009**, *159(3)*, 408–415.

Pansarin, E.R.; Amaral, M.C.E. Reproductive biology and pollination of southeastern Brazilian *Stanhopea* Frost ex Hook. (Orchidaceae). *Flora* **2009**, *204*, 238–249. https://doi.org/10.1016/j.flora.2008.01.014

Pansarin, E.R.,; Estanislau do Amaral, M. do C. Pollen and nectar as a reward in the basal epidendroid *Psilochilus modestus* (Orchidaceae: Triphoreae): A study of floral morphology, reproductive biology and pollination strategy. *Flora* **2008**, *203(6)*, 474–483. https://doi.org/10.1016/j.flora.2007.07.004

Pansarin, E.R.,; Ferreira, A.W.C. Butterfly pollination in *Pteroglossa* (Orchidaceae, Orchidoideae): a comparative study on the reproductive biology of two species of a Neotropical genus of Spiranthinae. J. Pl. Res. **2015**, *128(3)*, 459–468. https://doi.org/10.1007/s10265-015-0707-x

Pansarin, E.R. Biologia floral de *Cleistes macrantha* (Barb. Rodr.) Schltr. (Orchidaceae: Vanilloideae: Pogoniinae). *Braz. J. Bot.* **2003**, *26*, 73–80.

Pansarin, E.R.; Amaral, M.C.E. Reproductive biology and pollination mechanisms of *Epidendrum secundum* (Orchidaceae). Floral variation: a consequence of natural hybridization? *Pl. Biol.* **2008**, *10(2)*, 211–219.

Pansarin, E.R.; Pansarin, L.M. Reproductive biology of *Epidendrum tridactylum* (Orchidaceae: Epidendroideae): a reward-producing species and its deceptive flowers. Pl. Syst. Evol. **2014**, *300*, 321–328.

Pansarin, E.R. Recent advances on evolution of pollination systems and reproductive biology of Vanilloideae (Orchidaceae). *Lankesteriana* **2016**, *16*, 255–267. https://doi.org/10.15517/lank.v16i2.26010.

Pansarin, E.R. *Vanilla* flowers: Much more than food-deception. *Bot. J. Linn. Soc.* **2022**, *198*, 57–73. https://doi.org/10.1093/botlinnean/boab046.

Pansarin, E.R., Bittrich, V.; Amaral, M.C.E. At daybreak - Reproductive biology and isolating mechanisms of *Cirrhaea dependens* (Orchidaceae). *Pl. Biol.* **2006**, *8(4)*, 494–502. https://doi.org/10.1055/s-2006-923800

Pansarin, E.R.,; Pansarin, L.M.. Reproductive biology of *Trichocentrum pumilum*: An orchid pollinated by oil-collecting bees. *Pl. Biol.* **2011**, *13(4)*, 576–581. https://doi.org/10.1111/j.1438-8677.2010.00420.x

Pansarin, E. R. Nectar-mediated avian pollination in *Cattleya* (Orchidaceae: Laeliinae). Pl. Biol. **2024,** *26(2)*, 181–187. https://doi.org/10.1111/plb.13606

Pansarin, E.R.; Amaral, M.C.E. Reproductive biology and pollination of southeastern Brazilian Stanhopea Frost ex Hook. (Orchidaceae). *Flora* **2009**, *204(3)*, 238–249. https://doi.org/10.1016/j.flora.2008.01.014

Pansarin, E.R.,; Amaral, M.C.E. Biologia reprodutiva e polinização de duas espécies de *Polystachya* Hook. no Sudeste do Brasil: Evidência de pseudocleistogamia em Polystachyeae (Orchidaceae). *Rev. Bras. Bot.* **2006**, *29(3)*, 423–432. https://doi.org/10.1590/S0100-84042006000300009

Pansarin, L.M.; Pansarin, E.R.; Gerlach, G.; Sazima, M. The natural history of *Cirrhaea* and the pollination system of Stanhopeinae (Orchidaceae). *Int. J. Pl. Sci.* **2018**, *179*, 436–449. https://doi.org/10.1086/697997.

Pedron, M., Buzatto, C. R., Singer, R. B., Batista, J. A. N.; Moser, A. Pollination biology of four sympatric species of Habenaria (Orchidaceae: Orchidinae) from southern Brazil. *Bot. J. Linn. Soc.* **2012**, *170(2)*, 141–156. https://doi.org/10.1111/j.1095-8339.2012.01285.x

Rech, A.R., Rosa, Y.B.C.J.; Manente-Balestieri, F.C.L. Aspects of the reproductive biology of Brassavola cebolleta Rchb.f. (Orchidaceae). *Acta Sci. Biol. Sci.* **2010**, *32(4)*, 335–341. https://doi.org/10.4025/actascibiolsci.v32i4.7148

Rocha-Filho, L. C., Krug, C., Silva, C. I.,; Garófalo, C. A. Floral resources used by Euglossini bees (Hymenoptera: Apidae) in coastal ecosystems of the Atlantic Forest. *Psyche* 2012, 1–13. https://doi.org/10.1155/2012/934951

Sanguinetti, A., Buzatto, C.R., Pedron, M., Davies, K.L., Ferreira, P.M.A., Maldonado, S.; Singer, R.B. Floral features, pollination biology and breeding system of *Chloraea membranacea* Lindl. (Orchidaceae: Chloraeinae). *Ann. Bot.* **2012**, *110(8)*, 1607–1621.

Santos, T.F., Amano, E., Forstner, A.C.S., Toscano de Brito, A.L.V.; Smidt, E.C. Floral studies in *Octomeria* R. BR. (Orchidaceae: Pleurothallidinae). *Feddes Repert.* **2020**, *131(2)*, 101-110.

Sazima, M. Polinização por moscas em *Bulbophyllum warmingianum* Cogn. (Orchidaceae), na Serra do Cipó, Minas Gerais. *Braz. J. Bot.* **1978**, *1*, 133–138.

Silva-Pereira, V., Smidt, E.C.; Borba, E.L. Isolation mechanisms between two sympatric *Sophronitis* (Orchidaceae) species endemic to Northeastern Brazil. *Pl. Syst. Evol.* **2007,** *269*, 171–182.

Silveira, R. S., Singer, R. B.,; Ferro, V. G.. Pollination in *Epidendrum densiflorum* Hook. (Orchidaceae: Laeliinae): fraudulent trap-flowers, self-incompatibility, and a possible new type of mimicry. *Plants* **2023**, *12(3)*, 679. https://doi.org/10.3390/plants12030679

Singer, R.B.; Koehler, S. Notes on the pollination biology of *Notylia nemorosa* (Orchidaceae): do pollinators necessarily promote cross pollination?. *J. Pl. Res.* **2003**, *116*, 19–25.

Singer, R.B.; Cocucci, A.A. Pollination mechanism in southern Brazilian orchids which are exclusively or mainly pollinated by halictid bees. *Pl. Syst. Evol.* **1999**, *217*, 101–117. https://doi.org/10.1007/BF00984924.

Singer, R.B.; Sazima, M. The pollination mechanism of three sympatric *Prescottia* (Orchidaceae: Prescottinae) species in southeastern Brazil. *Ann. Bot.* **2001**, *88*, 999–1005. https://doi.org/10.1006/anbo.2001.1535.

Singer, R. B.; Koehler, S. Pollinarium morphology and floral rewards in Brazilian Maxillariinae (Orchidaceae). *Ann. Bot.* 2004, *93(1)*, 39–51. https://doi.org/10.1093/aob/mch009

Singer, R.B.; Marsaioli, A.J.; Flach, A.; Marsaioli, A.J.; Reis, M.G. *The Ecology and Chemistry of Pollination in Brazilian Orchids: Recent Advances*. Global Science Books, **2006**, 4(1859), 569–582

Singer, R.B.; Buzatto, C.R.; Sanguinetti, A.; Nervo, M.H. Found again: the extremely rare *Codonorchis canisioi* (Orchidaceae: Codonorchideae) reappears after being missing for 78 years. *Pl. Syst. Evol.* **2018**, *304*, 1157–1663. https://doi.org/10.1007/s00606-018-1538-8.

Smidt, E.C., Silva-Pereira, V.; Borba, E.L. Reproductive biology of two *Cattleya* (Orchidaceae) species endemic to north-eastern Brazil. *Pl. Sp. Biol.* **2006**, *21*, 85–91.

Stort, M.N.S. Influência do número de polínias sobre a fertilidade em cruzamentos de orquídeas, *Cien. Cult.* **1969**, *21(2)*, 238–239.

Stort, M.N.S. Estudos em híbridos F1 artificiais de orquídeas (Orchidaceae) com vistas à esterilidade. PhD dissertation, University of São Paulo, Piracicaba, 1970.

Stort, M.N.S. Estudos em híbridos F1 artificiais de orquídeas. *Cien. Cult.* **1972**, *24(9)*, 847–851.

Stort, M.N.S. Desarrollo del óvulo, posterior a la polinización en *Cyrtopodium cardiochilum* Lindl. *Orquideología* **1972,** *7(1)*, 22–28.

Stort, M.N.S. Ovule development after pollination in *Eulophidium* orchids. *Amer. Orch. Soc. Bull.* **1972**, *41*, 23–28.

Stort, M.N.S. Cruzamentos artificiais entre plantas diplóides e tetraplóides de *Cattleya bicolor* Lindl. *Cien. Cult.* **1976,** *28(10)*, 1208–1211.

Stort, M.N.S. Autopolinização e polinização cruzada em algumas espécies do gênero *Cattleya* (Orchidaceae). *Cien. Cult.* **1979,** *32(8)*, 1080–1083.

Stort, M.N.S. Sterility barriers of some artificial F1 orchid hybrids: male sterility. I. Microsporogenesis and pollen germination. *Amer. J. Bot.* **1984**, *71(3)*, 309–318.

Stort, M.N.S. Fertilidade de cruzamentos e relação filogenética entre algumas espécies do gênero *Cattleya* Lindl. (Orchidaceae). *Rev. Bras. Bot.* **1986,** *9*, 69–74.

Storti, E.F., Braga, P.I.S.; Storti-Filho, A. Biologia reprodutiva de *Cattleya eldorado*, uma espécie de Orchidaceae das campinas amazônicas. *Acta Amaz.* **2011**, *41*, 361–368.

Verola, C. F. Biologia floral e sistemas de reprodução em espécies de Bulbophyllum (Orchidaceae) ocorrentes em mata de galeria, campo rupestre e floresta estacional. PhD dissertation, Universidade Estadual de Campinas, 2002.

CYTOGENETICS

Assis, F.N.M.; Souza, B.C.Q.; Medeiros-Neto, E.; Pinheiro, F.; Silva, A.E.B.; Felix, L.P. Karyology of the Genus *Epidendrum* (Orchidaceae: Laeliinae) with Emphasis on Subgenus *Amphiglottium* and Chromosome Number Variability in *Epidendrum secundum*. *Bot. J. Linn. Soc.* **2013**, *172*(3), 329–344. https://doi.org/10.1111/boj.12045.

Cabral, J.S.; Felix, L.P.; Guerra, M. Heterochromatin diversity and its co-localization with 5S and 45S rDNA sites in chromosomes of four *Maxillaria* species (Orchidaceae). *Genet. Mol. Biol.* **2006**, *29*, 659–664. https://doi.org/10.1590/S1415-47572006000400015.

Cordeiro, J.M.; Chase, M.W.; Hágsater, E.; Almeida, E.M.; Costa, L.; Souza, G.; Felix, L.P. Chromosome number, heterochromatin, and genome size support recent polyploid origin of the *Epidendrum nocturnum* group and reveal a new species (Laeliinae, Orchidaceae). *Botany* 2022, *100*, 409–421. https://doi.org/10.1139/cjb-2021-0113

Félix, L.P.; Guerra, M. Cytogenetics and cytotaxonomy of some Brazilian species of Cymbidioid orchids. *Genet. Mol. Biol.* **2000**, *23*, 957–978.

Felix, L.P.; Guerra, M. Basic chromosome numbers of terrestrial orchids. *Pl. Syst. Evol.* **2005**, *254*, 131–148.

Felix, L.P.; Guerra, M. Cytogenetic studies on species of *Habenaria* (Orchidoideae: Orchidaceae) occurring in the northeast of Brazil. *Lindleyana* **1998**, *13*, 224–230. https://doi.org/10.5555/19991602897.

Felix, L.P.; Guerra, M. Chromosome analysis in *Psygmorchis pusilla* (L.) Dodson; Dressier: the smallest chromosome number known in Orchidaceae. *Caryologia* **1999**, *52*, 165–168. https://doi.org/10.1080/00087114.1998.10589169.

Felix, L.P.; Guerra, M. Variation in chromosome number and the basic number of subfamily Epidendroideae (Orchidaceae). *Bot. J. Linn. Soc.* **2010**, *163*, 234–278. https://doi.org/10.1111/j.1095-8339.2010.01059.x

Meirmans, P.G.; Liu, S.; van Tienderen, P.H. The analysis of polyploid genetic data. *J Hered*. **2018**, *109*, 283–296. https://doi.org/10.1093/jhered/esy006.

Medeiros-Neto, E., Nollet, F., Moraes, A.P.; Felix, L.P. Intrachromosomal karyotype asymmetry in Orchidaceae. *Genet. Mol. Biol.* **2017**, *40(3)*, 610–619.

Moraes, A.P.; Leitch, I.J.; Leitch, A.R. Chromosome studies in Orchidaceae: Karyotype divergence in Neotropical genera in subtribe Maxillariinae. *Bot. J. Linn. Soc.* **2012**, *170*, 29–39. https://doi.org/10.1111/j.1095-8339.2012.01266.x.

Moraes, A.P.; Chinaglia, M.; Palma-Silva, C.; Pinheiro, F. Interploidy hybridization in sympatric zones: The formation of *Epidendrum fulgens*× *E. puniceoluteum* hybrids (Epidendroideae, Orchidaceae). *Ecol. Evol.* **2013**, *3*, 3824–3837. http://dx.doi.org/10.1002/ece3.752.

Moraes, A.P.; Simões, A.O.; Alayon, D.I.O.; Barros, F.; Forni-Martins, E.R. Detecting mechanisms of karyotype evolution in *Heterotaxis* (Orchidaceae). *PLoS One* **2016**, *11*, 1–18. https://doi.org/10.1371/journal.pone.0165960.

Moraes, A.P.; Koehler, S.; Cabral, J.S.; Gomes, S.S.L.; Viccini, L.F.; Barros, F.; Felix, L.P.; Guerra M.; Forni-Martins, E.R. Karyotype diversity and genome size variation in Neotropical Maxillariinae orchids. *Plant Biol.* **2017**, *19*, 298–308. https://doi.org/10.1111/plb.12527.

Moraes, A.P.; Engel, T.B.; Forni-Martins, E.R.; Barros, F.; Felix, L.P.; Cabral, J.S. Are chromosome number and genome size associated with habit and environmental niche variables? Insights from the Neotropical orchids. *Ann. Bot.* **2022**, *130*, 11–25. https://doi.org/10.1093/aob/mcac021.

Nollet, F.; Medeiros Neto, E.; Cordeiro, J.M.; Buril, M.T.; Chase, M.W.; Felix, L.P. Chromosome numbers and heterochromatin variation in introgressed and non-introgressed populations of *Epidendrum* (Orchidaceae: Epidendroideae): interspecific transfers of heterochromatin lead to divergent variable karyotypes in the parental populations. *Bot. J. Linn. Soc.* **2022**, *199*, 694–705. https://doi.org/10.1093/botlinnean/boab101.

Oliveira, I.G.; Moraes, A.P.; Almeida, E.M.; Assis, F.N.M.; Cabral, J.S.; De Barros, F.; Felix, L.P. Chromosomal evolution in Pleurothallidinae (Orchidaceae: Epidendroideae) with an emphasis on the genus *Acianthera*: chromosome numbers and heterochromatin. *Bot. J. Linn. Soc.* **2015**, *178*, 102–120. https://doi.org/10.1111/boj.12273.

Oliveira, V.M.; De Barros, F.; Forni-Martins, E.R. Chromosome numbers and karyotypes of Catasetum species (Orchidaceae). *Pl. Biosyst.* **2014**, *148*, 499–507. https://doi.org/10.1080/11263504.2013.788093.

Penha, T.L.L., Corrêa, A.M.; Catharino, E.L.M. Números cromossômicos em *Kleberiella* VP Castro; Cath. (Orchidaceae, Oncidiinae) e gêneros afins. *Acta Bot. Bras.* **2011**, *25*, 466–475.

Querino, B.C.; Ferraz, M.E.; Mata-Sucre, Y.; Souza, G.; Felix, L.P. Cytomolecular diversity of the subtribe Laeliinae (Epidendroidae, Orchidaceae) suggests no relationship between genome size and heterochromatin abundance. *Pl. Syst. Evol.* **2020**, *306*, 1–15. https://doi.org/10.1007/s00606-020-01650-2.

CONSERVATION

Cintra, M.C.S., Lemes, P., Swanni, T.A.; Pessoa, E.M. 2023. Filling the gap to avoid extinction: conservation status of Brazilian species of *Epidendrum* L. (Orchidaceae). *J. Nat. Cons.* **2023**, *71*, 126328.

Endres Júnior, D.; Sasamori, M.H.; Silveira, T.; Schmitt, J.L.; Droste A. Reintrodução de *Cattleya intermedia* Graham (Orchidaceae) em borda e interior de um fragmento de Floresta Estacional Semidecidual no sul do Brasil. *Rev. Bras. Bioci.* **2015**, *13*, 33–40. https://seer.ufrgs.br/index.php/rbrasbioci/article/view/114793.

Fajardo, C.G.; Vieira, F.A.; Felix, L.P.; Molina, W.F. Negligence in the Atlantic forest, northern Brazil: a case study of an endangered orchid. *Biodiv.Cons.* **2017**, *26*, 1047–1063. https://doi.org/10.1007/s10531-016-1285-5.

Fajardo, C.G.; Vieira, F.A.; Molina, W.F. Conservação genética de populações naturais: uma revisão para Orchidaceae. *Biota Amaz.* **2016**, *6*, 108–118.

Nunes, G.P.; Sorgato, J.C.; Ramos, J.C.M.; Soares, J.S.; Rezende, R.K.S.; Peinado, R.B. Innovations in micropropagation of the orchid *Cattleya nobilior*: the effect of bioreactor and enriched culture media. *Ciênc. Rural* **2025**, *55*, e2024027. https://doi.org/10.1590/0103-8478cr20240271.

Oliveira, J.; Moraes, M.C.; Custódio, C.C.; Machado-Neto, N.B. In vitro development and acclimatization of *Cyrtopodium aliciae* L. Linden; Rolfe, an endemic species of the Chapada Diamantina. *Ciênc. Rural* **2023**, *53*, e20210599. https://doi.org/10.1590/0103-8478cr20210599.

Ribeiro, I.S.; Ribeiro, L.M.; Ramos, J.C.M.; Soares, J.S.; Sorgato, J.C. The Brazilian native orchid *Brassavola tuberculata* Hook.: ornamental potential and reintroduction. *Ornam. Hortic.* **2024**, *30*, e242768. https://doi.org/10.1590/2447-536X.v30.e242768.

van den Berg, C. Threats to *Cattleya* in the wild. *Renziana* **2014**, *4*, 80–85.
