# Supplementary material for: An Overview of Orchidaceae from Brazil: Advances and Shortfalls After 400 Years of Studies
Source: Plants (Basel). 2025 Nov 18;14(22):3520. doi: 10.3390/plants14223520 (PMC12656190; doi:10.3390/plants14223520)
Supplement: Supplementary file 1 [file plants-14-03520-s001.zip › plants-3937747-supplementary/Supplementary file S4.pdf]

Supplementary file S4. Checklist of Orchidaceae from Brazilian Pantanal.

Species	Collector and number	Herbarium and barcode
Acianthera pubescens (Lindl.) Pridgeon & M.W.Chase	*Damasceno-Júnior, G.A. s.n.*	COR 3516/UPCB 104501 COR00001075/UPCB0054203
Aspasia variegata Lindl.	*Godinho, R. & Macedo, M. 225*	UFMT15376/ UFMT003506
Bletia catenulata Ruiz & Pavon	*Pott, V.J. et al. 8579*	CGMS68629/ CGMS1101587
Campylocentrum mattogrossense Hoehne	*Hoehne, F.C. 4473*	R3455/ R000003455
Campylocentrum neglectum (Rchb.f. & Warm.) Cogn.	*Damasceno-Júnior, G.A. et al. s.n.*	COR 11972/ COR00105124
Catasetum fimbriatum (C.Morren) Lindl.	*Chaparro, M.A.C et al. s.n.*	SP270027
Catasetum osculatum Lacerda & V.P.Castro	*Andrade, R.P. ADA1234*	UFMT41428
Catasetum rooseveltianum Hoehne	*s. coll.*	LBMBP989
Catasetum saccatum Lindl.	*Bleher, A. s.n.*	SP174986
Cattleya cernua (Lindl.) Van den Berg	*Bortolotto, I. M. et al. s.n.*	COR14143/ COR00001168
Cattleya nobilior Rchb.f.	*Abdo, M.S.A. et al. 1482*	UFMT46757
Cyrtopodium brandonianum Barb.Rodr.	*Godinho, R. & Lopes, I.C. 265*	UFMT15424
Cyrtopodium latifolium Bianch. & J.A.N. Bat.	*Hoehne, F.C. 668*	R3000
Cyrtopodium paludicolum Hoehne	*Pott, V.J. et al. 3067*	CGMS79100/ CGMS1112111/UB0015915
Cyrtopodium poecilum Rchb.f. & Warm.	*Hatschbach, G. et al. 36084*	MBM34455/ MBM034455
Cyrtopodium saintlegerianum Rchb.f.	*Carniello, M.A. et al. 42*	UFMT36087/ HUEFS0178648/ HRCB042062
Cyrtopodium virescens Rchb. f. & Warm.	*Pott, A. et al. 3442*	CGMS79101/ CGMS1112112
Eltroplectris schlechteriana (Porto & Brade) Pabst	*Lozano, E.D. & Hase, L. 958*	MBM405270/ MBM405270
Encyclia argentinensis (Speg.) Hoehne.	*Hoehne, F.C. 632*	R2809
Encyclia linearifolioides (Kraenzl.) Hoehne	*Bueno, N.C. 192*	CPAP6863
Epidendrum anceps Jacq.	*Hoehne, F.C. 445*	R2812
Epidendrum carpophorum Barb. Rodr.	*Hatschbach, G. 33789*	MBM 30881
Epidendrum coronatum Ruiz & Pav.	*Damasceno-Junior, G.A. & Rodriguez, D.P. 1986*	COR13026/ COR00001103
Epidendrum densiflorum Hook.	*Ishii, I.H. et al. 417*	COR13824/ COR00001107
Epidendrum flexuosum G. May	*Hoehne, F.C. 977*	R2830
Epidendrum stiliferum Dressler	*Godinho, R. & M. Macedo 224*	UFMT15387
Epistephium sclerophyllum Lindl.	*Damasceno-Junior, G.A. & Bortolotto, I.M. 1030*	COR5346/ COR00001111
Eulophia alta (L.) Fawc. & Rendle	*Pott, A. et al. 8695*	CGMS69073/ CGMS1102031
Galeandra montana Barb.Rodr.	*Hatschbach, G. et al. 58992*	MBM158589/ MBM158589

Galeandra paraguayensis Cogn.	*Hoehne, F.C. 999*	R193517/
Galeandra styllomisantha (Vell.) Hoehne	*Schessl, M. s.n.*	UFMT23008
Gomesa macropetala (Lindl) M.W.Chase & N.H.Williams	*Lombardi, J.A. 143*	UEC59585/ UEC017524
Habenaria amambayensis Schltr.	*Pott, A. et al. 3318*	CGMS79075/ CGMS1112086
Habenaria anisitsii Kraenzl.	*Pott, A. et al. 7688*	CGMS79079/ CGMS1112108
Habenaria aricaensis Hoehne	*Pott, A. et al. 7675*	CPAP14711
Habenaria glazioviana Kraenzl. ex Cogn.	*Prado, A.L. & Ferreira, M.A. 2607*	UEC138387/ UEC034368
Habenaria gourlieana Gill. ex Lindl.	*Damasceno-Júnior, G.A. et al. 2907*	UPCB104512/ UPCB0054189
Habenaria hamata Barb.Rodr.	*Schessl, M. 3005*	UFMT19888/
Habenaria heptadactyla Rchb.f.	*Cunha, C.N. & Lima-Junior, G.A. 488*	UFMT26986/
Habenaria hexaptera Lindl.	*Damasceno-Júnior, G.A. et al. 3442*	COR12087/ COR00001126
Habenaria juruenensis Hoehne	*Bortolotto, I.M. et al. 175*	COR4161
Habenaria nabucoi Ruschi	*Pott, V.J. & Lima, L.C.P. 4938*	CGMS69936/ CGMS1114279
Habenaria nuda Lindl.	*Ishii, I.H. & Damasceno-Júnior, G.A. 700*	COR12156/ COR00001125
Habenaria orchiocalcar Hoehne	*Pott, A. 2531*	CEN15040/ CEN00015040
Habenaria petalodes Lindl.	*Maciel, A.A. s.n.*	INPA83545/ INPA0083545
Habenaria polycarpa Hoehne	*Damasceno-Júnior, G.A. et al. 3143*	COR13027/ COR00001119
Habenaria pratensis (Lindl.) Rchb.f.	*Costa, S.C. 174*	UFMT24213
Habenaria repens Nutt.	*Rego, S.C.A. 765*	COR922/ COR00001124
Habenaria spathulifera Cogn.	*Pott, A. et al. 7668*	CGMS79447/ CGMS1112458
Ionopsis utricularioides (Sw.) Lindl.	*Abdo, M.S.A. et al. 1421*	UFMT46699/
Isochilus linearis (Jacq.) R.Br.	*Chaparro, M. & Bortolotto, I. M. 07*	COR3488/ COR00001133
Laelia gloriosa Rchb.f.	*Anjos-Silva, E.J. EJAS320*	UFMT18361
Lockhartia goyazensis Rchb.f.	*Amaral, A.M. 142*	UFMT18381
Macradenia paraensis Barb.Rodr.	*Castro, R.M. 1671*	CESJ51010 CESJ051010
Macroclinium mirabile (C.Schweinf.) Dodson.	*Amaral, A.M. 143*	UFMT18402
Maxillaria subrepens (Rolfe) Schuit. & M.W.Chase	*Anjos-Silva, E.J. EJAS328*	UFMT18368
Maxillaria uncata Lindl.	*Fróes, R.L. 33542*	IAN99755/ IAN099755
Microchilus debilis (Lindl.) D.Dietr.	*Pott, A. 4289*	CGMS79095/ CGMS1112106
Microchilus longicornu (Cogn.) E.C.Smidt & M.W.Chase	*Hatschbach, G. et al. 60708*	MBM169330/ MBM169330
Notylia lyrata S.Moore	*Amaral, A.M. 132*	UFMT18405

Notylia odontonotos Rchb.f. & Warm.	*Moore S.M. 387*	BM00092306
Notylia pubescens Lindl.	*Hoehne F.C. 1219*	R3123
Octomeria warmingii Rchb.f.	*Rodriguez, D.P. 07*	COR6546/ COR00001140
Oeceoclades maculata (Lindl.) Lindl.	*Godinho, R. & Macedo, M. 277*	UFMT15402
Orleanesia mineirosensis Garay	*Anjos-Silva, E.J. 317*	UFMT18367
Ornithocephalus cujeticola Barb. Rodr.	*Hoehne, F.C. 891*	R3142
Pelexia stenantha (Cogn.) Schltr.	*Hatschbach, G. 22022*	MBM12742 MBM012742
Polystachya concreta (Jacq.) Garay & Sweet	*Rodriguez, D.P. R-01*	COR6540/ COR00001158
Polystachya foliosa (Hook.) Rchb.f.	*Damasceno-Júnior, G.A. et al. 3144*	COR13031 COR00001162
Prescottia oligantha (Sw.) Lindl.	*Ishii, I.H. et al. 711a*	COR13607 COR00001153
Prosthechea roraimensis V.P.Castro & Campacci	*Hoehne, F.C. 990*	R2821
Pteroglossa macrantha (Rchb.f.) Schltr.	*Pott, V.J. 8554*	CEN64244/ CEN00064244
Sacoila lanceolata (Aubl.) Garay	*Sanches, F.R. s.n.*	ESA007180/ ESA007180
Sarcoglottis curvisepala Szlach. & Rutk.	*Damasceno-Junior, G.A. 1965*	COR13028/ COR00001166
Trichocentrum cepula (Hoffmanns.) J.M.H. Shaw.	*Bortolotto, I.M. et al. s.n.*	COR13109/ COR00001094
Trichocentrum fuscum Lindl.	*Pott, A.& V.J. Pott 13293*	CGMS55646/ CGMS1085613
Trichocentrum jonesianum (Rchb.f.) M.W.Chase & N.H.Williams	*Bortolotto, I.M. et al. s.n.*	COR5387/ COR00001152
Trichocentrum morenoi (Dodson & Luer) M.W.Chase & N.H.Williams	*Pott, A. 12953*	CGMS47972/ CGMS1077930
Trichocentrum nanum (Lindl.) M.W.Chase & N.H.Williams	*Amaral, A.M. 10*	UFMT18392
Trichocentrum pumilum (Lindl.) M.W.Chase & N.H.Williams	*Damasceno-Junior, G.A. et al. 2031*	CGMS84311/ CGMS1118229
Vanilla palmarum (Salzm. ex Lindl.) Lindl.	*Arruda, J.S. 01*	COR4618/ COR00001173
Vanilla phaeantha Rchb.f.	*Novais, G.O. s.n.*	COR1989/ COR00001187
Vanilla pompona Schiede	*Hoehne, F.C. 972*	R2513
Veyretia hassleri (Cogn.) Szlach.	*Prance, G.T. et al. s.n.*	UFMT864
Veyretia simplex (Griseb.) Szlach.	*Schessl, M. 132*	K000878202/ K000878202
Xylobium foveatum (Lindl.) Nichols	*Rodriguez, D.P. 06*	UPCB104505/ UPCB0054186
